# *BCL9L* Dysfunction Impairs Caspase-2 Expression Permitting Aneuploidy Tolerance in Colorectal Cancer

**DOI:** 10.1016/j.ccell.2016.11.001

**Published:** 2017-01-09

**Authors:** Carlos López-García, Laurent Sansregret, Enric Domingo, Nicholas McGranahan, Sebastijan Hobor, Nicolai Juul Birkbak, Stuart Horswell, Eva Grönroos, Francesco Favero, Andrew J. Rowan, Nicholas Matthews, Sharmin Begum, Benjamin Phillimore, Rebecca Burrell, Dahmane Oukrif, Bradley Spencer-Dene, Michal Kovac, Gordon Stamp, Aengus Stewart, Havard Danielsen, Marco Novelli, Ian Tomlinson, Charles Swanton

**Affiliations:** 1 Translational Cancer Therapeutics Laboratory, The Francis Crick Institute, 1 Midland Road, London NW1 1AT, UK; 2 Bioinformatics Science Technology Platform, The Francis Crick Institute, 1 Midland Road, London NW1 1AT, UK; 3 Experimental Histopathology Laboratory, The Francis Crick Institute, 1 Midland Road, London NW1 1AT, UK; 4 Advanced Sequencing Facility, The Francis Crick Institute, 1 Midland Road, London NW1 1AT, UK; 5 Oxford Centre for Cancer Gene Research, The Wellcome Trust Centre for Human Genetics, Roosevelt Drive, Oxford, OX3 7BN UK; 6 Department of Oncology, University of Oxford, Roosevelt Drive, Oxford OX3 7DQ, UK; 7 Translational Cancer Therapeutics Laboratory, University College London Cancer Institute, Paul O'Gorman Building, 72 Huntley Street, London WC2E 6DD, UK; 8 Cancer System Biology, Centre for Biological Sequence Analysis, Department of Systems Biology, Technical University of Denmark, Lyngby 2800, Denmark; 9 Research Department of Pathology, University College London Medical School, University Street, London WC1E 6JJ, UK; 10 Institute for Cancer Genetics and Informatics, Norwegian Radium Hospital, Oslo University Hospital, Ullernchausseen 70, 0379 Oslo, Norway

**Keywords:** aneuploidy tolerance, chromosomal instability, *BCL9L*, caspase-2, p53, intratumor heterogeneity, chromosome segregation errors, mitotic checkpoint, BID, colorectal cancer evolution

## Abstract

Chromosomal instability (CIN) contributes to cancer evolution, intratumor heterogeneity, and drug resistance. CIN is driven by chromosome segregation errors and a tolerance phenotype that permits the propagation of aneuploid genomes. Through genomic analysis of colorectal cancers and cell lines, we find frequent loss of heterozygosity and mutations in *BCL9L* in aneuploid tumors. *BCL9L* deficiency promoted tolerance of chromosome missegregation events, propagation of aneuploidy, and genetic heterogeneity in xenograft models likely through modulation of Wnt signaling. We find that *BCL9L* dysfunction contributes to aneuploidy tolerance in both *TP53*-WT and mutant cells by reducing basal caspase-2 levels and preventing cleavage of MDM2 and BID. Efforts to exploit aneuploidy tolerance mechanisms and the BCL9L/caspase-2/BID axis may limit cancer diversity and evolution.

## Highlights

• Loss-of-function alterations in *BCL9L* are frequent in aneuploid CRC
• *BCL9L* dysfunction drives aneuploidy tolerance by reducing levels of caspase-2
• Caspase-2 activation following aneuploidy results in MDM2 and BID cleavage
• p53 stabilization after chromosome missegregation is regulated by caspase-2

## Graphical Abstract

**Figure:**
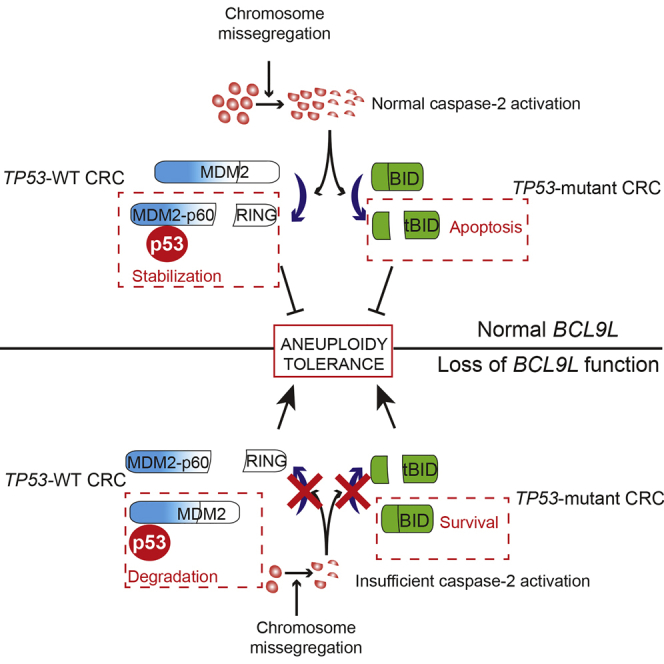

## Teaser

López-García et al. find that *BCL9L* is often genetically inactivated in human colorectal cancers with chromosomal instability. *BCL9L* dysfunction promotes aneuploidy tolerance by reducing basal caspase-2 levels and preventing cleavage of MDM2 and BID independent of *TP53* mutation status.

## Significance

**This comprehensive genomic analysis of aneuploid colorectal cancer identified frequent mutations and deletions of *BCL9L* leading to caspase-2 dysfunction and the tolerance of chromosome missegregation, which operates independently of *TP53* status. These data support the existence of parallel pathways complementing *TP53* dysfunction in the tolerance of aneuploidy and the central role for caspase-2 in the stabilization of p53 following chromosome missegregation events.**

## Introduction

Emerging evidence supports the influence of intratumor heterogeneity on patient outcome and drug response (Ding et al., 2012, Landau et al., 2013, Szerlip et al., 2012). Genomic instability is frequently observed in cancer (Lengauer et al., 1998, Negrini et al., 2010), driving intercellular variation and subsequent intratumor heterogeneity, providing the substrate for selection and tumor evolution (Boutros et al., 2015, Desmedt et al., 2015, Yates et al., 2015).

Chromosomal instability (CIN) is a form of genome instability characterized by the ongoing disorder of chromosome number and/or structure. Numerical CIN occurs after whole chromosome missegregation due to mitotic defects (Bakhoum et al., 2009a, Bakhoum et al., 2009b, Cahill et al., 1998, Sotillo et al., 2007) and results in an aberrant chromosome number, known as aneuploidy. Structural CIN results in the disordered integrity of parts of chromosomes. Both types of CIN are interconnected: missegregated chromosomes are exposed to mitotic stress that generates structural CIN (Crasta et al., 2012, Janssen et al., 2011) while changes in chromosome structure render them susceptible to missegregation (Burrell et al., 2013, Chan et al., 2007, Kawabata et al., 2011, Pampalona et al., 2010).

Since chromosome segregation errors are poorly tolerated by diploid cells (Dewhurst et al., 2014, Iwanaga et al., 2007, Thompson and Compton, 2008, Thompson and Compton, 2010), survival mechanisms, termed aneuploidy tolerance, are crucial for the propagation of aneuploidy in tumors. Mutations in *TP53* (Grim et al., 2012, Li et al., 2010, Thompson and Compton, 2010) and buffering of protein changes due to aneuploidy (Stingele et al., 2013, Torres et al., 2010) have been proposed as candidate mechanisms of aneuploidy tolerance. Due to the potential clinical benefit of limiting CIN in tumors, further efforts to elucidate these survival mechanisms might contribute to limiting this driver of heterogeneity.

Colorectal cancer (CRC) can be broadly divided into microsatellite-instability high (MSI, 20%) and microsatellite-stable tumors (MSS, 80%). MSI CRC tumors remain near diploid, whereas MSS tumors develop a wide range of aneuploid karyotypes and CIN (Bogaert and Prenen, 2014, Cancer Genome Atlas Network, 2012, Mouradov et al., 2013, Rowan et al., 2005). *TP53* mutations occur frequently in aneuploid tumors (Cianchi et al., 1999, Rowan et al., 2005, Tang et al., 2004); however, next-generation sequencing efforts have not specifically explored the somatic mutational landscapes of aneuploid versus diploid MSS CRC tumors to identify determinants of CIN.

In this study, we aimed to identify somatic mutations enriched in aneuploid CRC and to elucidate the potential role of these mutations in the development of CIN in CRC.

## Results

### Somatic Mutation Analysis in CIN Colorectal Cancer Genomes

We selected a cohort of 17 MSS colorectal adenocarcinomas and eight MSS aneuploid cell lines (see Table S1 for clinical features) for whole-exome sequencing. Ploidy status was deduced by calculation of the DNA index (DI) using DNA image cytometry data obtained from nuclei isolated from paraffin-embedded specimens (Table 1 and Figure S1). DI was calculated as the ratio between the mode of the relative DNA content of observable tumor nuclei peaks and a diploid control consisting of nuclei from infiltrated fibroblasts, endothelial cells, and immune cells. To validate the DNA image cytometry results, we performed centromeric fluorescence in situ hybridization (FISH) for chromosomes 2 and 15 (Table 1), since these chromosomes are not frequently subject to whole chromosome gains or losses in CRC (Orsetti et al., 2014). The overall distribution of centromeric signals in tumor cells was compared with the normal adjacent tissue (Table 1; Figures S2A and S2B). By convention, a tumor was classified as aneuploid when an aneuploid peak was detected by DNA image cytometry (DI between 1.1 and 1.89 or greater than 2.1) or when significant changes in the distribution of centromeric signals were detected for one of the chromosomes tested. A tumor was classified as diploid when no aneuploid populations were detected by DNA image cytometry (only one peak with DI = 1) and no significant changes were detected in the distribution of centromeric signals for the two chromosomes tested. We detected modal chromosome signals different from 2 for at least one chromosome in samples with aneuploid peaks (Table 1). For the MSS cell lines, ploidy status was obtained from published karyotyping and SNP array analysis (Table 2, Gaasenbeek et al., 2006, Lee et al., 2011).

Aneuploid CRC is strongly associated with CIN defined by cell-to-cell variation of centromeric signals and surrogate parameters that measure karyotypic complexity in cancer genomes such as the weighted genome instability index (wGII), which assesses the fraction of the genome with alterations (Burrell et al., 2013, Chin et al., 2007). We observed that both modal centromere deviation (MCD) for chromosomes 2 and 15 and the wGII were significantly higher in aneuploid tumors (Figures S2C and S2D). In samples with DI = 1, the modal centromere signal was 2 in all cases except tumor 395, which showed significant alterations in the overall distribution of chromosome 15 signals and was therefore classified as aneuploid (Table 1 and Figure S2E). This classification was also supported by a high wGII (0.41). No significant differences were observed in tumor sample purity between aneuploid and diploid tumors (Table 1 and Figure S2F). Taken together, ten MSS tumors were classified as aneuploid and seven as diploid (Table 1).

**Figure 1:**
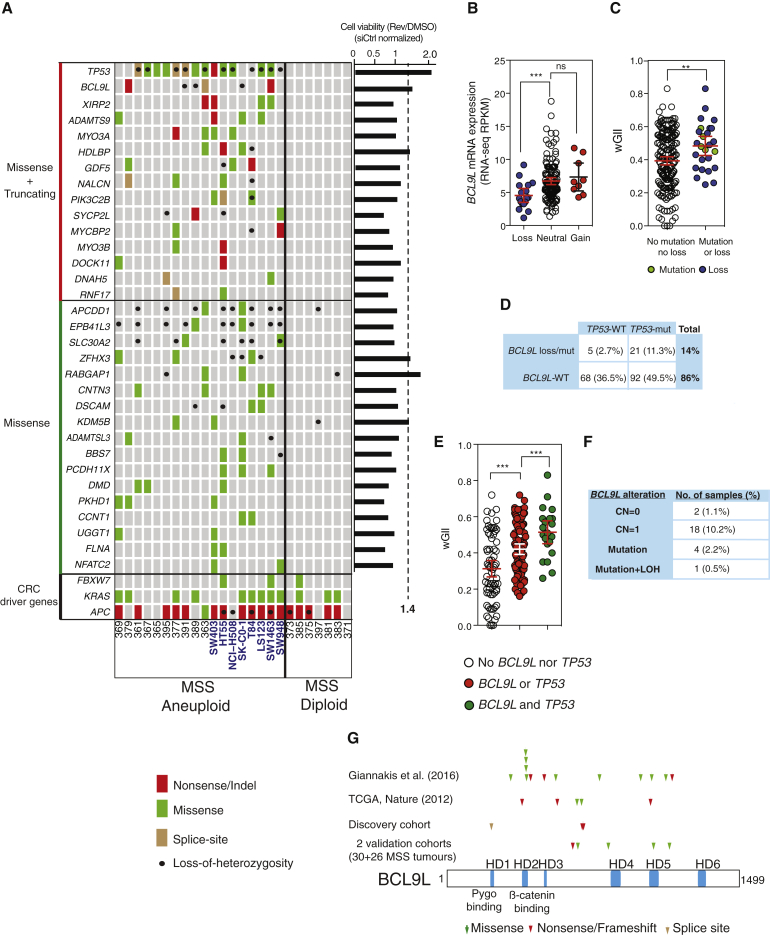
Genomic Analysis of Aneuploid CRC and Cell Lines (A) Non-synonymous somatic mutations and loss of heterozygosity in a discovery cohort of 17 MSS CRC tumors and eight cell lines (in blue). Genes mutated in aneuploid samples are shown separated in two categories (truncating + missense or missense only). Common CRC driver genes are shown at the bottom. Right-hand graph presents the results of the reversine tolerance screen. Each value was normalized to siControl (siCtrl) treated cells, and genes whose knockdown provided a ≥1.4-fold increase in viability were considered for further analysis. (B) Correlation between *BCL9L* copy number and mRNA level in MSS CRC in the TCGA dataset. Loss (CN = 0 or 1), neutral (CN = 2), and gain (CN ≥ 3). RPKM, reads per kilobase of transcript per million mapped reads; p values were calculated by unpaired Student's t test. (C) wGII in MSS CRC TCGA samples with *BCL9L* alterations and wild-type *BCL9L* (p values calculated by unpaired Student's t test). (D) Co-occurrence and mutual exclusivity of *BCL9L* alterations and *TP53* mutations in TCGA MSS CRC (percentage of all MSS samples). (E) wGII in samples with mutually exclusive and co-occurring *BCL9L* and *TP53* somatic alterations (MSS CSC TCGA; p values calculated by unpaired Student’s t test). (F) Allelic pattern of *BCL9L* alterations in TCGA MSS CRC (percentage of all MSS samples). (G) Mapping of non-synonymous mutations across the BCL9L protein identified in four different cohorts. Error bars denote 95% confidence interval. ns, not significant. ^∗∗^p < 0.01, ^∗∗∗^p < 0.001. See also Figure S3 and Tables S2–S4.

To attempt to identify aneuploidy-specific mutations, we performed exome sequencing on DNA from tumors, normal adjacent tissue, and cell lines (Table S2), and mutation calling of tumor somatic variants was performed by filtering germline variants identified in normal adjacent colon. Manual curation of variant calls and validation by Sanger sequencing revealed a list of 32 genes specifically mutated in aneuploid samples (Figure 1A and Table S3). Notably, known CRC drivers (*APC*, *FBXW7*, and *KRAS*) did not segregate according to tumor ploidy status. As expected, however, *TP53* mutations were significantly enriched in aneuploid tumors (Figure 1A, 13/18 aneuploid tumors and cell lines, 0/7 diploid samples; p = 0.001, Fisher's exact test). No somatic mutations were mutually exclusive with *TP53* in this discovery cohort.

*BCL9L* was the only gene for which all mutations found were clearly inactivating, with one nonsense mutation Q713^∗^ in tumor 379, one nonsense mutation R716^∗^ in the cell line SW1463, and one splice-site variant (exon 5 + 1 G > A) in the tumor sample 363. We also found loss of heterozygosity (LOH) at the *BCL9L* locus in two aneuploid tumors (391 and 389) and one cell line (SK-CO-1). R716^∗^ was observed in two of four alleles of the aneuploid cell line SW1463. Q713^∗^ and the splice-site mutation were observed in two of three alleles of tumor samples 379 and 363, respectively, which suggests that *BCL9L* mutations occurred early, prior to chromosome duplication.

Next, we performed two functional RNAi screens probing phenotypes relevant to chromosome segregation errors and their tolerance. We used the diploid cell line HCT-116 due to its low level of constitutive chromosome segregation errors relative to CIN CRC cell lines and its poor tolerance of drug-induced segregation errors (Gascoigne and Taylor, 2008, Lengauer et al., 1997, Thompson and Compton, 2010). In the first RNAi screen we silenced each of the 32 genes mutated in aneuploid samples and examined the consequence upon chromosome segregation error frequency as previously described (Burrell et al., 2013), with no significant results. Second, we performed a screen to detect tolerance of chromosome segregation errors. Chromosome missegregation induces p53-mediated cell cycle arrest in the next G1 phase, often followed by apoptosis, thereby preventing propagation of aneuploid progeny (Figure S3A; see also Hinchcliffe et al., 2016). Chromosome missegregation can be artificially induced in HCT-116 cells with reversine, an Mps1 inhibitor that impairs the spindle assembly checkpoint resulting in chromosomal non-disjunction and missegregation. Consistent with results by Jemaa et al. (2012), we found that reversine treatment induces subsequent arrest or cell death (Figure S3A). We depleted all aneuploid-specific genes (Figure 1A) individually in HCT-116 cells with small interfering RNA (siRNA) pools from Dharmacon in the presence or absence of 250 nM reversine, a concentration that does not inhibit Aurora kinase B (Santaguida et al., 2010). Silencing of *TP53*, *RABGAP1*, *BCL9L*, *HDLBP*, and *ZFHX3* induced reversine tolerance (Figure 1A, right). We considered a gene validated when at least three of four individual oligonucleotides (components of the pool of four) showed the same effect as the pool. *TP53*, *BCL9L*, and *ZFHX3* were validated following these criteria (Figure S3B). Experiments with distinct Qiagen siRNA pools also showed a similar result for *TP53*, *BCL9L*, and *ZFHX3* (Figure S3B).

Efficient depletion of the BCL9L protein was observed for all four single oligonucleotides (Figure S3C); however, we discarded the BCL9L oligonucleotide 4 due to high cellular toxicity. Finally, expression of a BCL9L-EGFP construct lacking the 3′ UTR region reverted the survival phenotype in reversine when an siRNA targeting 3′ UTR was transfected, further supporting that aneuploidy tolerance observed with various siRNA duplexes is due to on-target silencing of *BCL9L* (Figure S3D). *BCL9L* silencing also increased cell viability following treatment with 200 nM aphidicolin (Figure S3E), which causes replication stress and induces segregation errors of structurally unstable chromosomes (Burrell et al., 2013). No tolerance effect was observed when HCT-116 cells were treated with doxorubicin, suggesting that silencing of *BCL9L* does not result in general resistance to cytotoxics causing DNA damage (Figure S3F).

Given the aneuploid-specific pattern of LOH and truncating events of *BCL9L*, the second most commonly truncated gene in aneuploid CRC after *TP53* in our discovery cohort (Figure 1A and Table S3), and its putative aneuploidy tolerance function in the siRNA screen, we investigated *BCL9L* somatic events in independent cohorts. We analyzed data from 186 MSS CRCs available from The Cancer Genome Atlas (TCGA) (Cancer Genome Atlas Network, 2012). *ZFHX3* was not investigated further as mutations in this gene were not enriched in aneuploid CRC in validation cohorts.

We confirmed that samples with somatic copy-number loss of *BCL9L* had significantly lower gene expression compared with samples with no alterations in *BCL9L* (Figure 1B). Using the wGII score as a surrogate of chromosomal instability and aneuploidy in CRC as previously described (Chin et al., 2007, Lee et al., 2011), we observed significantly higher wGII scores in tumors harboring *BCL9L* mutation or copy-number loss compared with tumors with no alteration in *BCL9L* (Figure 1C). This relationship remained significant when controlling for the higher probability of gene loss in high-wGII tumors (Figure S3G, p = 0.03).

The majority of *BCL9L* deletions and mutations co-occurred with *TP53* mutations (21/26 tumors with co-occurrence, Figure 1D). In a similar computational permutation analysis as performed above (Figures 1C and S3G), samples with co-occurring *BCL9L* and *TP53* alterations displayed higher wGII scores compared with those with mutually exclusive alterations (Figure 1E, p = 0.007), suggesting that these two genes might cooperate as aneuploidy suppressors in CRC.

Comprehensive genomic analysis from the same colorectal TCGA cohort enabled us to infer the genotype of *BCL9L* alterations in MSS CRC (Figure 1F). In total, *BCL9L* mutations and/or deletions occurred in 14% of MSS CRC (26/186, 5 mutations and 21 deletions), the majority of which (23/26) retained a wild-type (WT) copy of *BCL9L* while biallelic alterations of *BCL9L* occurred in only three samples. Taken together, these results suggest a haploinsufficient model of tumor suppression for *BCL9L*.

Finally, we evaluated the pattern of *BCL9L* non-synonymous mutations in MSS CRC across the BCL9L protein (Figure 1G). We compiled all *BCL9L* mutation data from the discovery cohort in Figure 1A, the TCGA MSS CRC cohort, a large cohort of 438 MSS CRC published by Giannakis et al. (2016) together with two additional validation cohorts of MSS CRC tumors sequenced by Ion Torrent targeted sequencing (Figure S3H). The 27 *BCL9L* somatic mutations identified were scattered across the gene with one cluster of four missense mutations mapping to two adjacent residues within the β-catenin binding HD2 domain (R409W and S410F). Thirty-seven percent (10/27) of the somatic mutations were truncating events (nonsense, splice-site, and indel mutations) whereas 17 of 27 were missense mutations (see Table S4 for functional impact). This characteristic profile of scattered mutations with >20% of inactivating/truncating mutations is consistent with the tumor-suppressor pattern proposed by Vogelstein et al. (2013). Consistent with these data, comprehensive computational analysis has classified *BCL9L* as a candidate driver gene in a pan-cancer analysis (Tamborero et al., 2013) and as a significantly mutated driver gene in MSS CRC (Giannakis et al., 2016).

### Loss of *BCL9L* Drives Tolerance to Segregation Errors and Aneuploidy

**Figure 2:**
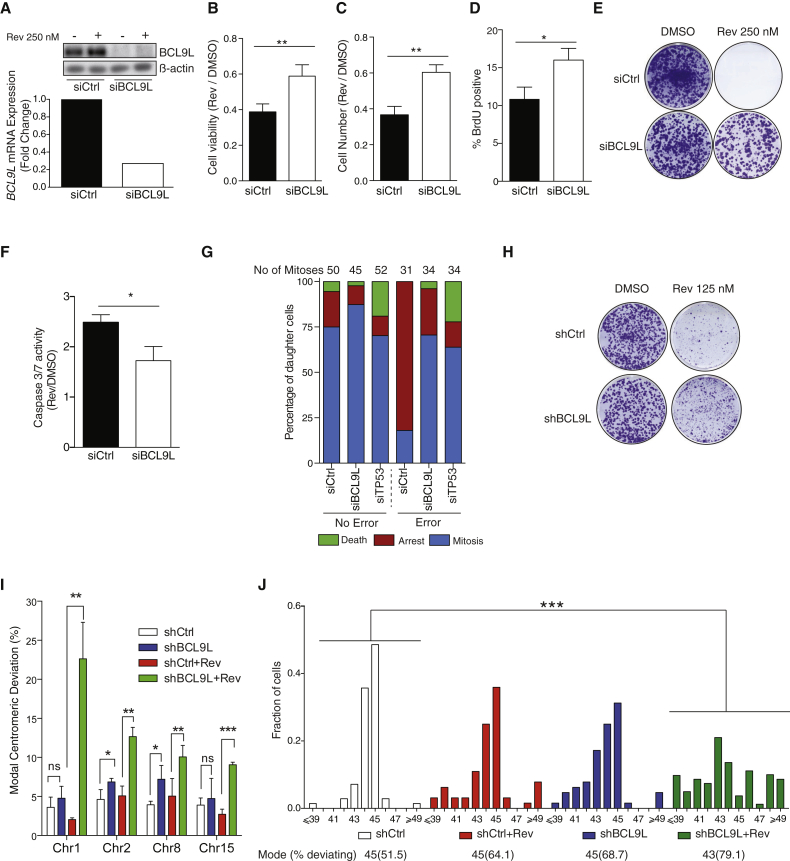
BCL9L Knockdown Confers Aneuploidy Tolerance in HCT-116 Cells (A) siRNA knockdown of BCL9L protein (top) and mRNA (bottom) in HCT-116 (representative experiments shown, Qiagen siRNA pool). (B–D) Impact of 250 nM reversine treatment on cell viability (B), cell number (C), and fraction of BrdU-incorporating cells (D) following control or BCL9L siRNA transfection (72 hr, n = 3; p values calculated by paired Student's t test). (E) Colony-forming efficiency of HCT-116 after siRNA transfection and 250 nM reversine treatment. (F) Fold change induction of Caspase-3/7 enzymatic activity following 250 nM reversine treatment in HCT-116 transfected control siRNA or after BCL9L knockdown (72 hr, n = 3, p value calculated by paired Student's t test). (G) Live-cell imaging analysis of post-mitotic daughter cell fate after a normal mitosis or mitosis with endogenous segregation errors. Daughter cells were tracked for at least 48 hr. Arrest was defined as the absence of cell division within 48 hr post mitosis. Death was defined as visible nuclear collapse. (H) Effect of stable lentiviral BCL9L knockdown on colony-forming efficiency of HCT-116 treated with 125 nM reversine for 15 days and recovered for additional 15 days. (I) Modal centromeric deviation (%) in the number of FISH signals (CEP 1, 2, 8, and 15) in reversine-treated and untreated HCT-116 cells with lentiviral stable BCL9L knockdown (n = 3, 400–500 nuclei scored per experiment). Cells were treated as in (H). (J) Karyotypic analysis following stable lentiviral BCL9L knockdown and reversine treatment. Metaphases were stained with a pan-centromeric probe (70–100 cells analyzed, modal number of chromosomes and percentage deviating from the mode shown at the top; p values calculated by two-sided Wilcoxon rank-sum test). Error bars denote SD. ns, not significant. ^∗^p < 0.05, ^∗∗^p < 0.01, ^∗∗∗^p < 0.001. See also Figures S4 and S5.

The results shown above prompted us to carry out a more detailed study of the role of *BCL9L* dysfunction in aneuploidy tolerance. The diploid cell line HCT-116 expresses high levels of BCL9L, and siRNA transfection efficiently depleted BCL9L protein and mRNA (Figure 2A). *BCL9L* silencing increased the number of metabolically active cells, total cell number, bromodeoxyuridine (BrdU)-incorporating cells, and colony-forming efficiency in reversine-treated HCT-116 cells (Figures 2B–2E) and reduced reversine-induced apoptosis (Figure 2F). In the absence of reversine, *BCL9L* silencing did not induce any significant changes in cell proliferation or apoptosis, nor did it affect the rate of constitutive segregation errors (Figure S4A). BCL9L knockdown also induced reversine tolerance in a panel of near-diploid colorectal cell lines that express BCL9L (Gaasenbeek et al., 2006, Lee et al., 2011; Figures S4B–S4D). In contrast, survival of *BCL9L* mutant and/or non-expressing cells (LS-174T and RKO) in reversine was not improved after *BCL9L* silencing, suggesting on-target specificity of the BCL9L siRNAs (Figures S4B–S4D; see also COSMIC database at cancer.sanger.ac.uk).

Next, we examined the fate of daughter cells arising from error-free mitoses or mitoses with naturally occurring segregation errors. For this we used HCT-116 cells expressing H2B-RFP to visualize chromosomes. Following control siRNA transfection, the majority of daughter cells that had undergone a chromosome segregation error did not divide again within 48 hr (Figures 2G and S5A, and Movies S1, S2, S3, and S4 for examples of endogenous segregation errors). Longer-term observation of cells that had undergone a chromosome segregation error revealed that 24.5% of arrested cells died between 48 and 72 hr after mitosis (Figure S5B). In contrast, the majority of daughter cells entered a second mitosis following silencing of *BCL9L* or *TP53*, whether an endogenous segregation error occurred or not (Figures 2G and S5A). Similar results were found through live-cell microscopy of three additional cell lines (Figure S5C). These results suggest that *BCL9L* dysfunction promotes survival following chromosome segregation errors by a mechanism that may not be unique to Mps1 inhibition but a more general mechanism that also applies to endogenous chromosome segregation errors.

We generated HCT-116 cells with partial depletion of BCL9L (Figure S5D) using a lentiviral small hairpin RNA (shRNA) vector to study the long-term consequences of *BCL9L* silencing. Treatment with 125 nM reversine for 15 days revealed an increase in colony-forming efficiency in the shBCL9L cells relative to shControl (shCtrl) cells (Figure 2H). To study whether BCL9L depletion promotes the propagation of aneuploid cells, we treated cells with 125 nM reversine for 15 days followed by a 2-week recovery in drug-free medium, and performed centromeric FISH analysis with centromeric probes for four chromosomes (Figure 2I). In untreated cells, *BCL9L* silencing produced a small but significant increase in the modal centromeric deviation when compared with shCtrl for chromosomes 2 and 8. In shBCL9L cells pre-treated with reversine, this increase was significant for all four probes (Figure 2I). Total chromosome counts carried out on metaphase spreads derived from the same cells supported the development of aneuploidy in BCL9L-depleted cells treated with reversine (Figure 2J). We did not detect structurally aberrant chromosomes in metaphase spreads. There was no evidence of cytokinesis failure resulting in tetraploidization in BCL9L-depleted cells treated with reversine (Figure S5E).

**Figure 3:**
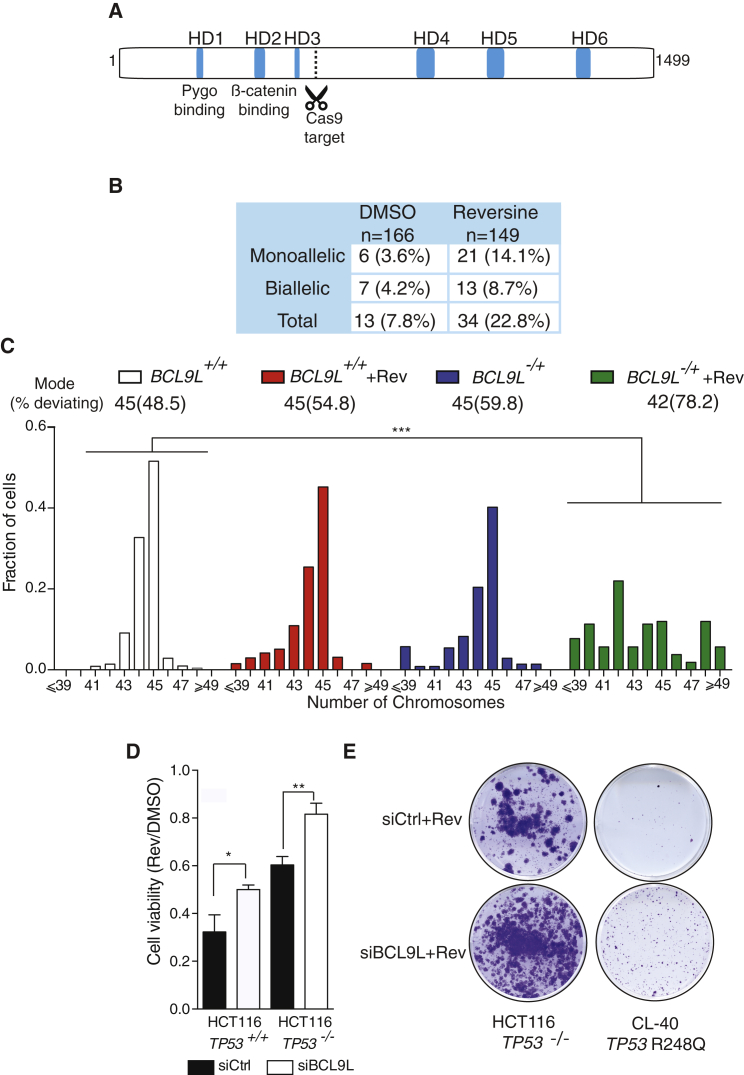
Heterozygous Truncation of *BCL9L* Drives Aneuploidy Tolerance in HCT-116 Cells (A) Mapping of the CRISPR protospacer site on the BCL9L protein. Guide RNA targets nucleotides 2,542–2,561 (*BCL9L* cDNA sequence GenBank: NM_182557). (B) Genotyping results of *BCL9L* CRISPR clones selected in 125 nM reversine for 15 days (percentage of all isolated clones). (C) Karyotypic analysis of an isolated HCT-116 clone bearing a heterozygous 5-bp deletion C-terminal to the HD3 domain in *BCL9L* (p.Glu530fs) (70–100 cells analyzed, modal number of chromosomes and percent deviating from the mode shown above the graph; p value calculated by two-sided Wilcoxon rank-sum test). (D) Effect of BCL9L depletion on cell viability of isogenic *TP53*-WT and null HCT-116 cells (72 hr, n = 3; p value calculated by paired Student's t test). Cell viability was measured by CellTiter-Blue (Promega). (E) Colony-formation assay in CRC *TP53*-mutant cells (*TP53*-null HCT-116 and CL-40). Error bars denote SD. ^∗^p < 0.05, ^∗∗^p < 0.01, ^∗∗∗^p < 0.001. See also Figure S5.

Next, we engineered *BCL9L* truncating mutations in HCT-116 cells similar to those observed in CRC using CRISPR/Cas9. Since most truncating *BCL9L* mutations preserve HD1, HD2, and HD3 domains (Figure 1G), we designed a guide RNA targeting *BCL9L* C-terminal to the HD3 domain (Figure 3A). Sanger sequencing of clones selected in 125 nM reversine for 2 weeks showed a 2.9-fold enrichment in *BCL9L* mutant clones selected in reversine when compared with untreated colonies (Figure 3B). Both monoallelic and biallelic *BCL9L* truncations appeared to be selected by reversine treatment (Figure 3B). Karyotypic analysis of metaphase spreads of HCT-116 with a heterozygous 5-bp deletion C-terminal to the HD3 domain generated by CRISPR/Cas9 (reduction of BCL9L protein shown in Figure S5F) showed an increase in aneuploidy in reversine-treated *BCL9L*^−/+^ cells in comparison with the WT control (Figure 3C). Taken together, these results with *BCL9L* mutant cell lines and our genomic analysis support a role for *BCL9L* haploinsufficiency conferring aneuploidy tolerance.

The majority of CRCs with *BCL9L* alterations also harbor *TP53* mutations and this co-occurrence seems to coincide with higher wGII scores in tumors (Figures 1D and 1E). Silencing of *BCL9L* in HCT-116 *TP53*-null cells also increased the fraction of surviving cells after 3 days of reversine treatment (Figure 3D) and the number of resistant colonies in HCT-116 *TP53*-null and CL-40 cells, a CRC cell line harboring the most frequent *TP53* mutation in CRC (R248Q, Figure 3E). These data support the hypothesis that BCL9L depletion results in an additive survival effect in *TP53-*mutant cells and suggest that loss of *BCL9L* contributes to aneuploidy tolerance in both *TP53*-competent and mutant CRC.

### Effect of *BCL9L* Loss on Xenograft Models of Tumorigenesis

**Figure 4:**
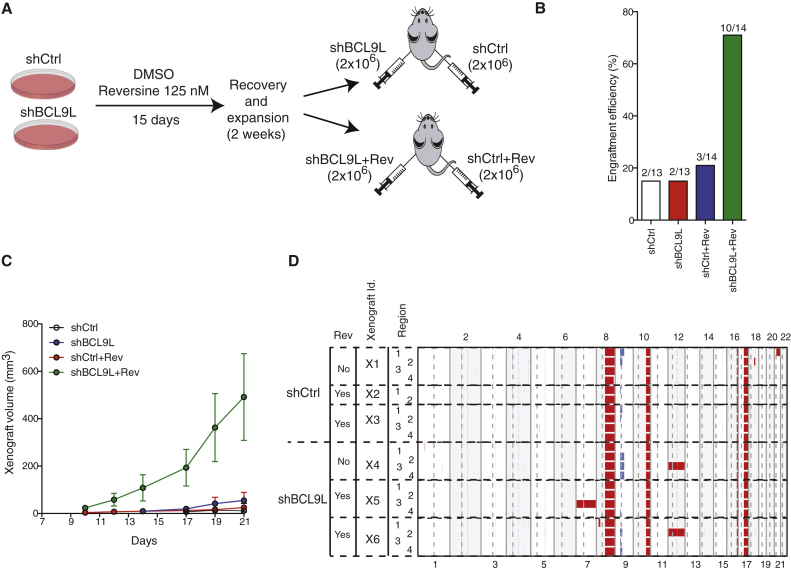
BCL9L Depletion Results in Intratumor Heterogeneity in Xenografts (A) Experimental procedure for xenograft experiments. Stable shBCL9L HCT-116 cells were treated as in Figure 2H. Cells (2 × 10^6^) were subcutaneously injected in each mouse flank. (B) Engraftment efficiency of cells treated as described in (A) 60 days following injection. (C) Growth curves of shCtrl and shBCL9L xenografts with and without reversine pre-treatment (mean ± SEM). (D) Genome-wide multi-region SNP DNA copy-number analysis of six xenografts (three shCtrl and three shBCL9L). Red, gain; blue, loss.

To determine the role of *BCL9L* as an aneuploidy suppressor in vivo, we injected BCL9L-depleted or control cells into immunocompromised mice following the protocol shown in Figure 4A. We observed that reversine pre-treatment of shBCL9L cells dramatically improved the engraftment efficiency and growth rate when compared with the rest of the experimental situations (Figures 4B and 4C). Although untreated shBCL9L cells did not engraft better, they displayed a modest growth advantage when compared with untreated shCtrl cells, although these differences were not statistically significant (Figure 4C).

We hypothesized that the increased karyotypic diversity in *BCL9L*-deficient cells pre-treated with reversine (Figures 2J and 3C) might lead to clonal selection of advantageous karyotypes, promoting intratumor heterogeneity of whole chromosome aneuploidies in the mouse xenografts. SNP profiling of the xenografts (Figure 4D) detected ubiquitous alterations on chromosomes 8, 10, 16, and 17 that are known for parental HCT-116 cells, together with intratumor heterogeneity for whole chromosome 12 in one region of two BCL9L-depleted xenografts and whole chromosome 7 gain in one region of one BCL9L-depleted xenografts. Notably, whole chromosome gains were observed in shBCL9L cells both with and without reversine, substantiating the role of *BCL9L* loss in the tolerance and propagation of endogenous segregation errors. Control cells did not show any heterogeneous whole chromosome alterations. Gain of the long arm of chromosome 21 was seen in one region of one shCtrl xenograft. These data support the ability of BCL9L depletion to foster intratumor heterogeneity and the propagation of subclones with whole chromosome aneuploidies distinct from other subclones within the same tumor.

### Mechanism of *BCL9L*-Mediated Aneuploidy Surveillance

**Figure 5:**
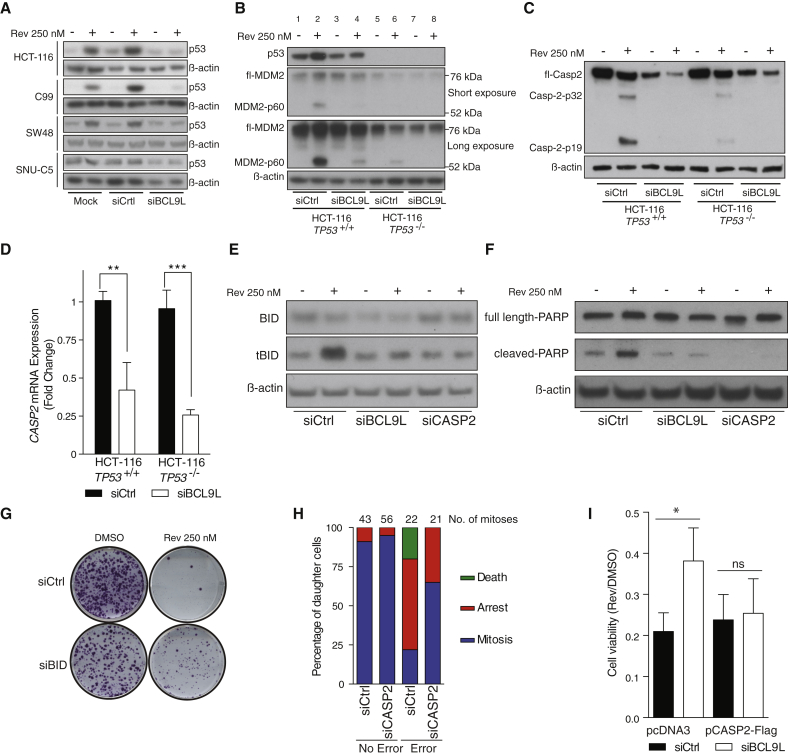
Mechanisms of *BCL9L*-Mediated Aneuploidy Tolerance (A) Effect of BCL9L depletion on p53 protein levels in four near-diploid CRC cell lines following reversine treatment for 72 hr. (B) MDM2 protein expression and cleavage in reversine-treated (72 hr) *TP53*-WT and *TP53*-null HCT-116 cells following BCL9L depletion (two exposures shown). (C) Effect of BCL9L depletion on caspase-2 protein expression and cleavage in isogenic HCT-116 cells (72 hr). (D) qPCR analysis of caspase-2 mRNA in BCL9L-depleted *TP53*-WT and *TP53*-null HCT-116 cells (n = 3; p value calculated by unpaired Student's t test). (E) BID protein expression and cleavage (tBID) in reversine-treated *TP53*-null HCT-116 cells following BCL9L or caspase-2 depletion (72 hr). (F) Effect of BCL9L and caspase-2 depletion on PARP cleavage in *TP53*-null HCT-116 cells (72 hr). (G) Effect of *BID* silencing on colony-forming efficiency in reversine-treated *TP53*-null HCT-116 cells. (H) Live-cell imaging analysis of daughter cell fate after normal mitosis and mitosis with endogenous segregation errors in caspase-2-depleted HCT-116 cells. (I) Viability of cells at 72 hr after co-transfecting pcDNA3-caspase-2-FLAG or empty control together with the indicated siRNA in the presence and absence of 250 nM reversine (n = 3; p value calculated by paired Student's t test). Error bars denote SD. ns, not significant. ^∗^p < 0.05, ^∗∗^p < 0.01, ^∗∗∗^p < 0.001. See also Figures S6 and S7.

p53 stabilization mediates apoptosis and cell cycle arrest upon genotoxic stress. Western blot analysis showed that *BCL9L* silencing strongly inhibited p53 accumulation following reversine treatment in *TP53*-WT HCT-116, SW48, and C99 cells, an effect reproduced with three siRNA duplexes (Figures 5A and S6A). We did not detect p53 accumulation in *TP53*-mutant SNU-C5 cells upon reversine treatment (Figure 5A). *BCL9L* silencing in HCT-116 did not affect *TP53* mRNA levels (Figure S6B). However, *BCL9L* silencing in HCT-116 inhibited the induction of the p53 transcriptional targets *CDKN1A* (p21) and *BBC3* (Puma) after reversine treatment (Figure S6C).

We then examined MDM2 expression due to its important role in regulating p53 stability (Karni-Schmidt et al., 2016). Following reversine treatment, we observed an intense band around 60 kDa similar to the MDM2-p60 N-terminal cleavage product previously described (Oliver et al., 2011) (Figure 5B) that was not detected by C-terminal MDM2 antibodies (Figure S6D). MDM2-p60 accumulated mainly in the nucleus where it co-localized with p53 (Figure S6E). Importantly, MDM2 cleavage was still detectable in *TP53-*null cells following reversine exposure, and was impaired following *BCL9L* silencing and reversine treatment in both *TP53*-WT (Figure 5B, compare lanes 2 and 4) and *TP53*-null cells (Figure 5B, compare lanes 6 and 8).

Active caspase-2 cleaves MDM2, generating the MDM2-p60 fragment as part of a p53 regulatory cascade. MDM2-p60 conserves the p53 binding domain but is devoid of the RING domain. p60-p53 heterodimers cannot be targeted for degradation, which ultimately enhances p53 accumulation (Oliver et al., 2011, Terry et al., 2015). We observed that reversine treatment induced cleavage of caspase-2 (determined by cleavage of caspase-2 into p32 and p19 moieties) in both HCT-116 *TP53*-WT and to a lesser extent in *TP53*-null cells (Figure 5C). We also observed reduced levels of caspase-2 protein and mRNA in BCL9L-depleted HCT-116 *TP53*-WT and null cells (Figures 5C, 5D, and S5D), which contributed to lower levels of active caspase-2 upon reversine treatment. A reduction in caspase-2 protein following *BCL9L* silencing was also confirmed in other cell lines (Figure S6F) and with different siRNA sequences targeting *BCL9L* (Figure S6G). qPCR analysis revealed that reversine treatment did not increase the expression of *PIDD* mRNA, a gene involved in p53-dependent caspase-2 activation (Figure S6H). Cell synchronization and transient reversine exposure revealed that p53 stabilization, MDM2 cleavage, and caspase-2 activation are detectable after one division in the presence of reversine (doubling time for HCT-116 = 20 hr), confirming that one cell division is sufficient to trigger these three events (Figure S6I). Similar to *BCL9L* silencing, caspase-2 depletion by RNAi attenuated both p53 accumulation and MDM2 cleavage upon reversine treatment (Figures S7A and B).

These results suggest that reversine induces proteolytic activation of caspase-2 partially independent of p53, and depletion of BCL9L reduces caspase-2 expression that ultimately prevents cleavage of MDM2 and stabilization of p53 following reversine exposure.

Given the higher number of karyotypic alterations in CRC with co-occurrence of *BCL9L* and *TP53* alterations (Figure 1E) and our results showing a *BCL9L* survival effect in *TP53*-WT and null backgrounds (Figures 3D, 3E, S4C, and S4D), we investigated a potential p53-independent role for BCL9L in aneuploidy tolerance. Since caspase-2 cleavage was detectable, but at reduced levels, in *TP53*-null cells (Figure 5C), we assessed the role of other caspase-2 substrates, such as BID, in mediating aneuploidy tolerance (Guo et al., 2002). Although *BCL9L* silencing in *TP53*-null cells resulted in lower levels of basal *BID* mRNA (Figure S7C), only a moderate reduction in BID protein steady-state levels was observed (Figures 5E and S7D). In *TP53*-null cells, reversine treatment induced formation of a 15 kDa band consistent with tBID (Figures 5E and S7D), derived through caspase-mediated cleavage of BID. Silencing of either *BCL9L* or caspase-2 attenuated this cleavage (Figures 5E and S7D). tBID relocalizes to the outer mitochondrial membrane where it activates the mitochondrial apoptotic pathway (Korsmeyer et al., 2000). Consistent with a p53-independent pro-apoptotic role for BCL9L, BCL9L and caspase-2 depletion prevented poly(ADP-ribose)polymerase (PARP) cleavage in reversine-treated *TP53-*null HCT-116 cells (Figure 5F). Colony-forming assays confirmed that BID depletion by siRNA had a similar effect to *BCL9L* silencing in reversine-treated HCT-116 *TP53*-null cells (Figure 5G). We did not find significant changes in the expression of other caspases and mitochondrial apoptotic regulators (Figure S7E).

Consistent with the results shown above, caspase-2 depletion increased resistance to reversine treatment in BrdU incorporation assays (Figure S7F), and also increased tolerance to endogenous segregation errors in HCT-116 cells (Figure 5H). Finally, co-transfection of siRNA targeting BCL9L and a caspase-2 expression plasmid (Figure S7G) reverted the tolerance of reversine treatment mediated by BCL9L depletion (Figure 5I). These observations support a mechanism of aneuploidy tolerance whereby caspase-2 suppression in BCL9L-depleted cells enhances the survival of cancer cells after endogenous or drug-mediated segregation errors in both *TP53*-WT and *TP53*-null backgrounds.

**Figure 6:**
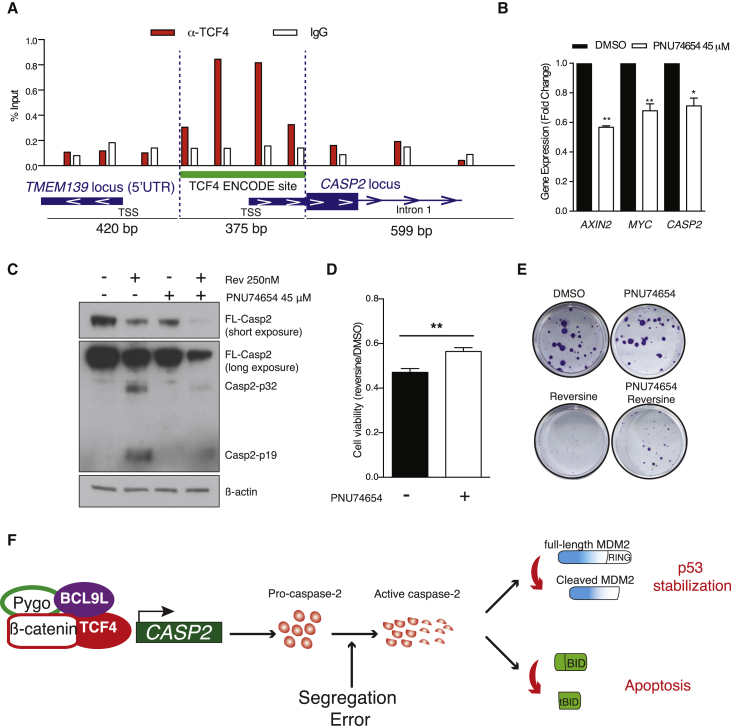
Mechanism of *BCL9L* in the Regulation of Caspase-2 Expression (A) Chromatin immunoprecipitation of TCF4 and qPCR analysis of the immunoprecipitated DNA. Primers were designed across the caspase-2 promoter region that contains a TCF4 binding site annotated in ENCODE (bars are located approximately in the center of the resulting amplicon). TSS, transcription start site; IgG, immunoglobulin G. (B) mRNA expression analysis of caspase-2 and the Wnt targets *AXIN2* and *MYC* following treatment with 45 μM PNU74654 (n = 3; p values calculated by unpaired Student's t test). (C) Effect of PNU74654 on caspase-2 protein with and without reversine treatment. (D) Effect of PNU74654 on cell viability following reversine treatment in HCT-116 cells (mean ± SD, n = 3). (E) Colony-forming efficiency of HCT-116 cells treated with reversine in the presence or absence of 45 μM PNU74654. (F) Proposed mechanism of *BCL9L* in aneuploidy surveillance. Error bars denote SD. ^∗^p < 0.05, ^∗∗^p < 0.01. See also Figure S8.

Next, we explored the hypothesis that *BCL9L* loss drives aneuploidy tolerance through repression of Wnt signaling. BCL9/BCL9L and their binding partners β-catenin and Pygo function as transcriptional co-activators that facilitate the activity of the TCF/LEF family of transcription factors (de la Roche et al., 2008). We confirmed that *BCL9L* silencing inhibited TCF4 transcriptional activity in reporter assays and expression of Wnt signaling targets (Figures S8A and S8B). Examination of the ENCODE database (Rosenbloom et al., 2012) revealed a potential TCF4-binding site near the transcription start site of *CASP2* that we were able to confirm by TCF4 chromatin immunoprecipitation in HCT-116 cells (Figure 6A). Treatment of HCT-116 with PNU74654, a drug that inhibits Wnt signaling by impairing β-catenin binding to TCF4, triggered a statistically significant downregulation of the Wnt targets *AXIN2* and *MYC* along with reduction of caspase-2 mRNA and protein (Figures 6B and 6C). In addition, treatment of HCT-116 cells with PNU74654 induced reversine tolerance relative to HCT-116 cells treated with reversine alone (Figures 6D and 6E).

In summary, we propose a model in which partial loss of *BCL9L* results in lower caspase-2 mRNA and protein levels in both *TP53*-WT and mutant cells, likely mediated through inhibition of TCF4 transcriptional activity at the *CASP2* promoter. After chromosome segregation errors, fully functional BCL9L permits transcription and activation of caspase-2, resulting in p53 stabilization via MDM2 cleavage in *TP53*-WT cells and BID cleavage in *TP53*-mutant cells, ultimately inducing arrest and apoptosis (Figure 6F). In cancer cells, *BCL9L* dysfunction results in lower levels of caspase-2, and when chromosome missegregation occurs this deficiency results in suboptimal activation of caspase-2, leading to impaired p53 stabilization, tBID formation, and attenuated cell death.

## Discussion

Aneuploidy has prognostic relevance in multiple cancer types (Carter et al., 2006, Danielsen et al., 2016, Kronenwett et al., 2004, Mouradov et al., 2013, Yoo et al., 2010) and is associated with cancer multidrug resistance (Kuznetsova et al., 2015, Lee et al., 2011, Swanton et al., 2006). These high-risk features of CIN suggest that targeting aneuploid cancer cell populations may have therapeutic potential, emphasizing the importance of understanding the cellular processes that initiate and promote tolerance of aneuploidy.

Tumors harbor a wide spectrum of structural and numerical chromosomal alterations (Andor et al., 2015) ranging from diploid or near-diploid tumors to highly aneuploid samples with more complex karyotypes. Notwithstanding that p53 is closely associated with CIN and aneuploidy in CRC, little is known about somatic events that might cooperate with p53 dysfunction in generating or sustaining the accumulation of chromosomal alterations. Our data provide support for *BCL9L* as an aneuploidy tumor-suppressor gene in CRC, the loss of which sustains aneuploidy tolerance, both independently of and in cooperation with p53, through repression of caspase-2. These results are supported by studies in caspase-2 knockout mouse models in which transformed cells develop aneuploidy and become more aggressive (Dorstyn et al., 2012, Puccini et al., 2013).

The mechanisms leading to p53 accumulation in response to chromosomal missegregation events are unclear. DNA damage (Janssen et al., 2011), histone phosphorylation (Hinchcliffe et al., 2016) and reactive oxygen species (Li et al., 2010) have all been proposed as mechanisms of p53 accumulation in CIN cells. Our data reveal that caspase-2 depletion induces tolerance of endogenous chromosome segregation errors and prevents p53 accumulation in response to artificial induction of chromosome segregation errors using an Mps1 inhibitor, reversine, supporting a central role for caspase-2 as an enzyme regulating p53, underpinned by seminal work from other groups (Dorstyn et al., 2012, Oliver et al., 2011, Terry et al., 2015). We found that loss of *BCL9L* prevents cleavage of BID through caspase-2 in *TP53*-null cells and thereby inhibits apoptosis. This p53-independent role for caspase-2 in the suppression of aneuploidy might operate as a fail-safe mechanism to limit CIN in *TP53*-mutant tumors, thereby compromising outgrowth of heterogeneous tumor cells and impairing subsequent tumor adaptation. Conceivably, in *TP53*-WT cells parallel mechanisms of aneuploidy surveillance independent of p53 might reinforce the removal of aneuploid cells in instances where chromosome missegregation events may remain undetected by p53.

Our results support the possibility that caspase-2 can be activated upstream of p53 after chromosome missegregation. This emphasizes the need to elucidate the mechanisms of caspase-2 activation, such as its dependence on phosphorylation (Andersen et al., 2009) or proteotoxic stress, frequently observed in aneuploid cells (Upton et al., 2008). This process might constitute a mechanism of genome instability sensing that can operate independently of p53.

Although evidence supports roles for both BCL9L and BCL9 in the regulation of gene expression that are independent of β-catenin (Cantu et al., 2014), our data suggest that caspase-2 is a target of the β-catenin/TCF4 transcriptional complex. However, it is unclear whether BCL9L regulates a specific subset of genes distinct from its homolog BCL9. Our data support the “just-right” model (Albuquerque et al., 2002) for the modulation of Wnt signaling in tumors to render TCF transcriptional activation sufficient for cancer cell viability, while minimizing transcriptional activation of genes associated with cell death. More specifically, partial inhibition of TCF4 transcriptional activity in tumors with excessive Wnt pathway activation through *BCL9L* dysfunction might reduce caspase-2 expression to levels compatible with cell viability, enhancing tolerance of segregation errors and intratumor heterogeneity.

Consistent with these observations, and notwithstanding the limitations of the xenograft evolutionary experiments due to animal welfare considerations and the resulting short time course of such studies, the xenograft data support the ability of *BCL9L* silencing to propagate intratumor heterogeneity manifested as whole chromosome aneuploidies that are spatially distinct within individual tumors.

Based on the genomic analysis presented here, the characterization of *BCL9L* as an aneuploidy suppressor conforms to a haploinsufficiency model based on results from our analyses of CRC datasets and our functional work. This model has been frequently described for other tumor suppressors such as *PTEN*, *BRCA1*, and *RAD17* (Berger et al., 2011). Such observations may begin to explain why the identification of aneuploidy suppressors has proved evasive, and suggest the need for deeper analysis of genomic datasets focusing on haploinsufficiency as a possible mechanism of tolerance to large-scale karyotypic alterations.

In summary, these data support a role for *BCL9L* as an aneuploidy tolerance gene, conforming to criteria for a significantly mutated driver gene and tumor suppressor in CRC (Giannakis et al., 2016, Tamborero et al., 2013, Vogelstein et al., 2013). Understanding aneuploidy tolerance mechanisms more widely, and the BCL9L/caspase-2/BID axis specifically, may unravel potential vulnerabilities in aneuploid cancers, which could be exploited to limit intercellular heterogeneity, a substrate for selection and tumor evolution.

## Experimental Procedures

### Patient Samples

Tissue collection was approved by an ethics committee (NRS Committee South Central-Oxford B, REC reference 05/Q1605/66), and all individuals included in this study had provided written informed consent for the analysis presented.

### Reversine Survival Screen

Five thousand HCT-116 cells per well were seeded on 96-well plates with siRNA transfection medium and DMSO or 250 nM reversine. Cells were grown for 3 days and cell viability was measured by Cell Titer Blue (Promega). The surviving fraction for each siRNA pool was calculated as the ratio of the fluorescent Cell Titer Blue signal of treated wells between untreated wells (four replicates). Data shown in Figure 1A were normalized to siCtrl2 (siGENOME).

### Reversine Survival Assays

For siRNA short-term survival, cells were plated in transfection medium and 250 nM reversine for 3 days. Cell number was measured by DAPI staining and automated imaging (Acumen), cell viability was measured with Cell Titer Blue (Promega) or alternatively, cells were harvested and analyzed after 1 hr of BrdU incorporation. For colony-forming assays with siRNA, cells were transfected for 3 days and replated in serial dilutions in the presence or absence of reversine. After 5 days of treatment, reversine-containing medium was replaced by drug-free medium and cell colonies grown until the appropriate size was reached.

For long-term colony-forming assays (shRNA), cells were treated for 15 days with 125 nM reversine.

### FISH Analysis

For development of aneuploidy, stable BCL9L knockdown and control cells were treated for 15 days with 125 nM reversine. Reversine-containing medium was replaced by drug-free medium and cells were allowed to recover for 2 weeks. Next, cells were grown on glass slides and centromeric FISH was performed (CEP 1, 2, 8, and 15; Abbot Molecular). Centromeric signals were counted and modal centromeric variation was calculated as the fraction of cells with centromeric signals different from the modal number within the population.

### Animal Procedures

All animal regulated procedures were approved by The Francis Crick Institute BRF Strategic Oversight Committee that incorporates the Animal Welfare and Ethical Review Body and conformed with UK Home Office guidelines and regulations under the Animals (Scientific Procedures) Act 1986 including Amendment Regulations 2012.

### Statistical Methods

For a complete description see Supplemental Experimental Procedures.

## Author Contributions

Conceptualization, C.L.-G., L.S., I.T., and C.S.; Methodology, C.L.-G., L.S., N.McG., E.G., A.J.R., R.B., H.D., and C.S.; Software, Formal Analysis, and Data Curation, C.L.-G., N.McG., N.J.B., S.H., F.F., A.S., M.K., and C.S.; Investigation and Validation, C.L.-G., L.S., S.H., E.G., A.J.R., N.M., S.B., B.P., D.O., M.N., and R.B.; Resources, A.J.R., E.D., H.D., G.S., B.S.-D., and I.T.; Writing – Original Draft, C.L.-G. and C.S.; Writing – Review and Editing and Visualization, C.L.-G., L.S., N.McG., S.H., N.J.B., E.G., I.T., and C.S.; Supervision, C.L.-G., I.T. M.N., H.D., and C.S.; Funding Acquisition, C.S.

## Figures and Tables

**Table 1:** Tumor DNA Computational Purity, DNA Indices, and Centromeric FISH Analysis of a Cohort of 17 MSS Colorectal Adenocarcinomas

Sample	Purity	DNA Index	Chr2			Chr15			Ploidy^c^
			Mode (%MCD)^a^		p Value^b^	Mode (%MCD)^a^		p Value^b^	
			Normal	Tumor		Normal	Tumor		
389	0.42	1.58	2 (41)	1 (60)	0.006	2 (39)	1 (61)	5 × 10^−4^	A
363	0.69	2.23	2 (39)	3 (74)	5 × 10^−4^	2 (32)	2 (65)	5 × 10^−4^	A
391	0.48	1.59	2 (37)	2 (60)	5 × 10^−4^	2 (38)	1 (64)	0.005	A
377	0.40	1.5	2 (42)	3 (60)	5 × 10^−4^	2 (45)	3 (57)	5 × 10^−4^	A
361	0.8	1.27	2 (43)	1 (64)	5 × 10^−4^	2 (30)	2 (54)	0.01	A
379	0.36	1.63	2 (39)	4 (74)	5 × 10^−4^	2 (31)	2 (53)	0.03	A
367	0.21	1.42	2 (36)	3 (56)	5 × 10^−4^	2 (41)	2 (56)	0.1	A
369	0.48	1.61	2 (37)	3 (69)	5 × 10^−4^	2 (38)	3 (55)	5 × 10^−4^	A
365	0.18	1.49	2 (33)	2 (53)	0.002	2 (39)	3 (53)	5 × 10^−4^	A
395	0.42	1	2 (47)	2 (55)	0.12	2 (43)	1 (42)	5 × 10^−4^	A
375	0.35	1	2 (37)	2 (33)	0.14	2 (34)	2 (32)	0.25	D
397	0.49	1	2 (36)	2 (41)	0.18	2 (37)	2 (32)	0.25	D
385	0.56	1	2 (41)	2 (34)	0.22	2 (35)	2 (29)	0.22	D
381	0.63	1	2 (37)	2 (37)	0.4	2 (30)	2 (24)	0.05	D
383	0.47	1	2 (39)	2 (48)	0.5	2 (32)	2 (35)	0.22	D
373	0.65	1	2 (47)	2 (51)	0.7	2 (48)	2 (55)	0.19	D
371	0.35	1	2 (39)	2 (33)	0.12	2 (45)	2 (35)	0.19	D

See also Figures S1 and S2; Table S1.

a Percentage modal centromeric deviation.

b Normal-tumor comparison (two-tailed Fisher's exact test and Monte Carlo simulations).

c A = aneuploid; D = diploid.

**Table 2:** Ploidy and Karyotype Data for the Eight Exome-Sequenced CRC Cell Lines

Cell Line	Age	Dukes Stage	MCN^a^	Ploidy
T84	NR^b^	NR^b^	56	hyperdiploid
SW948	72	C3	67	hypotriploid
SW403	81	C3	68	triploid
SK-CO-1	51	NR^b^	75	hypertriploid
SW1463	65	C1	NR	hypertetraploid
NCI-H508	66	NR^b^	102	hypotriploid
LS123	55	B	63	hypotriploid
HT55	NR^b^	NR^b^	72	hypertriploid

a Modal chromosome number.

b Not reported.

## Accession Numbers

The accession number for whole-exome sequencing and SNP array data reported in this paper is EGA: EGASS00001001907.

## Supplemental Information

Document S1. Supplemental Experimental Procedures, Figures S1–S8, and Tables S1–S4

Movie S1. Two Anaphases at 1 hr 33 min and 3 hr 33 min with Segregation Errors; Daughter Cells Arrest or Die. Related to Figure 2

Movie S2. Anaphase with Chromosomal Bridge at 3 hr 15 min; Daughter Cells Divide and Arrest. Related to Figure 2

Movie S3. Anaphase with Lagging Chromosome at 51 min; Both Daughter Cells Divide. Related to Figure 2

Movie S4. Normal Mitosis at 42 min; Both Daughter Cells Divide. Related to Figure 2

Document S2. Article plus Supplemental Information

## References

[bib1] Albuquerque C., Breukel C., van del Luijt R., Fidalgo P., Lage P., Slors F.J.M., Nobre-Leitao C., Fodde R., Smits R. (2002). The “just-right” signaling model: APC somatic mutations are selected based on a specific level of activation of the β-catenin signaling cascade. Hum. Mol. Genet..

[bib2] Andersen J.L., Johnson C.E., Freel C.D., Parrish A.B., Day J.L., Buchakjian M.R., Nutt L.K., Thompson J.W., Moseley M.A., Kornbluth S. (2009). Restraint of apoptosis during mitosis through interdomain phosphorylation of caspase-2. EMBO J..

[bib3] Andor N., Graham T.A., Jansen M., Xia L.C., Aktipis C.A., Petritsch C., Ji H.P., Maley C.C. (2015). Pan-cancer analysis of the extent and consequences of intratumor heterogeneity. Nat. Med..

[bib4] Bakhoum S.F., Genovese G., Compton D.A. (2009). Deviant kinetochore microtubule dynamics underlie chromosomal instability. Curr. Biol..

[bib5] Bakhoum S.F., Thompson S.L., Manning A.L., Compton D.A. (2009). Genome stability is ensured by temporal control of kinetochore-microtubule dynamics. Nat. Cell Biol..

[bib6] Berger A.H., Knudson A.G., Pandolfi P.P. (2011). A continuum model for tumor suppression. Nature.

[bib7] Bogaert J., Prenen H. (2014). Molecular genetics of colorectal cancer. Ann. Gastroenterol..

[bib8] Boutros P.C., Fraser M., Harding N.J., de Borja R., Trudel D., Lalonde E., Meng A., Hennings-Yeomans P.H., McPherson A., Sabelnykova V.Y. (2015). Spatial genomic heterogeneity within localized, multifocal prostate cancer. Nat. Gen..

[bib9] Burrell R.A., McClelland S.E., Endesfelder D., Groth P., Weller M.C., Shaikh N., Domingo E., Kanu N., Dewhurst S.M., Gronroos E. (2013). Replication stress links structural and numerical cancer chromosomal instability. Nature.

[bib10] Cahill D.P., Lengauer C., Yu J., Riggins G.J., Willson J.K., Markowitz S.D., Kinzler K.W., Vogelstein B. (1998). Mutations of mitotic checkpoint genes in human cancers. Nature.

[bib11] Cancer Genome Atlas Network (2012). Comprehensive molecular characterization of human colon and rectal cancer. Nature.

[bib12] Cantu C., Zimmerli D., Hausmann G., Valenta T., Moor A., Aguet M., Basler K. (2014). Pax6-dependent, but beta-catenin-independent, function of Bcl9 proteins in mouse lens development. Genes Dev..

[bib13] Carter S.L., Eklund A.C., Kohane I.S., Harris L.N., Szallasi Z. (2006). A signature of chromosomal instability inferred from gene expression profiles predicts clinical outcome in multiple human cancers. Nat. Gen..

[bib14] Chan K.L., North P.S., Hickson I.D. (2007). BLM is required for faithful chromosome segregation and its localization defines a class of ultrafine anaphase bridges. EMBO J..

[bib15] Chin S.F., Teschendorff A.E., Marioni J.C., Wang Y., Barbosa-Morais N.L., Thorne N.P., Costa J.L., Pinder S.E., van de Wiel M.A., Green A.R. (2007). High-resolution aCGH and expression profiling identifies a novel genomic subtype of ER negative breast cancer. Genome Biol..

[bib16] Cianchi F., Balzi M., Becciolini A., Giache V., Messerini L., Palomba A., Tisti E., Faraoni P., Chellini F., Pucciani F. (1999). Correlation between DNA content and p53 deletion in colorectal cancer. Eur. J. Sur..

[bib17] Crasta K., Ganem N.J., Dagher R., Lantermann A.B., Ivanova E.V., Pan Y., Nezi L., Protopopov A., Chowdhury D., Pellman D. (2012). DNA breaks and chromosome pulverization from errors in mitosis. Nature.

[bib18] Danielsen H.E., Pradhan M., Novelli M. (2016). Revisiting Tumor aneuploidy - the place of ploidy assessment in the molecular era. Nat. Rev. Clin. Oncol..

[bib19] de la Roche M., Worm J., Bienz M. (2008). The function of BCL9 in Wnt/beta-catenin signaling and colorectal cancer cells. BMC Cancer.

[bib20] Desmedt C., Fumagalli D., Pietri E., Zoppoli G., Brown D., Nik-Zainal S., Gundem G., Rothe F., Majjaj S., Garuti A. (2015). Uncovering the genomic heterogeneity of multifocal breast cancer. J. Pathol..

[bib21] Dewhurst S.M., McGranahan N., Burrell R.A., Rowan A.J., Gronroos E., Endesfelder D., Joshi T., Mouradov D., Gibbs P., Ward R.L. (2014). Tolerance of whole-genome doubling propagates chromosomal instability and accelerates cancer genome evolution. Cancer Discov..

[bib22] Ding L., Ley T.J., Larson D.E., Miller C.A., Koboldt D.C., Welch J.S., Ritchey J.K., Young M.A., Lamprecht T., McLellan M.D. (2012). Clonal evolution in relapsed acute myeloid leukaemia revealed by whole-genome sequencing. Nature.

[bib23] Dorstyn L., Puccini J., Wilson C.H., Shalini S., Nicola M., Moore S., Kumar S. (2012). Caspase-2 deficiency promotes aberrant DNA-damage response and genetic instability. Cell Death Differ..

[bib24] Gaasenbeek M., Howarth K., Rowan A.J., Gorman P.A., Jones A., Chaplin T., Liu Y., Bicknell D., Davison E.J., Fiegler H. (2006). Combined array-comparative genomic hybridization and single-nucleotide polymorphism-loss of heterozygosity analysis reveals complex changes and multiple forms of chromosomal instability in colorectal cancers. Cancer Res..

[bib25] Gascoigne K.E., Taylor S.S. (2008). Cancer cells display profound intra-and interline variation following exposure to antimitotic drugs. Cancer Cell.

[bib26] Giannakis M., Mu X.J., Shukla S.A., Qian Z.R., Cohen O., Nishihara R., Bahl S., Cao Y., Amin-Mansour A., Yamauchi M. (2016). Genomic correlates of immune-cell infiltrates in colorectal carcinoma. Cell Rep..

[bib27] Grim J.E., Knoblaugh S.E., Guthrie K.A., Hagar A., Swanger J., Hespelt J., Delrow J.J., Small T., Grady W.M., Nakayama K.I., Clurman B.E. (2012). Fbw7 and p53 cooperatively suppress advanced and chromosomally unstable intestinal cancer. Mol. Cell. Biol..

[bib28] Guo Y., Srinivasula S.M., Druilhe A., Fernandes-Alnemri T., Alnemri E.S. (2002). Caspase-2 induces apoptosis by releasing proapoptotic proteins from mitochondria. J. Biol. Chem..

[bib29] Hinchcliffe E.H., Day C.A., Karanjeet K.B., Fadness S., Langfald A., Vaughan K.T., Dong Z. (2016). Chromosome missegregation during anaphase triggers p53 cell cycle arrest through histone H3.3 Ser31 phosphorylation. Nat. Cell Biol..

[bib30] Iwanaga Y., Chi Y.H., Miyazato A., Sheleg S., Haller K., Peloponese J.M., Li Y., Ward J.M., Benezra R., Jeang K.T. (2007). Heterozygous deletion of mitotic arrest-deficient protein 1 (MAD1) increases the incidence of tumors in mice. Cancer Res..

[bib31] Janssen A., van der Burg M., Szuhai K., Kops G.J., Medema R.H. (2011). Chromosome segregation errors as a cause of DNA damage and structural chromosome aberrations. Science.

[bib32] Jemaa M., Galluzzi L., Keep O., Boileve A., Lissa D., Senovilla L., Harper F., Pierron G., Berardinelli F., Antoccia A. (2012). Preferential killing of p53-deficient cancer cells by reversine. Cell Cycle.

[bib33] Karni-Schmidt O., Lokshin M., Prives C. (2016). The roles for MDM2 and MDMX in cancer. Annu. Rev. Pathol..

[bib34] Kawabata T., Luebben S.W., Yamaguchi S., Ilves I., Matise I., Buske T., Botchan M.R., Shima N. (2011). Stalled fork rescue via dormant replication origins in unchallenged S phase promotes proper chromosome segregation and tumor suppression. Mol. Cell.

[bib35] Korsmeyer S.J., Wei M.C., Saito M., Weiler S., Oh K.J., Schlesinger P.H. (2000). Pro-apoptotic cascade activates BID, which oligomerizes BAK or BAX into pores that result in the release of cytochrome c. Cell Death Differ..

[bib36] Kronenwett U., Huwendiek S., Ostring C., Portwood N., Roblick U.J., Pawitan Y., Alaiya A., Sennerstam R., Zetterberg A., Auer G. (2004). Improved grading of breast adenocarcinomas based on genomic instability. Cancer Res..

[bib37] Kuznetsova A.Y., Seget K., Moeller G.K., de Pagter M.S., de Roos J.A., Durrbaum M., Kuffer C., Muller S., Zaman G.J., Kloosterman W.P. (2015). Chromosomal instability, tolerance of mitotic errors and multidrug resistance are promoted by tetraploidization in human cells. Cell Cycle.

[bib38] Landau D.A., Carter S.L., Stojanov P., McKenna A., Stevenson K., Lawrence M.S., Sougnez C., Stewart C., Sivachenko A., Wang L. (2013). Evolution and impact of subclonal mutations in chronic lymphocytic leukemia. Cell.

[bib39] Lee A.J., Endesfelder D., Rowan A.J., Walther A., Birkbak N.J., Futreal P.A., Downward J., Szallasi Z., Tomlinson I.P., Howell M. (2011). Chromosomal instability confers intrinsic multidrug resistance. Cancer Res..

[bib40] Lengauer C., Kinzler K.W., Vogelstein B. (1997). Genetic instability in colorectal cancer. Nature.

[bib41] Lengauer C., Kinzler K.W., Vogelstein B. (1998). Genetic instabilities in human cancers. Nature.

[bib42] Li M., Fang X., Baker D.J., Guo L., Gao X., Wei Z., Han S., van Deursen J.M., Zhang P. (2010). The ATM-p53 pathway suppresses aneuploidy-induced tumorigenesis. Proc. Natl. Acad. Sci. USA.

[bib43] Mouradov D., Domingo E., Gibbs P., Jorissen R.N., Li S., Soo P.Y., Lipton L., Desai J., Danielsen H.E., Oukrif D. (2013). Survival in stage II/III colorectal cancer is independently predicted by chromosomal and microsatellite instability, but not by specific driver mutations. Am. J. Gastroenterol..

[bib44] Negrini S., Gorgoulis V.G., Halazonetis T.D. (2010). Genomic instability–an evolving hallmark of cancer. Nat. Rev. Mol. Cell Biol..

[bib45] Oliver T.G., Meylan E., Chang G.P., Xue W., Burke J.R., Humpton T.J., Hubbard D., Bhutkar A., Jacks T. (2011). Caspase-2-mediated cleavage of Mdm2 creates a p53-induced positive feedback loop. Mol. Cell.

[bib46] Orsetti B., Selves J., Bascoul-Mollevi C., Lasorsa L., Gordien K., Bibeau F., Massemin B., Paraf F., Soubeyran I., Hostein I. (2014). Impact of chromosomal instability on colorectal cancer progression and outcome. BMC Cancer.

[bib47] Pampalona J., Soler D., Genesca A., Tusell L. (2010). Whole chromosome loss is promoted by telomere dysfunction in primary cells. Genes Chromosomes Cancer.

[bib48] Puccini J., Shalini S., Voss A.K., Gatei M., Wilson C.H., Hiwase D.K., Lavin M.F., Dorstyn L., Kumar S. (2013). Loss of caspase-2 augments lymphomagenesis and enhances genomic instability in Atm-deficient mice. Proc. Natl. Acad. Sci. USA.

[bib49] Rosenbloom K.R., Dreszer T.R., Long J.C., Malladi V.S., Sloan C.A., Raney B.J., Cline M.S., Karolchik D., Barber G.P., Clawson H. (2012). ENCODE whole-genome data in the UCSC Genome Browser: update 2012. Nucleic Acids Res..

[bib50] Rowan A., Halford S., Gaasenbeek M., Kemp Z., Sieber O., Volikos E., Douglas E., Fiegler H., Carter N., Talbot I. (2005). Refining molecular analysis in the pathways of colorectal carcinogenesis. Clin. Gastroenterol. Hepatol..

[bib51] Santaguida S., Tighe A., D'Alise A.M., Taylor S.S., Musacchio A. (2010). Dissecting the role of MPS1 in chromosome biorientation and the spindle checkpoint through the small molecule inhibitor reversine. J. Cell Biol..

[bib52] Sotillo R., Hernando E., Diaz-Rodriguez E., Teruya-Feldstein J., Cordon-Cardo C., Lowe S.W., Benezra R. (2007). Mad2 overexpression promotes aneuploidy and tumorigenesis in mice. Cancer Cell.

[bib53] Stingele S., Stoehr G., Storchova Z. (2013). Activation of autophagy in cells with abnormal karyotype. Autophagy.

[bib54] Swanton C., Tomlinson I., Downward J. (2006). Chromosomal instability, colorectal cancer and taxane resistance. Cell Cycle.

[bib55] Szerlip N.J., Pedraza A., Chakravarty D., Azim M., McGuire J., Fang Y., Ozawa T., Holland E.C., Huse J.T., Jhanwar S. (2012). Intratumoral heterogeneity of receptor tyrosine kinases EGFR and PDGFRA amplification in glioblastoma defines subpopulations with distinct growth factor response. Proc. Natl. Acad. Sci. USA.

[bib56] Tamborero D., Gonzalez-Perez A., Perez-Llamas C., Deu-Pons J., Kandoth C., Reimand J., Lawrence M.S., Getz G., Bader G.D., Ding L., Lopez-Bigas N. (2013). Comprehensive identification of mutational cancer driver genes across 12 tumor types. Sci. Rep..

[bib57] Tang R., Changchien C.R., Wu M.C., Fan C.W., Liu K.W., Chen J.S., Chien H.T., Hsieh L.L. (2004). Colorectal cancer without high microsatellite instability and chromosomal instability–an alternative genetic pathway to human colorectal cancer. Carcinogenesis.

[bib58] Terry M.R., Arya R., Mukhopadhyay A., Berrett K.C., Clair P.M., Witt B., Salama M.E., Bhutkar A., Oliver T.G. (2015). Caspase-2 impacts lung tumorigenesis and chemotherapy response in vivo. Cell Death Differ..

[bib59] Thompson S.L., Compton D.A. (2008). Examining the link between chromosomal instability and aneuploidy in human cells. J. Cell Biol..

[bib60] Thompson S.L., Compton D.A. (2010). Proliferation of aneuploid human cells is limited by a p53-dependent mechanism. J. Cell Biol..

[bib61] Torres E.M., Dephoure N., Panneerselvam A., Tucker C.M., Whittaker C.A., Gygi S.P., Dunham M.J., Amon A. (2010). Identification of aneuploidy-tolerating mutations. Cell.

[bib62] Upton J.P., Austgen K., Nishino M., Coakley K.M., Hagen A., Han D., Papa F.R., Oakes S.A. (2008). Caspase-2 cleavage of BID is a critical apoptotic signal downstream of endoplasmic reticulum stress. Mol. Cell. Biol..

[bib63] Vogelstein B., Papadopoulos N., Velculescu V.E., Zhou S., Diaz L.A., Kinzler K.W. (2013). Cancer genome landscapes. Science.

[bib64] Yates L.R., Gerstung M., Knappskog S., Desmedt C., Gundem G., Van Loo P., Aas T., Alexandrov L.B., Larsimont D., Davies H. (2015). Subclonal diversification of primary breast cancer revealed by multiregion sequencing. Nat. Med..

[bib65] Yoo J.W., Seo K.W., Jang S.J., Oh Y.M., Shim T.S., Kim W.S., Lee D.S., Lee S.D., Choi C.M. (2010). The relationship between the presence of chromosomal instability and prognosis of squamous cell carcinoma of the lung: fluorescence in situ hybridization analysis of paraffin-embedded tissue from 47 Korean patients. J. Korean Med. Sci..

