# Supplementary material for: BCL9L Dysfunction Impairs Caspase-2 Expression Permitting Aneuploidy Tolerance in Colorectal Cancer
Source: Cancer Cell. 2017 Jan 9;31(1):79–93. doi: 10.1016/j.ccell.2016.11.001 (PMC5225404; doi:10.1016/j.ccell.2016.11.001)
Supplement: Document S1. Supplemental Experimental Procedures, Figures S1–S8, and Tables S1–S4 [file mmc1.pdf]

Cancer Cell, Volume *31*

Supplemental Information

BCL9L Dysfunction Impairs Caspase-2 Expression Permitting Aneuploidy Tolerance in Colorectal Cancer

Carlos López-García, Laurent Sansregret, Enric Domingo, Nicholas McGranahan, Sebastijan Hobor, Nicolai Juul Birkbak, Stuart Horswell, Eva Grönroos, Francesco Favero, Andrew J. Rowan, Nicholas Matthews, Sharmin Begum, Benjamin Phillimore, Rebecca Burrell, Dahmane Oukrif, Bradley Spencer-Dene, Michal Kovac, Gordon Stamp, Aengus Stewart, Havard Danielsen, Marco Novelli, Ian Tomlinson, and Charles Swanton

SUPPLEMENTAL DATA

Table S1: Related to Table 1. Clinical features of the whole-exome sequenced cohort of 17 MSS colorectal adenocarcinomas.

Tumor	Sex	Age	Differentiation	Tumor Classification	Dukes stage	TNM	RLyV	Liver mets
**361**	M	66	Well/moderate	Adenocarcinoma	C1	pT3 N1 Mx	R0 Ly1 V1	Yes
**363**	M	52	Moderate	Adenocarcinoma	N.R.[(a)]	pT4 N0 Mx	R0 Ly0 V0	Yes
**365**	F	66	Moderate	Adenocarcinoma	A	pT2 N0 Mx	R0 Ly0 V0	Yes
**367**	M	76	Moderate	Adenocarcinoma	C1	pT3 N1 Mx	R0 Ly0 V0	Yes
**369**	M	48	Moderate	Adenocarcinoma	C1	pT3,N1,M1	R0 Ly0 V0	Yes
**371**	F	79	Moderate	Mucinous adenocarcinoma	B	ypT4 N0 Mx	R0 Ly0 V0	No
**373**	M	29	Moderate	Mucinous adenocarcinoma	C1	pT3 N2 M1	Ly1 V1	Yes
**375**	F	70	Well/moderate	Adenocarcinoma	B	pT3 N0 Mx	R0 Ly0 V0	No
**377**	M	61	Moderate	Adenocarcinoma	A	pT2 N0 Mx	R0 Ly0 V1	No
**379**	M	68	Moderate	Mucinous adenocarcinoma	A	pT2 N0 Mx	R0 Ly0 V0	No
**381**	F	64	Moderate	Adenocarcinoma	A	pT2 N0 Mx	R0 Ly0 V0	No
**383**	F	74	Moderate	Adenocarcinoma	A	pT2 N0 Mx	R0 Ly0 V0	No
**385**	M	70	Well/moderate	Adenocarcinoma	C1	T4 N2 M0	R0 Ly0 V0	No
**389**	M	58	Well/moderate	Adenocarcinoma	B	pT3 N0 Mx	R0Ly0V0	No
**391**	M	70	Well/moderate	Adenocarcinoma	A	pT2 N0 Mx	R0Ly0V0	No
**395**	M	59	Poor	Adenocarcinoma	B	pT3 N0 Mx	R0 Ly0 V1	No
**397**	M	74	Moderate	Adenocarcinoma	B	pT4 N0 Mx	R0 Ly0 V0	No

([a]) Not reported.

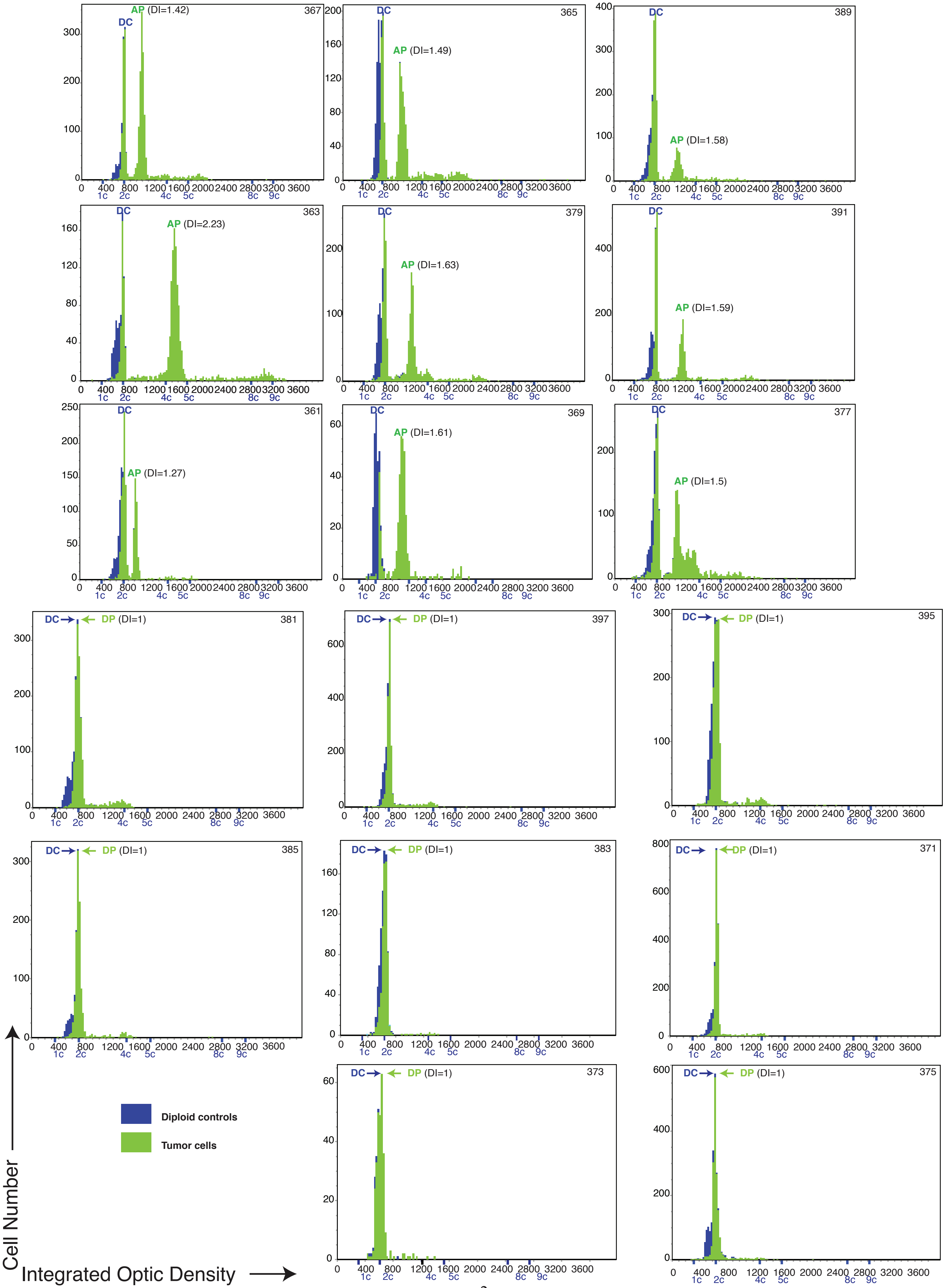

AP (DI=1.42)
DC
367
DC
AP (DI=1.49)
365
DC
AP (DI=1.58)
389
DC
AP (DI=2.23)
363
DC
AP (DI=1.63)
379
DC
AP (DI=1.59)
391
DC
AP (DI=1.27)
361
DC
AP (DI=1.61)
369
DC
AP (DI=1.5)
377
DC → ← DP (DI=1)
381
DC → ← DP (DI=1)
397
DC → ← DP (DI=1)
395
DC → ← DP (DI=1)
385
DC → ← DP (DI=1)
383
DC → ← DP (DI=1)
371
DC → ← DP (DI=1)
373
DC → ← DP (DI=1)
375
0 400 800 1200 1600 2000 2400 2800 3200 3600
1c 2c 4c 5c 8c 9c
Diploid controls
Tumor cells
Cell Number
Integrated Optic Density

Figure S1. Related to Table 1. Ploidy analysis of a cohort of 17 MSS colorectal adenocarcinomas by DNA image cytometry (see Supplemental Experimental Procedures). Histograms show DNA indices (DI) associated to aneuploid and diploid peaks calculated using adjacent fibroblasts, endothelial and infiltrated immune cells as diploid controls. Blue histograms represent diploid controls (fibroblasts, endothelial and infiltrated immune cells); green histograms represent tumor cells. The position of the modal DNA content of the G_1/G_0 diploid controls has been annotated as DC and marks the 2c position. The position of the modal DNA content of the aneuploid tumor cell population has been annotated as AP and the diploid tumor cell population as DP. 1c, 2c, 4c, 5c, 8c and 9c annotation on the horizontal axis mark the theoretical position of the 1N, 2N, 4N, 5N , 8N and 9N populations relative to the diploid controls.

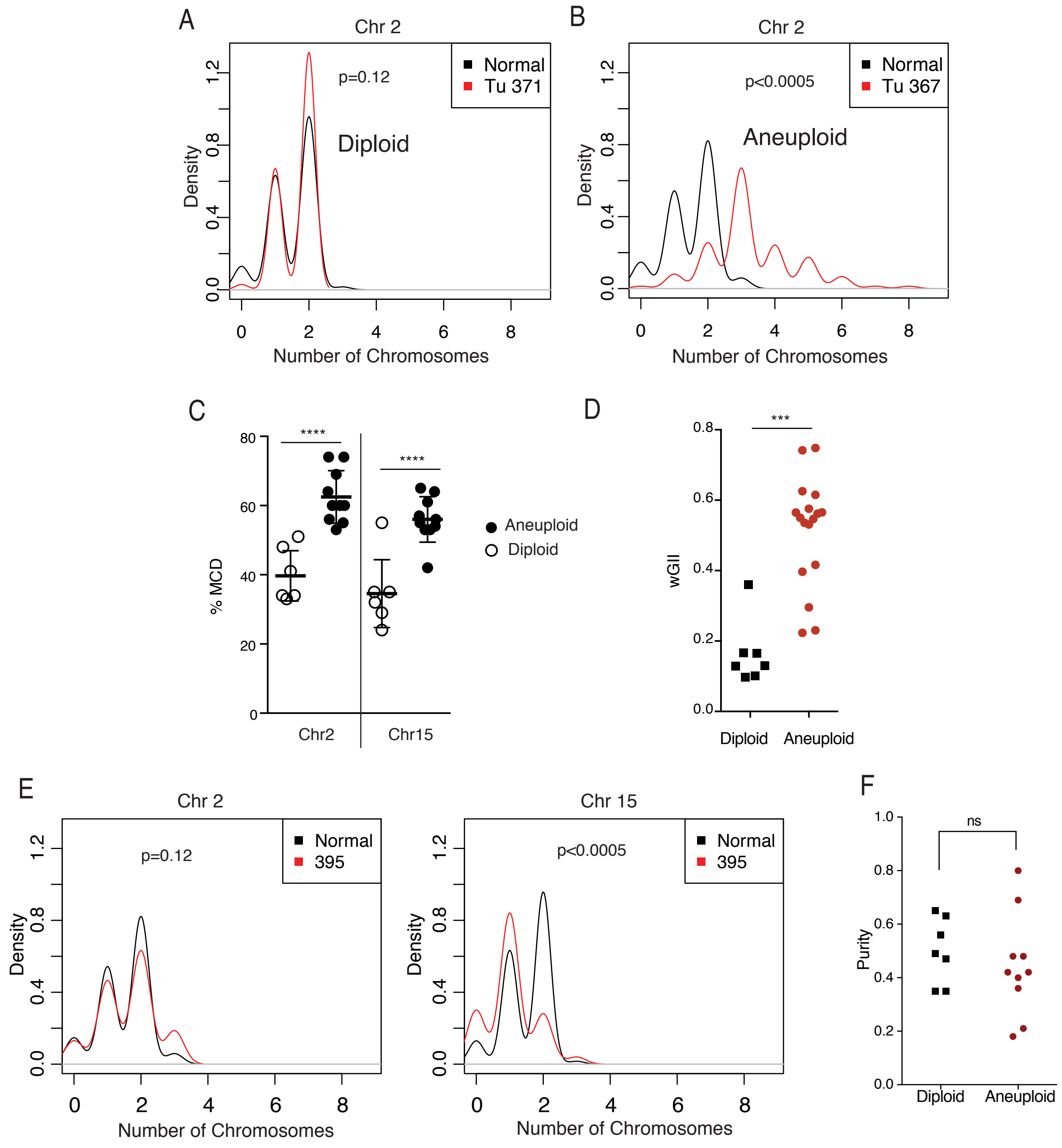

Figure S2. Related to Table 1. (**A -B**) Examples of analysis of chromosome 2 centromeric FISH on a diploid **(A)** and an aneuploid **(B)** colorectal adenocarcinoma. Normal adjacent tissue was used as diploid control (p values calculated by two-tailed Fisher's exact test and Monte Carlo simulations). **(C)** Comparison of the modal centromeric deviation (%MCD) from aneuploid and diploid CRC tumours (chr2 and chr15, p values calculated by unpaired Student's t-test). (**D)** Comparison of the weighted genome instability index (wGII) of aneuploid and diploid CRC tumours (p values calculated by unpaired Student's t-test). (**E)** Centromeric FISH analysis (Chr 2 and 15) of sample 395. (**F)** Difference in tumour cell purity between diploid and aneuploid CRC tumours (p value calculated by unpaired Student's t-test).

Error bars denote SD. ns=not significant, ***p<0.001, ****p<0.0001.

Table S2: Related to Figure 1. Whole-exome sequencing depth and number of variant calls before manual curation in a cohort of 17 MSS colorectal adenocarcinomas and 8 aneuploid cell lines.

Sample (N=Normal)	Median depth (reads)	Non-synonymous calls
**T84**	49	342
**SW480**	43	441
**SW403**	53	598
**SK-CO-1**	45	284
**SW1463**	52	617
**NCI-508**	49	392
**LS123**	54	555
**HT55**	45	1452
**361**	52	38
**362 (N)**	49	
**363**	49	123
**364 (N)**	51	
**365**	37	33
**366 (N)**	45	
**367**	49	51
**368 (N)**	48	
**369**	46	115
**370 (N)**	42	
**371**	43	123
**372 (N)**	48	
**373**	39	100
**374 (N)**	37	
**375**	41	94
**376 (N)**	43	
**377**	54	81
**378 (N)**	51	
**379**	50	84
**380 (N)**	49	
**381**	46	69
**382 (N)**	46	
**383**	57	113
**384 (N)**	47	
**385**	54	102
**386 (N)**	52	
**389**	55	41
**390 (N)**	44	
**391**	63	57
**392 (N)**	53	
**395**	55	65
**396 (N)**	57	
**397**	41	59
**398 (N)**	34	

Table S3. Related to Figure 1. Details of the somatic mutations shown in Figure 1A (note that 371, 397 samples are not shown since they do not display any mutation in genes shown in Figure 1A)

Sample	Chromosome	Position	Germline	Tumour	*Gene Symbol*	Mutation Type
361	chr17	7577157	T	A	*TP53*	Exon 5 -2
	chr3	74347140	C	A	*CNTN3*	S790I
	chrX	32360256	G	C	*DMD*	S1961R
	chr5	112173917	C	T	*APC*	R876X
	chr5	112175216	G	T	*APC*	E1309X
363	chr17	7577547	C	A	*TP53*	G245V
	chr11	118773944	C	T	*BCL9L*	Exon 5 +1
	chr2	168102142	C	T	*XIRP2*	Q1414X
	chr10	26241152	T	C	*MYO3A*	V38A
	chr2	242179190	G	A	*HDLBP*	R813C
	chr18	10485711	G	A	*APCDD1*	A343T
	chr9	125838936	G	C	*RABGAP1*	L701F
	chr3	74350696	G	A	*CNTN3*	P650S
	chr5	112176030	T	A	*APC*	A1580N
365	chr17	7673803	C	T	*TP53*	R273C
367	chr17	7577094	G	A	*TP53*	R282W
	chrX	32632524	A	C	*DMD*	L460V
369	chr3	64619218	C	T	*ADAMTS9*	R702Q
	chrX	117773430	G	A	*DOCK11*	G1345E
	chr16	72991587	G	A	*ZFHX3*	R820C
	chr2	128877943	G	T	*UGGT1*	D272Y
	chr12	25398284	C	A	*KRAS*	G12V
	chr5	112174631	C	T	*APC*	R1114X
373	chr5	112164586	C	T	*APC*	R554X
375	chr5	112175706	T	-	*APC*	frameshift
377	chr17	7578176	G	T	*TP53*	Exon 3 +1
	chr10	26377153	G	T	*MYO3A*	E461X
	chr13	101881748	G	A	*NALCN*	P541L
	chr13	77862394	T	C	*MYCBP2*	I128V
	chr2	171258135	A	G	*MYO3B*	Y688C
	chr13	25353803	A	T	*RNF17*	Exon 5 -2
	chr16	72984723	G	A	*ZFHX3*	T954M
	chr1	202702954	C	T	*JARID1B*	A1162T
	chr12	25398281	C	T	*KRAS*	G13D
	chr5	112174268	A	T	*APC*	K993X
	chr5	112116592	C	T	*APC*	R213X
379	chr11	118772315	G	A	*BCL9L*	Q713X
	chr20	34021715	A	G	*GDF5*	C500R
	chr13	101763474	A	G	*NALCN*	Exon 20 +2
	chr15	84611752	G	A	*ADAMTSL3*	C803Y
	chr6	51923374	C	T	*PKHD1*	G420D
	chr12	25398281	C	T	*KRAS*	G13D
381	chr12	25398284	C	A	*KRAS*	G12V
	chr5	112174182	-	A	*APC*	frameshift
383	chr5	112128191	C	T	*APC*	R232X
	chr12	25398284	C	T	*KRAS*	G12D
385	chr4	153249384	C	A	*FBXW7*	R347L
	chr12	25398281	C	T	*KRAS*	G13D
	chr5	112176027	T	-	*APC*	frameshift
	chr5	112173349	-	C	*APC*	frameshift
389	chr17	7577094	G	A	*TP53*	H282Y
	chr6	10907840	G	T	*SYCP2L*	E248X
	chr12	25398284	C	T	*KRAS*	G12D

391	chr17	7571720	G	A	*TP53*	Exon 8 +1
	chr1	26366306	G	A	*SLC30A2*	T312M
	chr5	112175212	AAAAG	-	*APC*	frameshift
395	chr17	7578406	C	T	*TP53*	R175H
	chr5	13894933	G	T	*DNAH5*	Exon 16 +2
	chr5	112175212	AAAAG	-	*APC*	frameshift
SW403	chr17	7579536	C	A	*TP53*	E51X
	chr2	168106261	G	T	*XIRP2*	E2787X
	chr3	64672354	G	A	*ADAMTS9*	Q136X
	chr10	26459447	A	T	*MYO3A*	K1126M
	chr1	204425158	G	T	*PIK3C2B*	T590N
	chr1	202729562	C	A	*JARID1B*	C353F
	chr6	51613167	C	T	*PKHD1*	V3083M
	chr2	128910379	G	T	*UGCGL1*	D656Y
	chrX	153587463	C	T	*FLNA*	G1455R
	chr20	50048607	C	A	*NFATC2*	D907Y
	chr5	112174884	C	T	*APC*	S1198F
	chr5	112175124	C	A	*APC*	S1278X
HT55	chr17	7578211	C	A	*TP53*	R213L
	chr2	242187700	T	A	*HDLBP*	K526X
	chr13	101844385	C	A	*NALCN*	Q549H
	chr1	204426163	A	C	*PIK3C2B*	Exon 11 +2
	chr2	171399474	A	T	*MYO3B*	K1212X
	chrX	117805037	G	T	*DOCK11*	E1710X
	chr13	101844385	C	A	*NALCN*	Q549H
	chr4	122765089	T	A	*BBS7*	D433V
	chrX	91132857	A	T	*PCDH11X*	I540F
	chrX	32404536	A	T	*DMD*	V1522E
	chrX	153581290	A	T	*FLNA*	Y2069N
	chr4	153303466	T	A	*FBXW7*	T8S
	chr5	112174682	C	T	*APC*	Q1131X
	chr5	112175198	C	T	*APC*	Q130X3
	chr5	112175680	A	T	*APC*	R1463S
NCI-H508	chr17	7577120	C	T	*TP53*	R273H
	chr10	26417463	A	G	*MYO3A*	E753G
	chr20	34022560	C	A	*GDF5*	R218M
SK-CO-1	chr3	64641310	G	T	*ADAMTS9*	Q371K
	chr2	242195813	T	A	*HDLBP*	D220V
	chr18	10485730	G	A	*APCDD1*	R349H
	chr18	5397160	A	G	*EPB41L3*	V913A
	chr9	125752338	A	C	*RABGAP1*	S185R
	chr15	84651057	G	A	*ADAMTSL3*	E893K
	chr4	122782666	T	C	*BBS7*	K112E
	chrX	91134258	G	A	*PCDH11X*	V1007I
	chr12	49087306	G	A	*CCNT1*	S564F
	chr12	25398284	C	A	*KRAS*	G12V
	chr5	112838859	T	-	*APC*	frameshift
T84	chr20	34022340	C	T	*GDF5*	W291X
	chr1	204423833	G	A	*PIK3C2B*	S677F
	chr16	72821983	C	G	*ZFHX3*	A3398P
	chr21	41550978	C	A	*DSCAM*	Q941H
	chr12	49089589	T	A	*CCNT1*	I244F
	chr12	25398281	C	T	*KRAS*	G13D
	chr5	112840058	T	-	*APC*	frameshift
LS123	chr17	7578406	C	T	*TP53*	R175H
	chr2	168104014	G	A	*XIRP2*	A2038T
	chr3	64617175	C	T	*ADAMTS9*	R782Q
	chr3	74316452	C	G	*CNTN3*	A928P

	chr21	41416190	C	T	*DSCAM*	R1733Q
	chr12	25398285	C	T	*KRAS*	G12S
	chr5	112170777	C	T	*APC*	Q625X
	chr5	112175639	C	T	*APC*	R1450X
SW948	chr6	10898308	C	T	*SYCP2L*	S134L
	chr13	77670484	T	G	*MYCBP2*	K3268T
	chr13	77765922	T	C	*MYCBP2*	E1283G
	chr1	26371527	A	T	*SLC30A2*	S78T
	chr20	50049005	G	A	*NFATC2*	P774L
	chr12	25380276	T	A	*KRAS*	Q61L
	chr5	112174631	C	T	*APC*	R1114X
	chr5	112175576	C	T	*APC*	Q1429X
SW1463	chr17	7577538	C	T	*TP53*	R248Q
	chr11	118772306	G	A	*BCL9L*	R716X
	chr2	168100943	T	A	*XIRP2*	V1014E
	chr5	13830149	G	A	*DNAH5*	L2079F
	chr3	74349047	C	A	*CNTN3*	S713I
	chrx	91132792	G	A	*PCDH11X*	R518H
	chr4	153247366	C	T	*FBXW7*	R361Q
	chr12	25398285	C	A	*KRAS*	G12C
	chr5	112176756	TG	-	*APC*	frameshift

Table S4: Related to Figure 1. Missense *BCL9L* mutations and Polyphen scores of predicted functional impact in 4 MSS CRC cohorts (TCGA, Giannakis et al., 2016 and 2 validation cohorts of 30 and 26 samples).

***BCL9L* mutation**	**Polyphen score**
E676G	Probably damaging
S700N	Benign
P842S	Probably damaging
Q1381R	Probably damaging
P1077R	Probably damaging
P1197A	Benign
A578V	Benign
L1064P	Benign
R409W (3)	Probably damaging
T1100I	Probably damaging
P356L	Benign
R581W	Benign
S410F	Probably damaging
V828	Benign
A1009C	Probably damaging

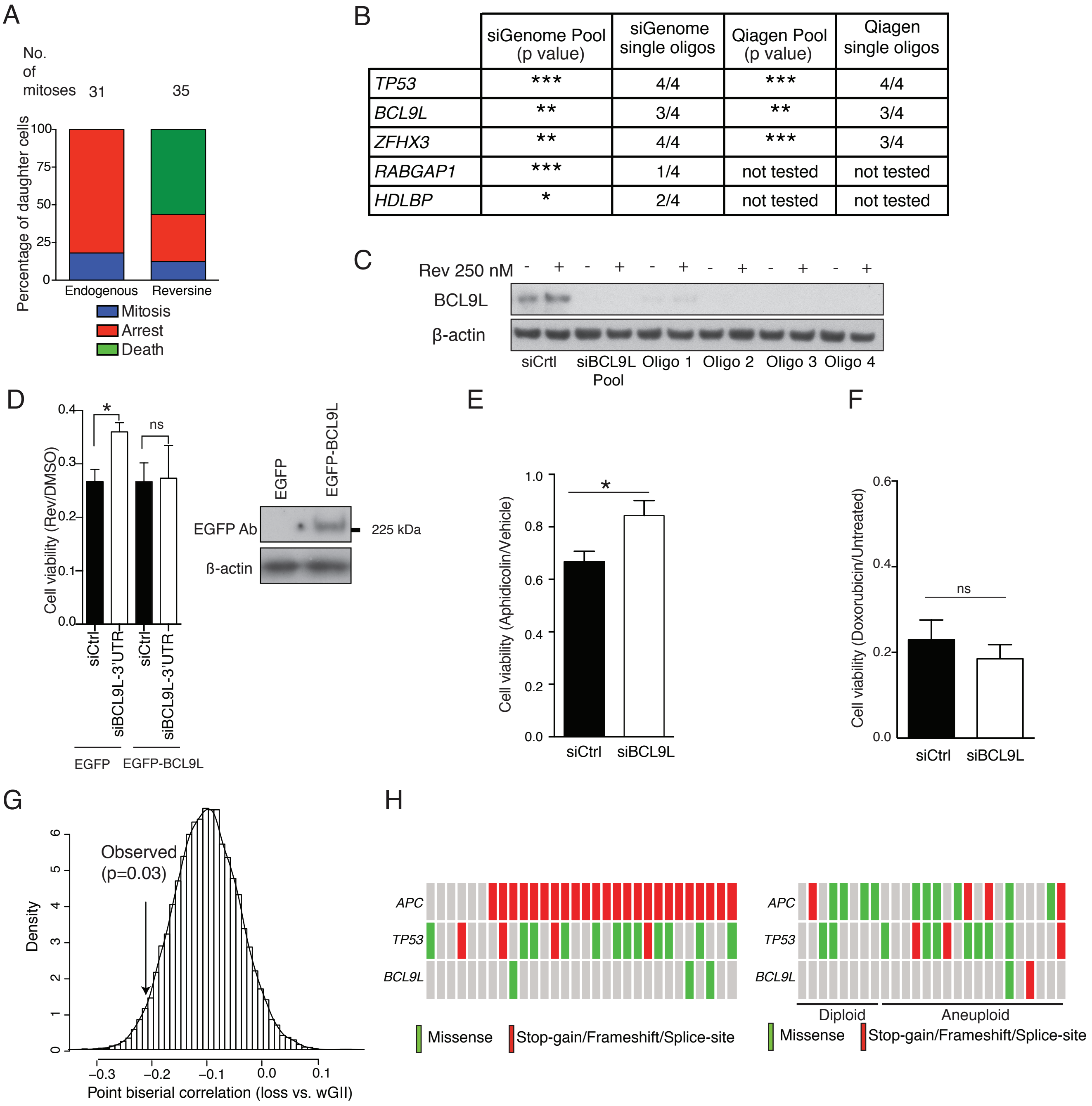

	siGenome Pool (p value)	siGenome single oligos	Qiagen Pool (p value)	Qiagen single oligos
TP53	***	4/4	***	4/4
BCL9L	**	3/4	**	3/4
ZFHX3	**	4/4	***	3/4
RABGAP1	***	1/4	not tested	not tested
HDLBP	*	2/4	not tested	not tested

Figure S3. Related to Figure 1. **(A)** Fate of daughter cells after endogenous or reversine-induced segregation error studied by live-cell imaging of HCT-116 cells expressing H2B-RFP. **(B)** Summary of the effect of TP53, BCL9L, RABGAP1, HDLBP and ZFHX3 knock-down on cell survival upon 250 nM with pools of 4 and indivudual siRNA duplexes (n=3, p value calculated by paired Student's t-test). **(C)** Western blot analysis of the BCL9L knock-down efficiency following transfection of an siRNA pool or transfection of individual duplexes (Qiagen). **(D)** Viability of HCT-116 cells co-transfecting a BCL9L-EGFP construct and a single siRNA duplex targeting the 3'-UTR region of *BCL9L* mRNA as indicated in the presence and absence of 250 nM reversine. (siRNA duplex shown in Supplemental Experimental Procedures, 72 hr, n=3, p value calculated by Student's t-test). **(E)** Effect of BCL9L depletion on survival upon aphidicolin treatment (200 nM, 4 days, n=3, p value calculated by paired Student's t-test) **(F)** Effect of BCL9L depletion on survival upon doxorubicin treatment (100 nM, 3 days, n=3, p value calculated by paired Student's t-test). **(G)** Computational permutation analysis showing a correlation between wGII and *BCL9L* copy-number loss in MSS CRC (TCGA, see Supplemental Experimental Procedures). **(H)** Mutational status of *APC*, *TP53* and *BCL9L* determined using Ion Torrent targeted sequencing in a cohort of 30 MSS CRC tumours (left) and a cohort of 26 MSS CRC tumours (right) of known ploidy (analyzed by DNA image cytometry).

Error bars denote SD. ns=not significant, *p<0.05, **p<0.001***p<0.001, ****p<0.0001.

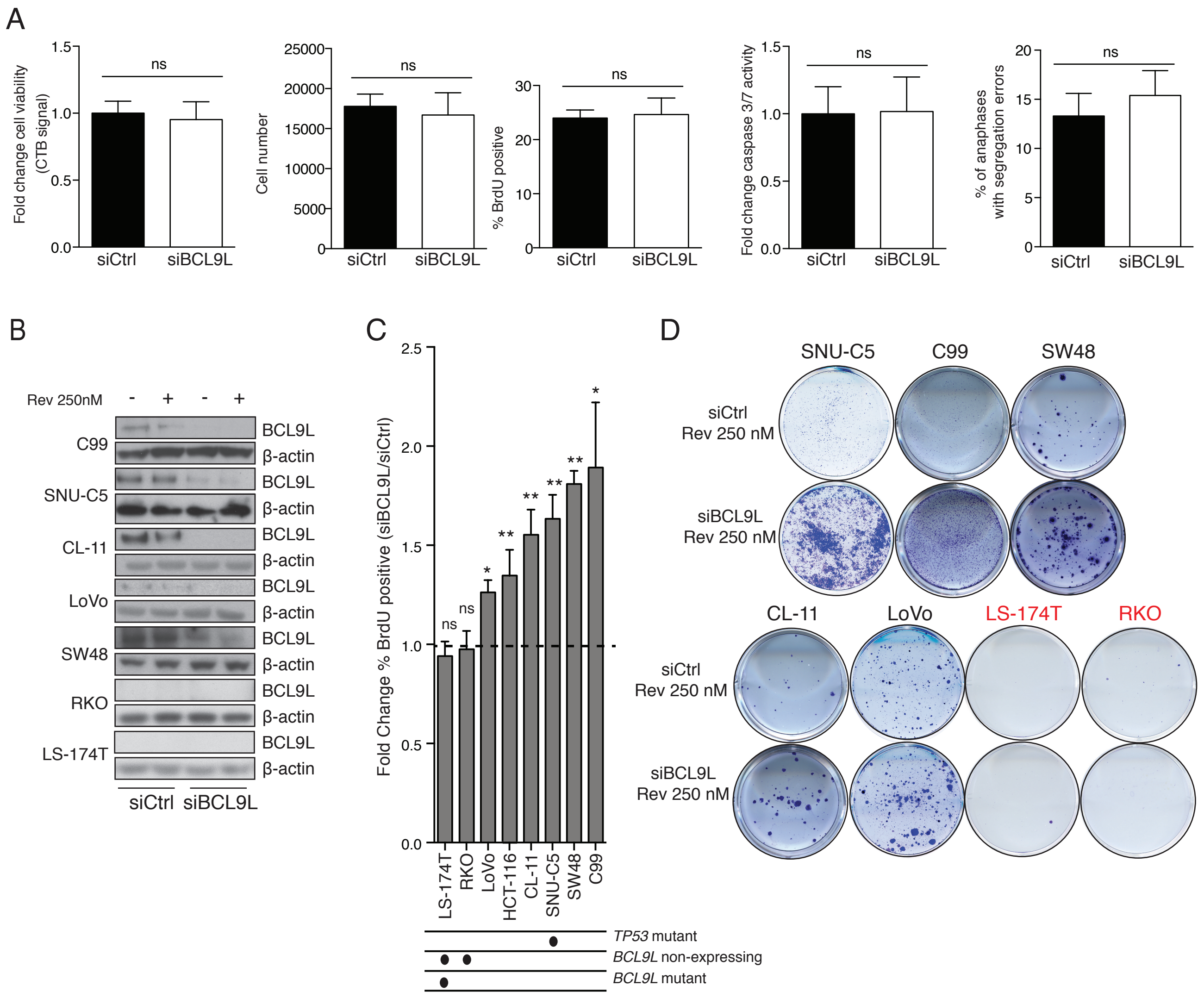

Figure S4. Related to Figure 2. (**A**) Basal effect of BCL9L knock-down on cell viability, cell number, BrdU incorporation, caspase 3/7 activity and endogenous segregation errors in the absence of reversine (n=3, p values calculated by paired Student's t-test). (**B)** Western-blot analysis of BCL9L protein levels in 7 near-diploid cell lines. Effect of 3 day reversine treatment and BCL9L knock-down are shown. (**C**) Effect of BCL9L depletion on the fraction of BrdU-incorporating cells in 8 reversine-treated near-diploid cell lines (n=3, no changes detected in untreated controls, p value calculated by paired Student's t-test). Cells were treated with 250 nM reversine for 3 days followed by 1 hr BrdU pulse. *BCL9L* and *TP53* mutation status is shown at the bottom. (**D)** Colony forming efficiency of 7 near-diploid CRC cell lines after BCL9L siRNA transfection and reversine treatment. After 72 hr of siRNA transfection, cells were plated in serial dilutions in the presence and absence of 250 nM reversine for 5 days and grown until appropriate colony size was achieved. LS-174T and RKO do not express BCL9L and accordingly do not benefit from BCL9L knock-down.

Error bars denote SD. ns=not significant, *p<0.05, **p<0.001.

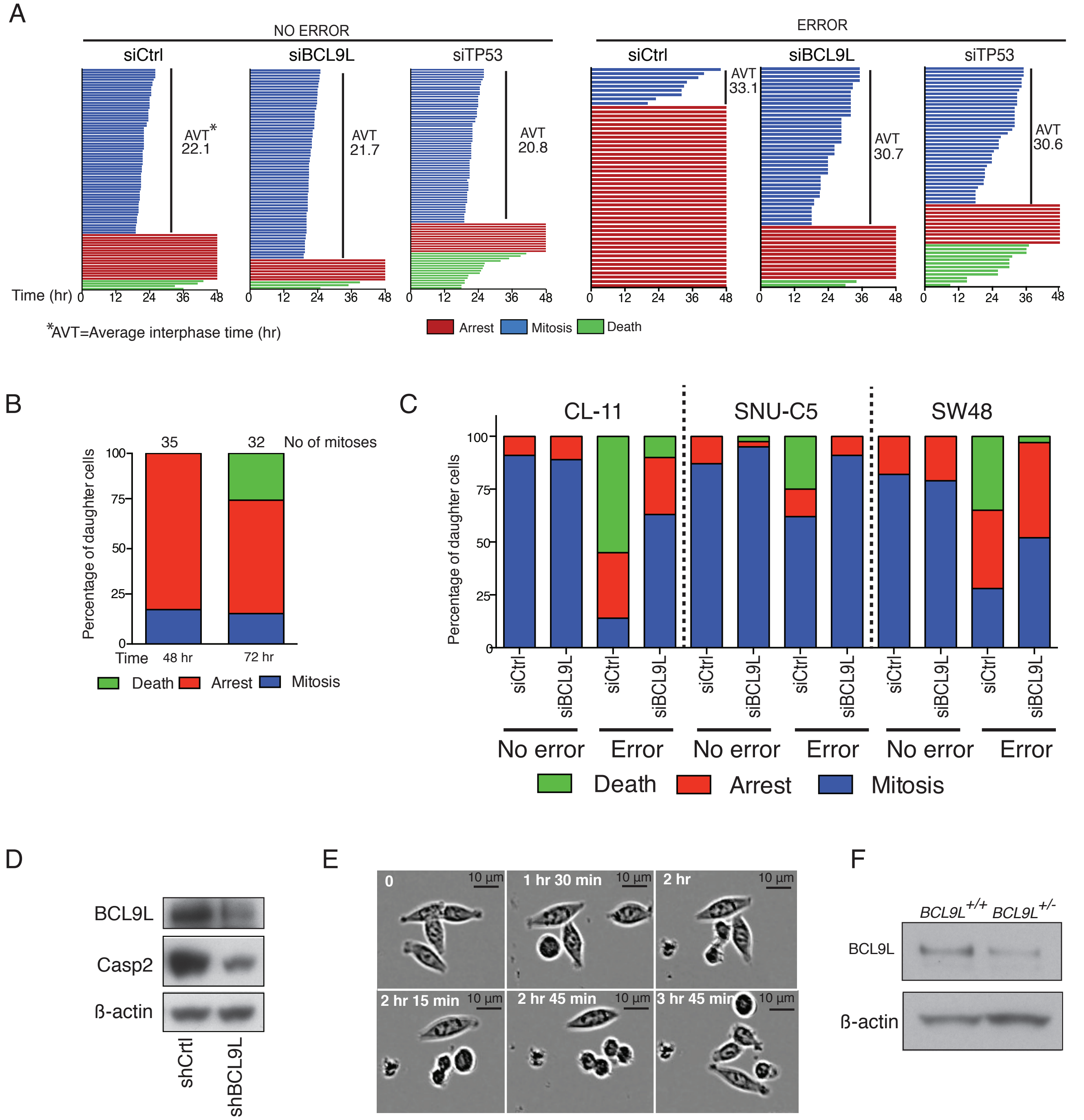

Figure S5. Related to Figure 2 and 3. (**A**) Single cell analysis of the effect of BCL9L and TP53 knock-down on post-mitotic daughter cell fate after normal mitosis or mitosis with endogenous segregation error (Time=0 marks the time of the first mitosis) (**B**) Post-mitotic daughter cell fate of HCT-116 after mitosis with chromosome segregation error. Live cell imaging was carried out for 48 or 72 hr. (**C**) Post-mitotic daughter cell fate of 3 near-diploid cell lines after a normal mitosis or with an endogenous chromosome segregation error. A minimum of 30 mitoses cells were analyzed. (**D**) Western-blot analysis of BCL9L and caspase-2 expression in HCT-116 cells stably expressing a *BCL9L* shRNA. (**E**) Representative images of mitosis in HCT-116 cells in reversine obtained by live-cell brightfield microscopy. All mitosis analyzed underwent normal cytokinesis and divided. (**F**) Levels of BCL9L protein in a *BCL9L*-WT clone and a *BCL9L*-mutant clone with a heterozygous 5 bp deletion downstream the HD3 domain as shown in 3A.

Movie S1: Two anaphases at 1 hr 33 min and 3 hr 33 min with segregation errors. Daughter cells arrest or die. Related to Figure 2

Movie S2: Anaphase with chromosomal bridge at 3 hr 15 min. Daughter cells divide and arrest. Related to Figure 2

Movie S3: Anaphase with lagging chromosome at 51 min. Both daughter cells divide. Related to Figure 2

Movie S4: Normal mitosis at 42 min. Both daughter cells divide. Related to Figure 2

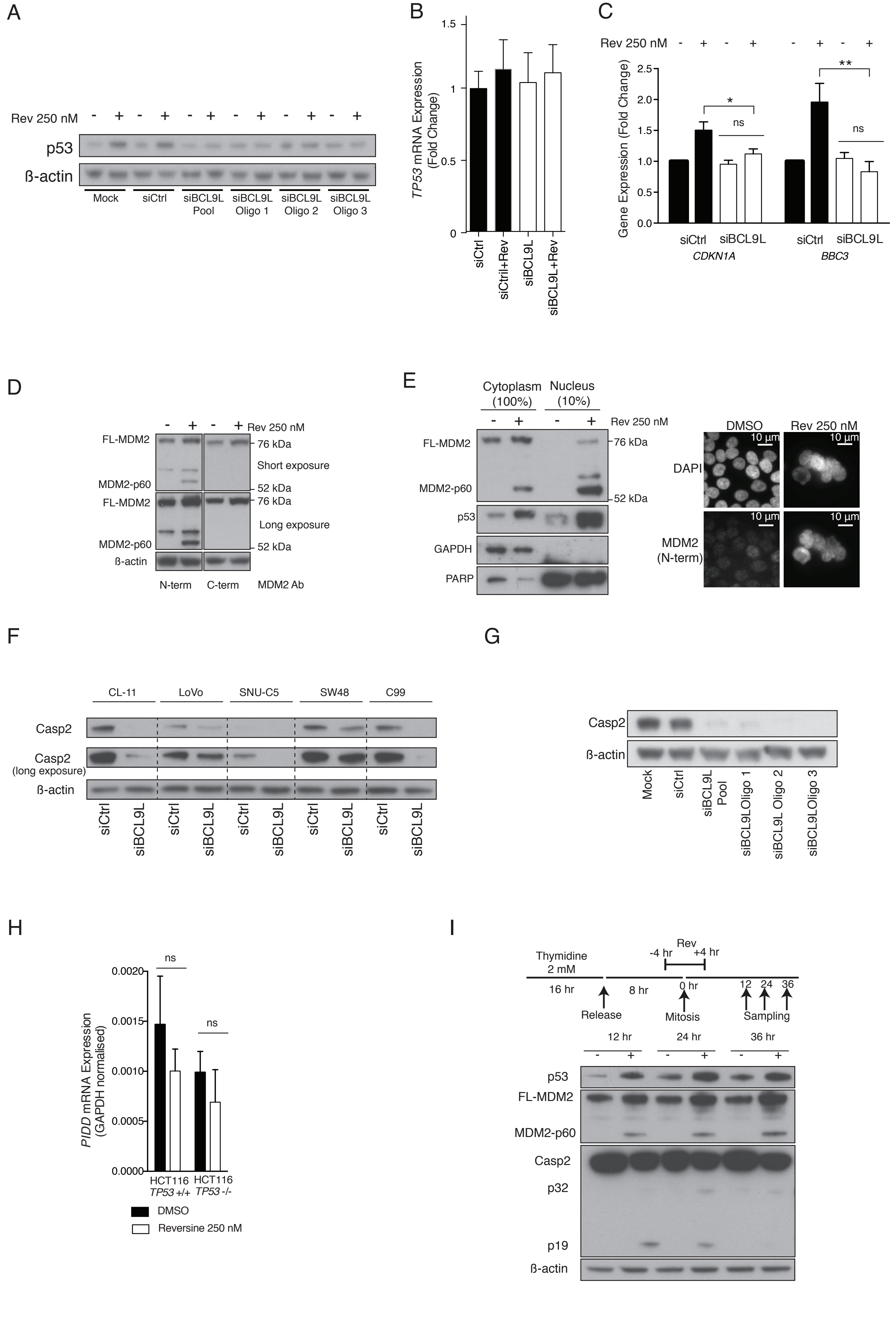
A
Rev 250 nM - + - + - + - + - + - +
p53
ß-actin
Mock
siCtrl
siBCL9L Pool
siBCL9L Oligo 1
siBCL9L Oligo 2
siBCL9L Oligo 3
B
TP53 mRNA Expression (Fold Change)
1.5
1
0.5
0
siCtrl
siCtrl+Rev
siBCL9L
siBCL9L+Rev
C
Rev 250 nM - + - + - + - +
Gene Expression (Fold Change)
2.5
2.0
1.5
1.0
0.5
0.0
*
ns
**
ns
siCtrl
siBCL9L
siCtrl
siBCL9L
CDKN1A
BBC3
D
- + - + Rev 250 nM
FL-MDM2
76 kDa
Short exposure
MDM2-p60
52 kDa
FL-MDM2
76 kDa
Long exposure
MDM2-p60
52 kDa
ß-actin
N-term
C-term
MDM2 Ab
E
Cytoplasm (100%)
Nucleus (10%)
- + - + Rev 250 nM
FL-MDM2
76 kDa
MDM2-p60
52 kDa
p53
GAPDH
PARP
DMSO
Rev 250 nM
DAPI
MDM2 (N-term)
10 µm
F
CL-11
LoVo
SNU-C5
SW48
C99
Casp2
Casp2 (long exposure)
ß-actin
siCtrl
siBCL9L
G
Casp2
ß-actin
Mock
siCtrl
siBCL9L Pool
siBCL9LOligo 1
siBCL9L Oligo 2
siBCL9LOligo 3
H
PIDD mRNA Expression (GAPDH normalised)
0.0020
0.0015
0.0010
0.0005
0.0000
ns
ns
HCT116 TP53 +/+
HCT116 TP53 -/-
DMSO
Reversine 250 nM
I
Thymidine 2 mM
16 hr
Release
8 hr
Rev
-4 hr
+4 hr
0 hr
Mitosis
12
24
36
Sampling
12 hr
24 hr
36 hr
- + - + - +
p53
FL-MDM2
MDM2-p60
Casp2
p32
p19
ß-actin

Figure S6. Related to Figure 5. (**A)** Reversine mediated induction of p53 in HCT-116 and effect of BCL9L knock-down with a pool of 3 siRNAs (Qiagen) and 3 individual single siRNAs (components of the pool) in HCT-116. (**B)** qPCR analysis of *TP53* mRNA expression in isogenic *TP53*-WT and null HCT-116 cells following reversine treatment and BCL9L depletion (n=3). (**C)** qPCR analysis of the p53 transcriptional targets *CDKN1A* (p21) and *BBC3* (PUMA) in HCT-116 cells and the effect of reversine and BCL9L knock-down (n=3, p values calculated by unpaired Student's t-test). (**D)** Immunodetection of MDM2 with antibodies against the N-terminal domain (left) or C-terminal domain (right) respectively. (**E)** Subcellular localization of full length-MDM2 and MDM2-p60 and p53 assessed by cellular fractionation and immunofluorescence (Note that nuclear protein represents 10% of cells relative to cytoplasmic). (**F)** Effect of BCL9L knock-down on caspase-2 expression in 5 near-diploid cell lines (2 exposures shown). (**G)** Effect of BCL9L depletion on caspase-2 expression with a pool of 3 siRNA oligonucleotides (Qiagen) and 3 individual siRNA oligonucleotides (components of the pool, marked in Supplemental Experimental Procedures). (**H)** Analysis of *PIDD* mRNA expression level by qPCR analysis following reversine treatment in HCT-116 *TP53*-WT and null cells (n=3, p values calculated by unpaired Student's t-test). (**I)** Time-course analysis of p53 accumulation, MDM2 cleavage and caspase-2 activation in synchronized HCT-116 cells transiently exposed to reversine during a single mitosis. Cells were synchronized at the G1-S transition with 2 mM thymidine for 16 hr, and released in fresh media to allow cell cycle progression. 250 nM reversine was added 4 hr before the estimated time for mitosis (8 hr after thymidine washout) and removed 4 hr after the estimated time for mitosis such that cells are only exposed to reversine during a single mitosis. Samples were collected 12, 24 and 36 hr later and lysed for western blot analysis.

Error bars denote SD. ns=not significant, *p<0.05, **p<0.001.

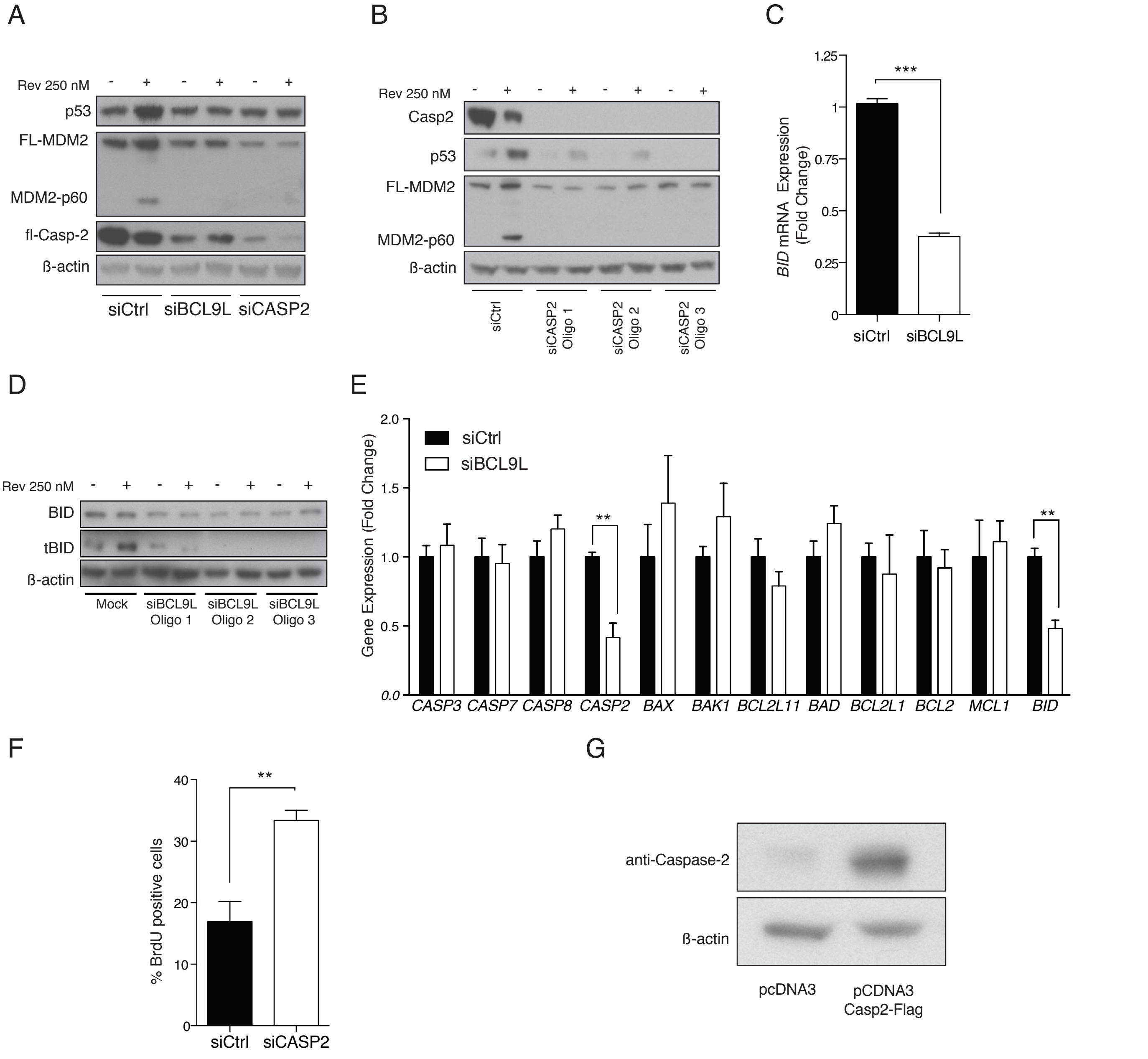

Figure S7. Related to Figure 5. (**A)** Effect of BCL9L and caspase-2 depletion on MDM2 cleavage and p53 stabilization following reversine treatment of HCT-116 cells. (**B)** Effect of caspase-2 knock-down on p53 stabilization and MDM2 cleavage with 3 different siRNA duplexes, and treated with reversine where indicated (Qiagen siRNAs used identified in Supplemental Experimental Procedures). (**C)** Analysis of *BID* mRNA expression in *TP53*-null HCT-116 by qPCR (n=3, p values calculated by unpaired Student's t-test). (**D)** Effect of *BCL9L* silencing with 3 different siRNA sequences on *BID* expression and cleavage in *TP53*-null HCT-116 determined by western blot (same oligonucleotides as the ones used in S6A and S6G). (**E)** qPCR analysis of mRNA expression of a selection of various initiator and effector caspases and components of the BCL2 family of mitochondrial apoptotic proteins (Fold change relative to siRNA control, n=3, p values calculated by unpaired Student's t-test). (**F)** Effect of caspase-2 depletion on cell proliferation as determined by the fraction BrdU-incorporating cells in 250 nM reversine treated HCT-116 cells (n=3, p values calculated by paired Student's t-test). (**G)** Caspase-2 protein levels in pcDNA3-Caspase2-Flag transfected HCT-116.

Error bars denote SD. **p<0.001, ***p<0.0001.

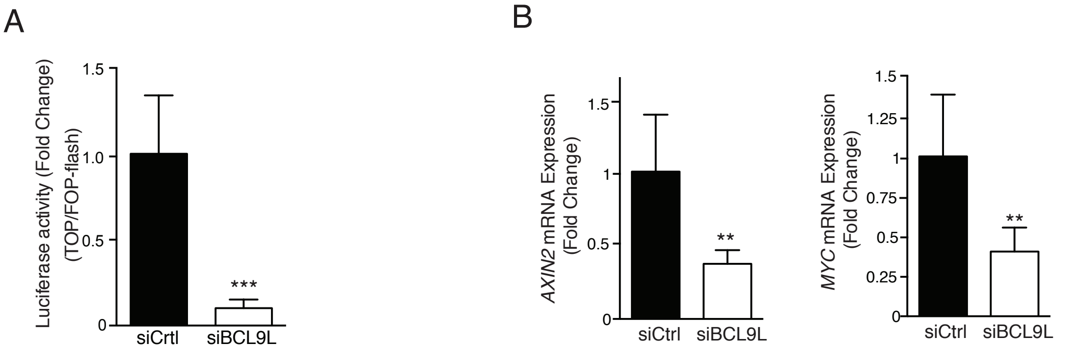

Figure S8. Related to Figure 6. (**A)** Effect of BCL9L knock-down on Wnt signaling (TOP/FOP-Flash luciferase reporter). (**B**) Gene expression analysis of the effect of BCL9L depletion on the Wnt signaling targets *AXIN2* and *MYC*.

p values calculated by unpaired Student's t-test. Error bars denote SD. $**p<0.001$, $***p<0.0001$.

SUPPLEMENTAL EXPERIMENTAL PROCEDURES

Patient samples, whole-exome sequencing, Ion-Torrent targeted sequencing and SNP array

Tissue collection was approved by an ethics committee (NRES Committee South Central-Oxford B, REC reference 05/Q1605/66), and all individuals included in this study had provided written informed consent for the analysis presented.

For whole exome sequencing, DNA was extracted from fresh frozen colorectal adenocarcinomas and adjacent normal colon. Exome enrichment was carried out with SureSelect 38Mb+. Exome sequencing was performed on an Illumina GAIIx in 72 bp paired end mode. FastQ files were aligned to hg19 reference sequence using bwa 0-5.9r16 with a seed length of 72 bases and all other settings left as default (in particular up to three mismatches were allowed per read). Sam/Bam files associated with the same sample were merged and converted to pileup files using samtools 0.1.16. Point mutations were identified from these pileup files using in-house scripts, which applied a series of thresholds. Variant calls were validated by Sanger sequencing in tumour and normal colon DNA samples.

For Ion Torrent analysis we designed oligonucleotide libraries for a panel of genes including *APC*, *TP53*, *CTNNB1*, *BCL9L* and *BCL9*. Putative variants were targeted for validation using the Ion Torrent platform, with alignment of raw reads carried out on the Torrent Server using Tmap, with a minimum detection frequency of required before the validation was considered to be successful. Somatic status of mutations was validated by Sanger sequencing in tumour and normal colon DNA samples.

For SNP array analysis of tumour samples, DNA was run on Human 610 Quad v1 or Human CytoSNP12 v2.1 arrays in accordance with the Illumina Infinium assays manual protocol (Illumina). Genome Studio software (Illumina) was used to retrieve LogR Ratios and B allele frequencies and calls for copy number changes and LOH status were obtained with OncoSNPv2 software.

For multi-region SNP array of xenografts, we used the Affymetrix CytoScan HD SNP array platform. All analysis was performed in the R statistical environment version 3.2.1. Arrays were processed along with a batch of 15 arrays of normal blood acquired from GEO, GSE53799, using the Aroma Affymetrix CRMAv2 algorithm, and the B-allele fraction (BAF) was adjusted using the CalMaTe algorithm. Tumor LogR data was segmented using the PCF algorithm. Thresholds for calling copy number gain and loss was determined from analysis of the distribution of segmented LogR scores, and set at -0.3 for loss and 0.15 for gain on the uncentered distribution.

Ploidy analysis (DNA Image Cytometry)

Image Cytometry analysis was performed according to methods described in Dunn et al. (2010). In brief, 50 µm sections were cut from FFPE blocks, dewaxed in xylene, rehydrated in ethanol series and digested with proteinase XXIV (Sigma-Aldrich). Sample was washed in PBS, filtered with a 40 µm strainer and resuspended in PBS. The nuclear suspension was spun onto Superfrost slides Plus (positively charged, VWR), dried for 1 hr placed in HCl 5 M for 1 hr and stained with Feulgen-Schiff using published methodology (Bocking et al., 1995). Integrated optical density was measured using a Fairfield DNA Ploidy System. A minimum of 600 nuclei were analyzed, sorted into 4 separate categories (cancer cells, lymphocytes, plasma cells and fibroblasts). Lymphocytes were used as control to determine diploid peak position. Histograms were finally analyzed according to European Society for Analytical Cellular Pathology (Bocking et al., 1995, Haroske at al., 2001).

FISH (FFPE specimens)

5 µm sections were dewaxed in xylene, rehydrated in ethanol series, permeabalized in 1 M sodium thiocyanate at 80°C for 10 min and washed 2 times 10 min in PBS. Samples were then digested in 0.4% pepsin in 0.1 M HCl at 37°C for 10 min followed by quenching with 0.2% glycine in PBS for 5 min and 3 washes in PBS over 15 min. Samples were then fixed in 4% PFA for 10 min, washed 3 times in PBS over 15 min and dehydrated in ethanol series. 1 µl of chromosomal centromeric probes (CEP2 and CEP15, Abbot Molecular Inc.) was diluted in 10 µl of provided hybridization buffer, placed onto the sample, covered with coverslip, sealed with rubber solution glue, placed at 95°C for 10 min and transferred overnight to a 37°C humidified incubator. Slides were then washed 3 times with 0.5xSSC (20xSSC composition is 17.5% NaCl, 8.8% tri-sodium citrate) over 15 min at 37°C and 3 times with PBS over 15 min at room temperature. Finally, slides were then counterstained with DAPI, dehydrated in ethanol series and mounted in fluorescence compatible medium.

FISH (Cell lines)

Cells were grown on Poly-Lysine coated glass slides with required culture medium, washed with PBS, placed in 37°C hypotonic solution (0.4% KCl and 0.4% sodium citrate) for 7 min and fixed in 3:1 methanol/acetic acid at -20°C for 15 min. Cells fixed onto slides were denatured in 70% formamide,

2X SSC at 70°C for 90 seconds and then transferred to 70% ethanol at -20°C. CEP probes (CEP 1, 2, 8, 15, Abbot Molecular Inc.) were denatured at 95°C then placed onto slides, covered with coverslips and sealed with rubber solution glue. Hybridization was performed in a humidified incubator overnight at in 37°C. Slides were then washed 3 times with 50% formamide, 2X SSC over 15 min at 42°C, 3 times with 2x SSC over 15 min at 42°C, once with 4x SSC, 0.02% Tween for 5 min and finally once in PBS. Slides were counterstained with DAPI, dehydrated in ethanol series, and mounted in with fluorescence compatible media.

Metaphase spreads

Cells were treated with 10 µl/mL Colcemid (GIBCO) for 90 min, trypsinised and allowed to swell in hypotonic solution (0.4% KCl and 0.4% sodium citrate) for 7 min at 37°C. Cells were then spun down and the pellet resuspended in methanol/acetic acid (3:1). The suspension of fixed cells was dropped onto glass slides to allow cell breakage and the existence of chromosome spreads confirmed under the microscope. Metaphase spreads were stained with a human pan-centromeric FISH (Kreatech, Leica) probe with enzymatic treatment as described by the manufacturer.

TCGA mutation and copy-number data

All mutation data were downloaded from the Broad Institute MAF dashboard (https://confluence.broadinstitute.org/display/GDAC/MAF+Dashboard). Mutations were filtered to ensure that each variant had at least five tumor reads and coverage of ≥30.
Affymetrix SNP 6.0 arrays for TCGA for Colorectal Adenocarcinoma were obtained from TCGA (https://tcga-data.nci.nih.gov/tcga). Allele-specific integer copy numbers and purity estimates, were obtained using ASCAT.

Cell lines, siRNA, shRNA transfections and DNA constructs

Cell lines were provided by Cell Services at The Francis Crick Institute or by collaborators, mycoplasma tested and authenticated by STR profiling and comparison against published profiles.
siRNA pools were obtained from Qiagen (GeneSolution) and Dharmacon (siGENOME) (sequences provided below). Tranfections were carried out to a final concentration of 40 nM with Lipofectamine RNAiMAX transfection reagent (Life Technologies) to a final 1/400 dilution.
For stable silencing of *BCL9L* we used a GIPZ lentiviral construct (Clone V3LHS_368414, target sequence AGCAGATGAACATGATGAT, ThermoFisher).
TOP/FLOP-Flash luciferase reporter constructs were purchased from Millipore. *BCL9L* cDNA was cloned into a pEGFP-N1 for EGFP C-terminal fusion (ClonTech). pcDNA3-Casp2-Flag construct was obtained from Addgene. Constructs were transfected using Lipofectamine-2000 (Life Tecnologies) according to manufacturers instructions.

siRNA sequences

siGENOME

Gene Symbol	Accession Number	Target Sequence
ADAMTS9	NM_182920	CGACAAAUGUGAUACCUUA<br>GGACGUAGAAUGAAAUUUA<br>GGAAAGCACUGCAAGUAUG<br>GGACACAAAUGAUAUCUGU
ADAMTSL3	NM_207517	GGACAGAGGUCAUCAAUAU<br>GAACAUAAUCCUUGGACUG<br>ACAAUCCGAUGUCCUGUAA<br>GUAGAGGAAUCCAUGCAUG
APCDD1	NM_153000	GCUCAAACAUCUCCACAAU<br>CUUCAAGGCCUACCAAUUU<br>CAAACUACCUCACACGGAG<br>GCCCAGAGUUCAUCACAAG
BCL9L	NM_182557	GAACAGCAGUGGCGUGAUG<br>GCUGAUGCCUUCACAGUUU

		GACCUCACCAUCAGUAUUA<br>CAGGCAACCUCAACAUGAA
BBS7	NM_018190	UAACAGGCCAGUUCAGUUU<br>UUACAGGGAUCUGAUGUGA<br>GCGCUUAUACAGAUUACUA<br>GUUCUACGAUUUGAUCAGA
CCNT1	NM_001240	UAUCAACACUGCUAUAGUA<br>GAACAAACGUCCUGGUGAU<br>GCACAAGACUCACCCAUCU<br>UAGUAUACAUGCAUCGAUU
CNTN3	NM_020872	GCUCAAAGUUGUUGCUUCU<br>GAGUUCAUCUGGUGAUUUA<br>GCUGAGAAUUCACGAGGAA<br>GAAGAUAGUCGGAGAUUUG
DMD	NM_000109	GAAUGUUUAUGAUACGGGA<br>GCAAGUGGCAAGUUCAACA<br>GUCAGAUUCUCAGCUUAUA<br>CAAGACAGUUGGGUGAAGU
DNAH5	NM_001369	GGACAUCACUGAAUAUUGA<br>AAACUGAGCUUGUACAUGA<br>GAAGUUCCCUGCCCUAAUU<br>CGAAUGAGAUAGUGCGACA
DOCK11	NM_144658	UAAAUGAGCGGCUAAUUAA<br>GAUCAACACCGACAGUUUA<br>CGACAGAGUUCUACAAGGA<br>CGAAACAGAUCAGGAGUAA
DSCAM	NM_001389	GCGCAUAAGUGAAAUCAUA<br>GGACGGGAUUUAUGGCAAA<br>ACGGGAGCCUUGUAUAUUA<br>GUCAAAGCACGCACAAUUA
EPB41L3	NM_012307	GCUCGAAUAUCAGCAAUUA<br>GAAAGUAUGUGUUGAGCAU<br>CGGAACAACUUUUACAUUA<br>GGGCUUACGUAUCGAGAUG
FLNA	NM_001456	CCAACAAGGUCAAAGUAUA<br>GCAGGAGGCUGGCGAGUAU<br>GUAUGGAGAUGAAGAGGUA<br>UCACAGAAAUUGACCAAGA
GDF5	NM_000557	GGAAGGCACUGCAUGUCAA<br>AUAAGACCGUGUAUGAGUA<br>CCAAUAAGCUUGCUCGCUC<br>ACGUGGUGUAUAAGCAGUA
HDLBP	NM_005336	CACCAUCGCUUUGUUAUUG<br>GAAGCGACACCGUUGUUAU<br>CGGCAAAGCCAUUCGCAAA<br>GGUUGGAGCAUGACGUGAA
KDM5B	NM_006618	CCACAGAGCUUGUUGAGAA<br>GCAGAGGCCAUGAAUAUUA<br>CAACAGAACCUCAUAUUUG<br>GGAGAUGCACUUCGAUAUA
MYO3A	NM_017433	GAAAGGCAAUAACAUUCUA<br>GCAAUUGGAUACCACUUAU<br>GAACAGCAAUUGGAUACCA<br>GAAGAGGGGUGAAAGAAUG
MYO3B	NM_138995	GAAAUGAUGUUUACACCAA<br>GUACAUAGCUGAUGAAACU<br>GGAAAGGUAUUAUAUGAUG<br>GGGCAAGAAUCUCUGAAUA
MYCBP2	NM_015057	GGAGUAAGCUGACUAACUU

		GGACAGGUCUUCACAUUUG<br>GAAAUGAGAUAUCUACGUU<br>GAAGGAAGCUCGAUAUAUU
NALCN	NM_052867	GCUCAUCUCUCCACAAAUU<br>GAUAGGAACUAGUAUAUUC<br>GGACUUCGGUGGAGUAAUG<br>GGACAGAUUUACUACGUUU
NFATC2	NM_013986	CCAAUAAUGUCACCUCGAA<br>GCAGAAUCGUCUCUUUACA<br>GCGGGGAUCUUGAAGCUUA<br>UCAUGUACUGCGAGAAUUU
PCDH11X	NM_014522	GACCUUAACUUGUCGCUGA<br>CCAGAGAACUCGGCUAUAA<br>GGAAUAAACGGAGUUCAAA<br>GCAAGUGAGUGUUACUGAU
PIK3C2B	NM_002646	GCCGGAAGCUUCUGGGUUU<br>CAAGAGCUCUGGCCGAAUC<br>GCUGAGACCCUGCGUAAGA<br>GCUACCAGCUAUGAAGAUU
PKHD1	NM_138694	CGAGAUAGCUGUACUUUCA<br>CCAAUUACCGUCAAGAUUA<br>GGUAUGGUCUCUUUGUAUA<br>GGAAGGUGUUAGCCUGAUA
RABGAP1	NM_012197	GAUGGAAAGUCGUAUGUUA<br>GAAUUAGCCUGCAACAUGA<br>GUAGAGAAAUACCGCAUUC<br>AGAAAUGUGCCGUCGGGAA
RNF17	NM_031277	GAACUAUGCUUGUUAAUGA<br>GCAAGAGCAUUACAAUUAU<br>GAACUUUCUUGUUACGAUA<br>GAAAUUAGACAGAAUGUUC
SYCP2L	NM_001040274	GAAGAUGAACCUCUGCUAA<br>CCUCGUAGAUGGCCUGAAA<br>GGACUGAUCCCAAAGCUAG<br>UGUGUAGACUGACGAUUAA
SLC30A2	NM_001004434	GAGCAUGGGUGUCCUAGUG<br>GAGAAGUCGUUGAGAUCUU<br>GAGAUGUGAUCCUGGUGUU<br>CUGCAUAUCUGGGCACUGA
TP53	NM_000546	GAGGUUGGCUCUGACUGUA<br>GCACAGAGGAAGAGAAUCU<br>GAAGAAACCACUGGAUGGA<br>GCUUCGAGAUGUUCCGAGA
UGGT1	NM_020120	GAACAGAUCUGAAAGAGUU<br>CAAGGGAACUUUAGGAUUA<br>CAAAGGAGAUCACAGAUAU<br>GCUAGACCCUGAUGAGUUA
XIRP2	NM_152381	CAACAGAAGCUGCCCGCAA<br>GAAGAGAUGUGGAGUUCGA<br>GCGCAGGAUUGAACGCUUU<br>GAAAUUACUUCUUCCCGUA
ZFHX3	NM_006885	GUAUAAACCAAACGAGUUA<br>GUACAGAGACCACUACGAU<br>CCACUAUGCUAGAAUGUGA<br>GAACAAGGUUUACGGACUA

Qiagen GeneSolution

Gene Symbol	Accession Number	Target Sequence
BCL9L	NM_182557	CCCGACGGCUACCACCGCCAA*<br>CAGAGCUGAAGCCAUAUACGA*(3'utr)<br>CACCUGCAACGUGGGCUCGAA*<br>ACCCACAAUUGUAAUGUAGCA
BID	NM_001196	CAGGGAUGAGUGCAUCACAAA<br>AAAGACAAUGUUAAACUUAUA<br>UAGGGACUAUCUAUCUUAAUA<br>AACUUCCAAUUUCUAGCCAUA
CASP2	NM_032982	AACAUCUUCUGGAGAAGGACA*<br>AACCGAGUGGUGCUAGCCAAA*<br>UUGGUCCACCUUCCAGCACAA<br>CAGGAUCAUGUAAAUGCUCAA*
ZFHX3	NM_006885	CGGCGUGAGCUUACAAAUGAU<br>AACGGACACCUUCAGAUUGUA<br>CAGAGCGCUCUUCAAAGCCAA<br>AAGAACAAGGUUUACGGACUA

(*). Oligonucleotides with the same effect as the pool of 4 and used in deconvolution experiments

Reversine-tolerance assays

Cell-viability and BrdU assays were performed by seeding cells in transfection medium with reversine 250 nM for 72 hr. For cell viability, 20 µl of Cell Titer Blue (Promega) was added in 100 µl of culture media for 2 hr and fluorescence measured according to manufacturers instructions. For BrdU analysis, BrdU was added to a final concentration of 100 nM for 1 hr. Cells were trypsinized, fixed in 70% ethanol, washed with PBS, resuspended in 2 M HCl for 20 min and washed twice in PBS. Fixed cells were then stained with anti-BrdU primary antibody (1/50, Becton-Dickinson) washed with PBS and finally stained with secondary goat anti-mouse Alexa Fluor488-conjugated antibody (Life Technologies). Finally cells were resuspended in 1 ml of PBS, treated with 50 µl of ribonuclease A (100 mg/ml stock, Sigma-Aldrich) and 150 ul of propidium iodide (50 µg/ml stock, Sigma-Aldrich) and subjected to FACS analysis.

For colony formation assays cells were transfected with siRNA for 3 days, then plated 6 serial 1/10 dilutions at a starting concentration of $2x10^5$ cells (6-well plates) in 250 nM reversine for 5 days. Reversine-containing medium was replaced by drug free medium and colonies were allowed to grow to an appropriate size. For long-term treatment (CRISPR and shRNA) experiments we used reversine 125 nM for 15 days.

Caspase 3/7 enzymatic activity assay

For determination of caspase3/7 enzymatic activity we used the ApoOne system (Promega) according to manufacturers instructions.

Endogenous segregation error screen (Live-cell imaging)

For live cell imaging, we generated stable HCT-116 expressing H2B-RFP. 48 hr siRNA knock-down was carried out on microscopy-compatible cell chambers. Imaging was performed using a DeltaVision RTsystem (Image Solutions, Applied Precission) equipped with temperature, humidity and CO2 control. Time-lapse images were acquired for each point with z-stacks steps of 2 μm to image the whole cell, every 3 min for 6 hr, which allows adequate spatio-temporal resolution to identify segregation errors. Images were acquired every 15 min during the rest of the experiment up to 48 hr to determine if daughter cells divided further, arrested or died.

Segregation error analysis

Cells were grown on poly-lysine (1/8 dilution, Sigma-Aldrich) coated coverslips and fixed in 10% Triton X-100, 1M PIPES, 0.5M EGTA, 1M $MgCl_2$, 4% formaldehyde. Coverslips were stained with rabbit anti-β-tubulin antibody (1/1000, Abcam), human anti-centromere antibodies (1/250, Antibodies Incorporated) and DAPI (Roche). For secondary staining we used goat anti-rabbit Alexa Fluor 488 and goat anti-human Alexa Fluor 594 (Molecular Probes, 1/500). All antibodies were diluted in 3% BSA 1xPBS. Manual examination of segregation errors in anaphase was carried out with z-stacks acquired with an Olympus DeltaVision RT microscope (Applied Precision) at 60x or 100x.

Western-blot and quantitative PCR

Cell lysates were made with a buffer containing 100 mM NaCl, 50 mM Tris, 10% glycerol and 0.2% NP40 and protein concentration measured with Bradford reagent. Protein electrophoresis and transfer onto PVDF membrane were carried out using NuPAGE NOVEX system (Life Technologies). Electrophoresis was performed with NuPAGE Bis-Tris 4-12% gradient gels in 1x NuPAGE MES running buffer and transferred for 90 min at 150V in 1x NuPAGE transfer buffer, 20% methanol. For BID detection we used 12% NuPAGE Bis-Tris non-gradient gels and for BCL9L detection we used NuPAGE 3-8% Tris-Acetate gels and transfer with pH10 transfer buffer (19.2 mM Tris, 2.5 mM Glycine, 0.01% SDS and 10% methanol). Primary antibodies used were mouse anti-p53 (1/1000, DO1 clone, Santa Cruz Biotechnology), mouse anti-MDM2 (1/1000, SMP14 clone, Santa Cruz Biotechnology), rabbit anti-C-terminal MDM2 (1/1000, C18, Santa Cruz Biotechnology), rat anti-caspase-2 antibody (1/1000, Enzo Life Technologies), rabbit anti-BCL9L (1/500, Abcam), rabbit anti-BID (1/1000, ab113110, Abcam) and HRP-conjugated anti-β-actin (1/50000, A3854, Sigma-Aldrich), rabbit anti-PARP (1/1000, 46D11, Cell Signaling Technology). For primary antibody detection we used secondary HRP-swine anti-rabbit (1/50000, Dako), HRP-goat anti-mouse (1/50000, Dako) and HRP-goat anti-rat (1/10000, GE Healthcare Life Sciences).

Taqman gene-expression assays (Applied Biosystems): *GAPDH* (Hs99999905), *BID* (Hs01026792), *CASP2* (Hs00892481), *PIDD* (Hs00611142).

PCR primers for SYBR green quantitative PCR:

AXIN2_F	gctgacggatgattccatgt
AXIN2_R	actgcccacacgataaggag
BAD_F	cgagtttgtggactcctttaaga
BAD_R	caccaggactggaagactcg

BAK1_F	cctgccctctgcttctga
BAK1_R	ctgctgatggcggtaaaaa
BAX_F	caagaccagggtggttgg
BAX_R	cactcccgccacaaagat
BBC3_F	gacctcaacgcacagtacga
BBC3_R	gagattgtacaggaccctcca
BCLXL_F	agccttggatccaggagaa
BCLXL_R	agcggttgaagcgttcct
BCL9L_1F	tggattcagaggccaaagag
BCL9L_1R	cccactgtacggctgctt
BIM_F	catcgcggtattcggttc
BIM_R	gctttgccatttggtcttttt
CASP3_F	tgtgaggcggttgtagaaga
CASP3_R	gggctcgctaactcctcac
CASP7_F	ccgagacttttagtttcgcttt
CASP7_R	cctgatcatctgccatcgt
CASP8_F	ggtcacttgaaccttgggaat
CASP8_R	tttctgctgaagtccatcttttt
CASP9_F	aagcccaagctcttttcatc
CASP9_R	actcgtcttcaggggaagtg
CDKN1A_F	ccgaagtcagttccttgtgg
CDKN1A_F	catgggttctgacggacat
MCL1_F	Aagccaatgggcaggtct
MCL1_R	tgtccagtttccgaagcat
MYC_F	gctgcttagacgctggattt
MYC_R	taacgttgaggggcatcg
TP53_F	aggccttggaactcaaggat
TP53_R	cccttttggacttcaggtg

Subcellular compartment protein enrichment.

For enrichment of nuclear and cytoplasmic protein fractions we used Qproteome Cell Compartment Kit (Qiagen) according to manufacturers instructions.

CRISPR genome editing and reversine-resistant colony selection

We designed a guide RNA construct targeting nucleotides 2542-2561 (AGTACTACGAAGAGAAACGG) of *BCL9L* (cDNA sequence NM182557) according to protocol described by Cong and colleagues (2013) by cloning a protospacer sequence into pX330-hSpCas9. transient transfection system (double-strand break). Cells were transfected with the *BCL9L* targeting construct for 48 hr and seeded in serial dilutions in reversine-containing media. After 2 weeks in 125 nM reversine resistant colonies were collected and *BCL9L* genotyped by Sanger sequencing.

Chromatin-immunoprecipitation

A 15 cm dish of 70% confluent HCT-116 was fixed with 1% formaldehyde in culture medium for 10 min at room temperature and quenched by adding 2.14 ml of 1.5 M glycine. Cells were scraped in 10 ml PBS with protease and phosphatase inhibitors and pelleted at 1500 rpm for 10 min at 4°C. The cell pellet was resuspended in 2 ml of buffer A containing NP40 (5 mM PIPES-NaOH, pH8, 85 mM KCl, 0.5% NP40 and inhibitors), incubated with rotation at 4°C for 10 min and pelleted at 3700 rpm for 10 min at 4°C. The pellet was resuspended buffer A without NP40, incubated with rotation at 4°C for 10 min and pelleted at 3700 rpm for 10 min at 4°C. The pellet was resuspended in lysis buffer B (50 mM Tris-HCl, pH8, 1% SDS, 10 mM EDTA and inhibitors), incubated with rotation at 4°C for 10 min and pelleted at 3700 rpm for 10 min at 4°C. Samples were sonicated in a Bioruptor at low intensity for 5 cycles (30 seconds on/30 seconds off), incubated at 4°C for 10 min and pelleted at 3700 rpm for 10 min at 4°C. The chromatin solution was diluted 1/5 with IP buffer (16.7 mM Tris-HCl, pH8, 167 nM NaCl, 1.2 mM EDTA, 1.1% Triton X100, 0.01% SDS and inhibitors). The chromatin solution was pre-cleared with 30 µl of Protein G-Sepharose FastFlow beads (Sigma) for 1 hr at 4°C. Sepharose beads were pelleted and 2 ml of the chromatin solution were incubated with 40 µl of rabbit antiTCF4 antibody (C48H11, Cell Signaling Technology) or with an equivalent amount of rabbit IgG (2729, Cell Signaling Technology) simultaneously with 30 µl of Protein G-Sepharose beads at 4°C overnight. Beads were washed 3 times with a low salt buffer (20 nM Tris HCl, pH8, 150 mM NaCl, 1% Triton X-100 and 0.1% SDS) and once with high salt buffer (same composition except 300 nM NaCl). Immune complexes were eluted overnight in 250 µl of elution buffer (10 mM Tris-HCl, pH8, 300 mM NaCl, 5 mM EDTA, 1% SDS) at 65°C. The supernatant was incubated 2 µl RNAase A (100 mg/ml) for 1 hr at 37°C followed by 2 µl Proteinase K (10 mg/ml) at 65°C for 2 hr and finally DNA purified with Qiagen PCR purification columns. Enrichment of immunoprecipitated DNA in the *CASP2* promoter was studied by qPCR using the following primers:

Primer pairs designed upstream the TCF4 binding site in the CASP2 promoter (ENCODE, chr7:142,985,169-142,985,544):

1F	ATGGGTTGGTATGAAGGCCA
1R	AGCTGTTAGAGACTCTTCGCA
2F	TGCCTCACTCCTAAGCTCTG
2R	GCAGCACACAATTCCAGGAA
3F	ATCTGTCCAGGGCCTTGTC
3R	TGGCGATAACTTCACGGAGA

Primers designed within the TCF4 binding site:

4F	TCTCAGTCTCCTTCGCAGC
4R	CCTATGGCGATAACTTCACGG
5F	AATCGGATTCCCAGGCCG
5R	CAGACAAAAGGAGGGGAAGG
6F	GTCTGAGGGGAGGGATGTG
6R	CCATTTCCCGCTTTTCCCG
7F	AAAAGCGGGAAATGGCGG
7R	TTCCCTACTTACCTGCGTCC

Primer pairs designed downstream the region of TCF4 binding site in the CASP2 promoter (5'UTR region and coding sequence):

8F	ATGGCCGCTGACAGGGGACG
8R	GCAGGAAGCGCGGACACG
9F	CAAGGGTGGGACGCCCGC
9R	CTGCAAAGACCTCCCCTTTAG
10F	CGACGAGACCTTCTGCAGAC
10R	GTACTCAATTTCCAAACCC

Mouse xenografts

HCT-116 cells infected with lentiviral shRNA targeting human *BCL9L* and control shRNA were grown in 125 nM reversine for 15 days and surviving colonies were trypsinized and expanded for two weeks. Two million cells were injected subcutaneously into nude (*nu/nu*) mice according to the UK Home Office rules and regulations.

Statistical methods

Data was analyzed using Prism 6.0 by two-tailed Student's t-test and Fisher's exact test. For the analysis shown in Figure S2B, on the basis of the observed probability for loss, given by the percentage of genome that is lost in that sample, we generated an aberration state (loss or no loss) for each sample separately. A pointbiserial correlation between aberration state and wGII was then calculated across samples. This process was repeated 10,000 times and a p value was obtained for *BCL9L* by counting the percentage of permutations showing a greater correlation coefficient than that observed for *BCL9L*.
For statistical analysis of tissue FISH analysis, centromere counts as established by FISH were tabulated and difference in ploidy was assessed using a two-tailed Fisher's exact test. All analyses were performed in R 2.12.2, with Monte Carlo simulation used to compute the p values for the Fisher's test.
For statistical analysis of metaphase spreads, total chromosome numbers were analyzed by two-sided Wilconxon rank sum test and performed in R 3.2.3.

SUPPLEMENTAL REFERENCES

Bocking, A., Giroud, F., and Reith, A. (1995). Consensus report of the ESACP task force on standardization of diagnostic DNA image cytometry. European Society for Analytical Cellular Pathology. Analytical cellular pathology : the journal of the European Society for Analytical Cellular Pathology *8*, 67-74.

Cong, L., Ran, F. A., Cox, D., Lin, S., Barretto, R., Habib, N., Hsu, P. D., Wu, X., Jiang, W., Marraffini, L. A., and Zhang, F. (2013). Multiplex genome engineering using CRISPR/Cas systems. Science *339*, 819-823.

Dunn, J. M., Mackenzie, G. D., Oukrif, D., Mosse, C. A., Banks, M. R., Thorpe, S., Sasieni, P., Bown, S. G., Novelli, M. R., Rabinovitch, P. S., and Lovat, L. B. (2010). Image cytometry accurately detects DNA ploidy abnormalities and predicts late relapse to high-grade dysplasia and adenocarcinoma in Barrett's oesophagus following photodynamic therapy. British journal of cancer *102*, 1608-1617.

Haroske, G., Baak, J. P., Danielsen, H., Giroud, F., Gschwendtner, A., Oberholzer, M., Reith, A., Spieler, P., and Bocking, A. (2001). Fourth updated ESACP consensus report on diagnostic DNA image cytometry. Analytical cellular pathology : the journal of the European Society for Analytical Cellular Pathology *23*, 89-95.
