# Supplementary figures and images for: BCL9L Dysfunction Impairs Caspase-2 Expression Permitting Aneuploidy Tolerance in Colorectal Cancer
Source: Cancer Cell. 2017 Jan 9;31(1):79–93. doi: 10.1016/j.ccell.2016.11.001 (PMC5225404; doi:10.1016/j.ccell.2016.11.001)

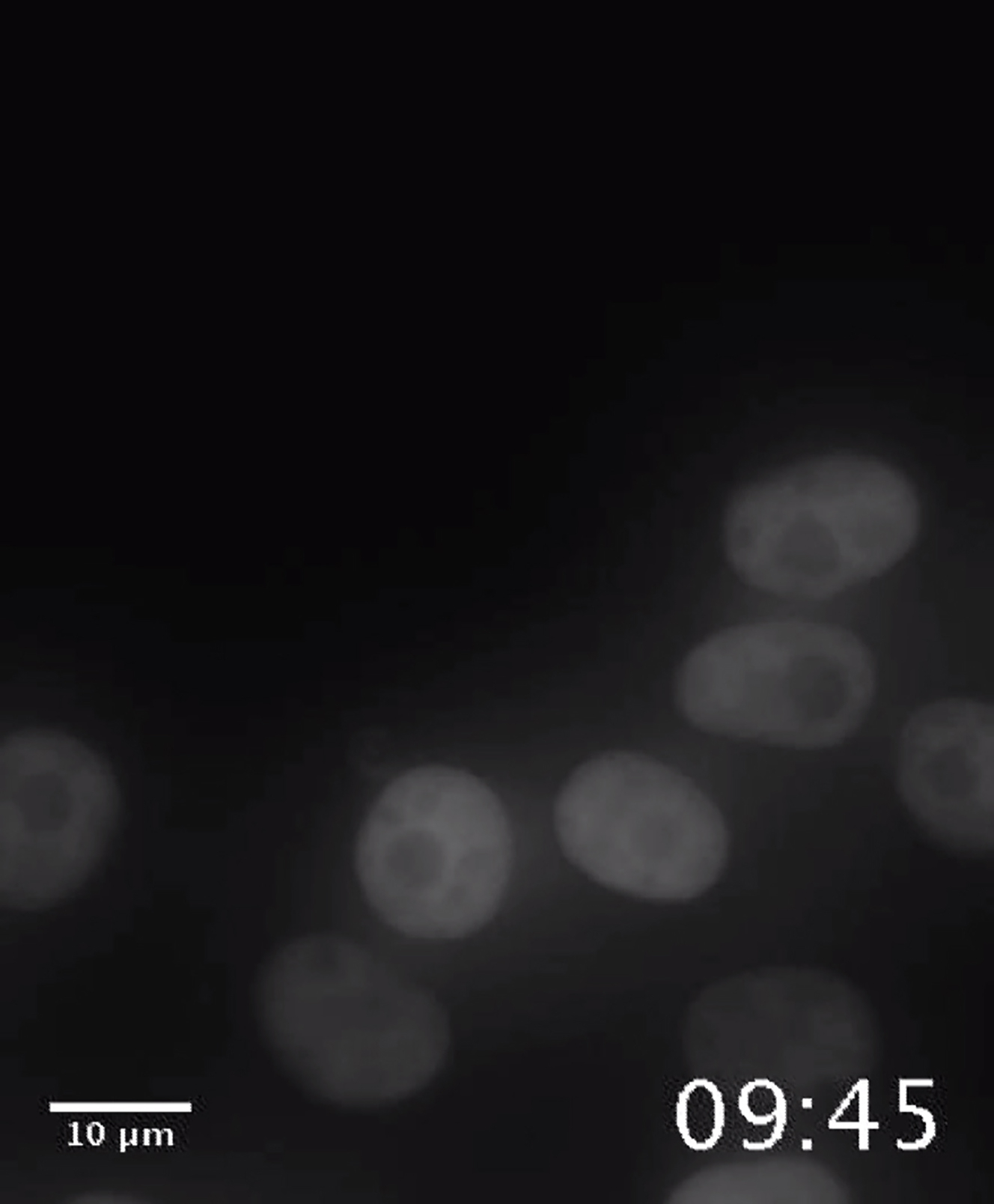

Supplement: Movie S1. Two Anaphases at 1 hr 33 min and 3 hr 33 min with Segregation Errors; Daughter Cells Arrest or Die. Related to Figure 2 [file mmc2.jpg]

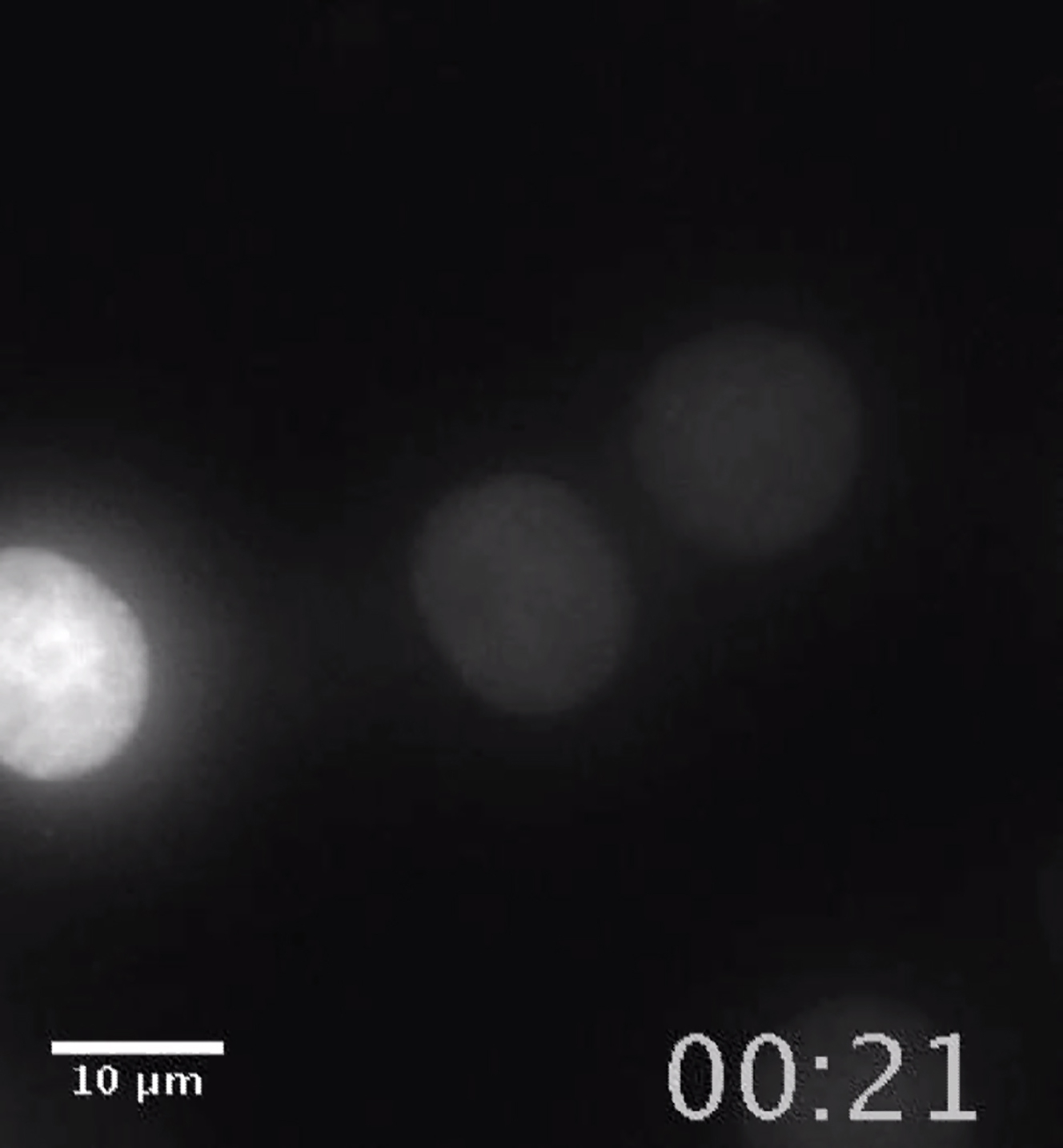

Supplement: Movie S2. Anaphase with Chromosomal Bridge at 3 hr 15 min; Daughter Cells Divide and Arrest. Related to Figure 2 [file mmc3.jpg]

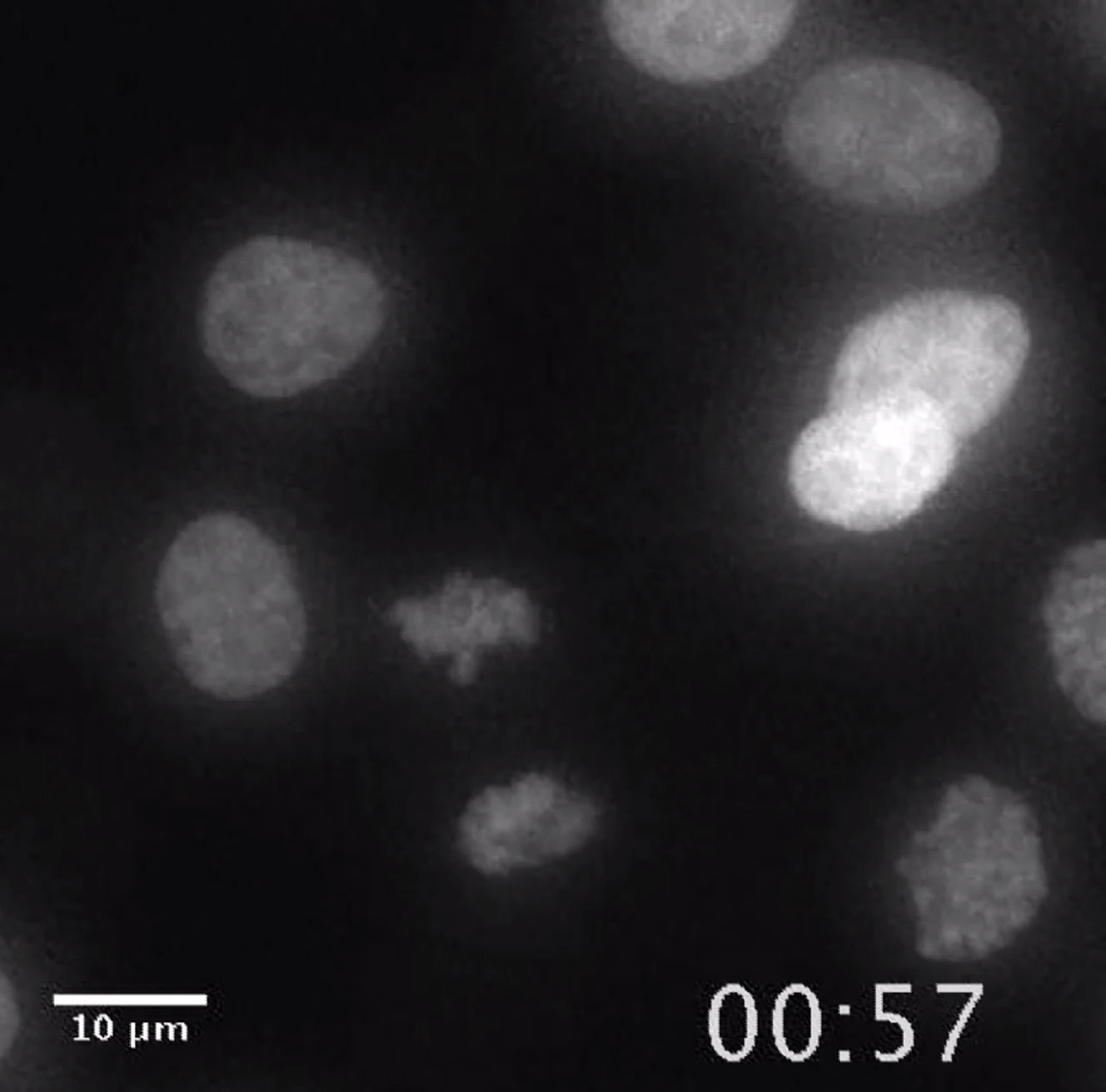

Supplement: Movie S3. Anaphase with Lagging Chromosome at 51 min; Both Daughter Cells Divide. Related to Figure 2 [file mmc4.jpg]

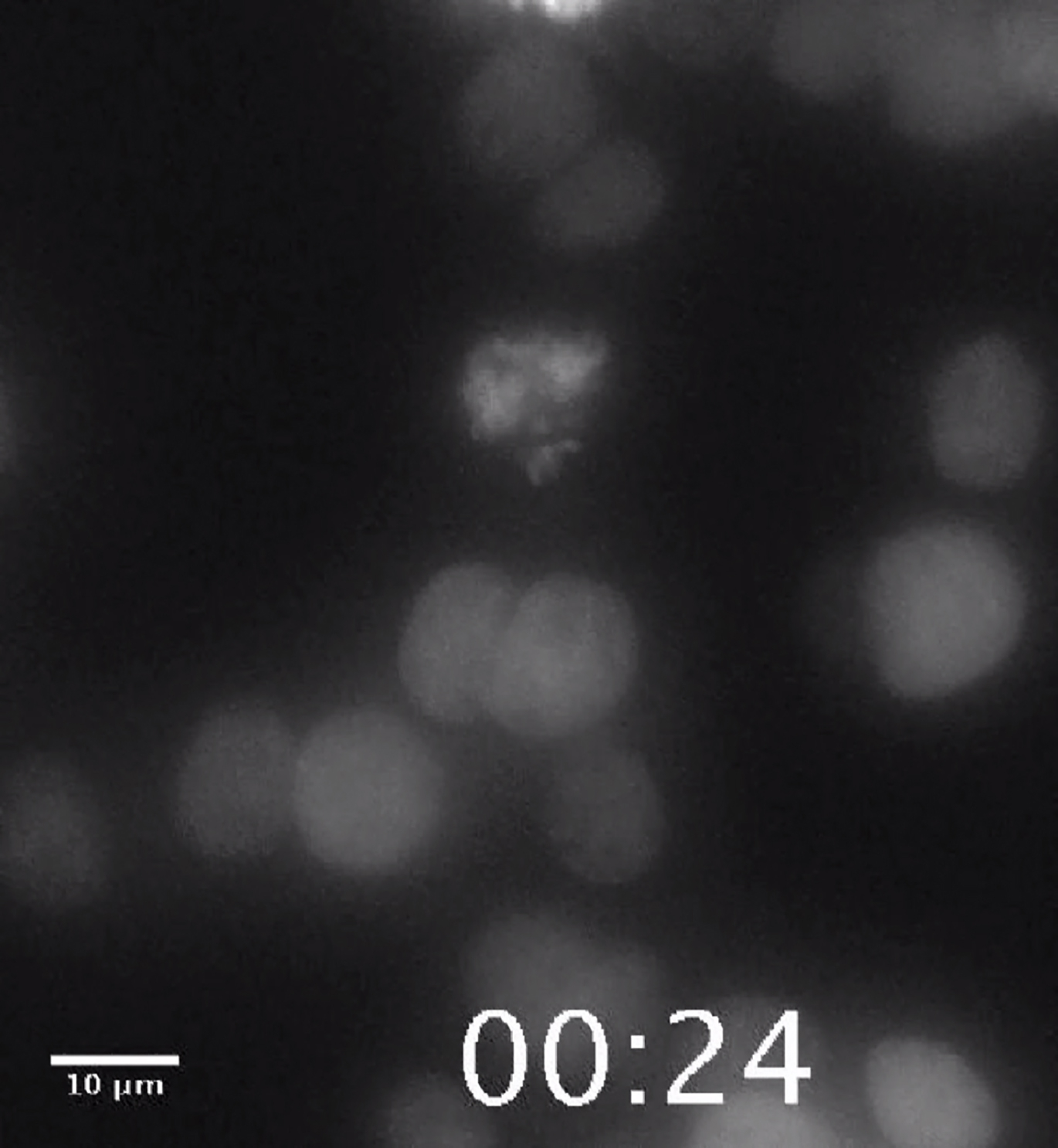

Supplement: Movie S4. Normal Mitosis at 42 min; Both Daughter Cells Divide. Related to Figure 2 [file mmc5.jpg]
