# Supplementary material for: BCL9L Dysfunction Impairs Caspase-2 Expression Permitting Aneuploidy Tolerance in Colorectal Cancer
Source: Cancer Cell. 2017 Jan 9;31(1):79–93. doi: 10.1016/j.ccell.2016.11.001 (PMC5225404; doi:10.1016/j.ccell.2016.11.001)
Supplement: Document S2. Article plus Supplemental Information [file mmc6.pdf]

Cancer Cell

Article

BCL9L Dysfunction Impairs Caspase-2 Expression Permitting Aneuploidy Tolerance in Colorectal Cancer

Graphical Abstract

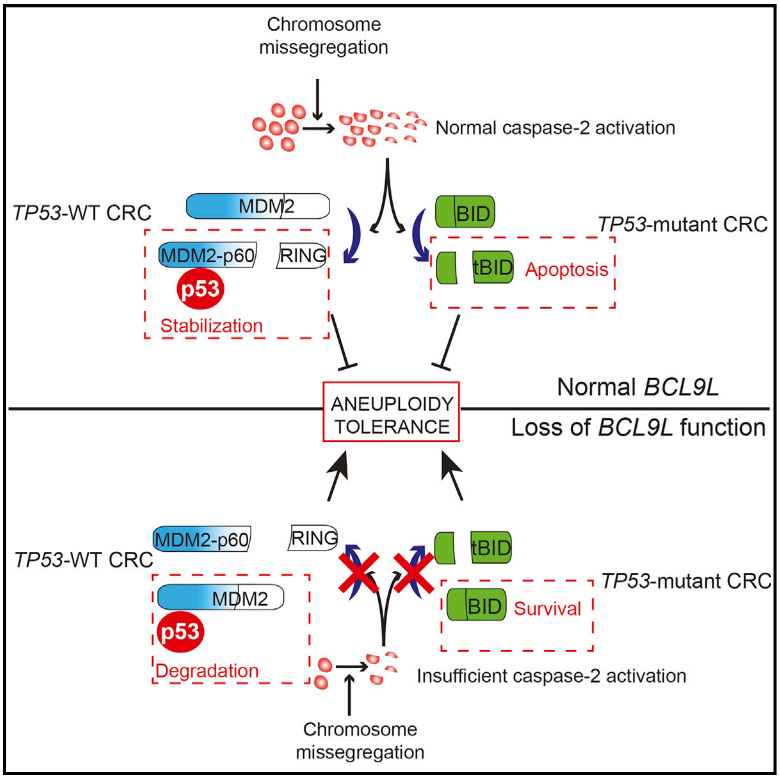

Highlights

- Loss-of-function alterations in *BCL9L* are frequent in aneuploid CRC
- *BCL9L* dysfunction drives aneuploidy tolerance by reducing levels of caspase-2
- Caspase-2 activation following aneuploidy results in MDM2 and BID cleavage
- p53 stabilization after chromosome missegregation is regulated by caspase-2

Authors

Carlos López-García,
Laurent Sansregret, Enric Domingo, ...,
Marco Novelli, Ian Tomlinson,
Charles Swanton

Correspondence

charles.swanton@crick.ac.uk

In Brief

López-García et al. find that *BCL9L* is often genetically inactivated in human colorectal cancers with chromosomal instability. *BCL9L* dysfunction promotes aneuploidy tolerance by reducing basal caspase-2 levels and preventing cleavage of MDM2 and BID independent of *TP53* mutation status.

López-García et al., 2017, Cancer Cell *31*, 79–93
January 9, 2017 © 2017 The Francis Crick Institute. Published by Elsevier Inc.
http://dx.doi.org/10.1016/j.ccell.2016.11.001

Cancer Cell
Article

CellPress

BCL9L Dysfunction Impairs Caspase-2 Expression Permitting Aneuploidy Tolerance in Colorectal Cancer

Carlos López-García,[1] Laurent Sansregret,[1] Enric Domingo,[5,6] Nicholas McGranahan,[1,7] Sebastijan Hobor,[1] Nicolai Juul Birkbak,[1,7] Stuart Horswell,[2] Eva Grönroos,[1] Francesco Favero,[1,8] Andrew J. Rowan,[1] Nicholas Matthews,[4] Sharmin Begum,[4] Benjamin Phillimore,[4] Rebecca Burrell,[1] Dahmane Oukrif,[9] Bradley Spencer-Dene,[3] Michal Kovac,[5] Gordon Stamp,[3] Aengus Stewart,[2] Havard Danielsen,[10] Marco Novelli,[9] Ian Tomlinson,[5] and Charles Swanton[1,7,11,*]
[1]Translational Cancer Therapeutics Laboratory
[2]Bioinformatics Science Technology Platform
[3]Experimental Histopathology Laboratory
[4]Advanced Sequencing Facility
The Francis Crick Institute, 1 Midland Road, London NW1 1AT, UK
[5]Oxford Centre for Cancer Gene Research, The Wellcome Trust Centre for Human Genetics, Roosevelt Drive, Oxford, OX3 7BN UK
[6]Department of Oncology, University of Oxford, Roosevelt Drive, Oxford OX3 7DQ, UK
[7]Translational Cancer Therapeutics Laboratory, University College London Cancer Institute, Paul O'Gorman Building, 72 Huntley Street, London WC2E 6DD, UK
[8]Cancer System Biology, Centre for Biological Sequence Analysis, Department of Systems Biology, Technical University of Denmark, Lyngby 2800, Denmark
[9]Research Department of Pathology, University College London Medical School, University Street, London WC1E 6JJ, UK
[10]Institute for Cancer Genetics and Informatics, Norwegian Radium Hospital, Oslo University Hospital, Ullernchausseen 70, 0379 Oslo, Norway
[11]Lead Contact
*Correspondence: charles.swanton@crick.ac.uk
http://dx.doi.org/10.1016/j.ccell.2016.11.001

SUMMARY

Chromosomal instability (CIN) contributes to cancer evolution, intratumor heterogeneity, and drug resistance. CIN is driven by chromosome segregation errors and a tolerance phenotype that permits the propagation of aneuploid genomes. Through genomic analysis of colorectal cancers and cell lines, we find frequent loss of heterozygosity and mutations in *BCL9L* in aneuploid tumors. *BCL9L* deficiency promoted tolerance of chromosome missegregation events, propagation of aneuploidy, and genetic heterogeneity in xenograft models likely through modulation of Wnt signaling. We find that *BCL9L* dysfunction contributes to aneuploidy tolerance in both *TP53*-WT and mutant cells by reducing basal caspase-2 levels and preventing cleavage of MDM2 and BID. Efforts to exploit aneuploidy tolerance mechanisms and the BCL9L/caspase-2/BID axis may limit cancer diversity and evolution.

INTRODUCTION

Emerging evidence supports the influence of intratumor heterogeneity on patient outcome and drug response (Ding et al., 2012; Landau et al., 2013; Szerlip et al., 2012). Genomic instability is frequently observed in cancer (Lengauer et al., 1998; Negrini et al., 2010), driving intercellular variation and subsequent intratumor heterogeneity, providing the substrate for selection and tumor evolution (Boutros et al., 2015; Desmedt et al., 2015; Yates et al., 2015).

Chromosomal instability (CIN) is a form of genome instability characterized by the ongoing disorder of chromosome number and/or structure. Numerical CIN occurs after whole chromosome missegregation due to mitotic defects (Bakhoum et al.,

Significance

This comprehensive genomic analysis of aneuploid colorectal cancer identified frequent mutations and deletions of *BCL9L* leading to caspase-2 dysfunction and the tolerance of chromosome missegregation, which operates independently of *TP53* status. These data support the existence of parallel pathways complementing *TP53* dysfunction in the tolerance of aneuploidy and the central role for caspase-2 in the stabilization of p53 following chromosome missegregation events.

CrossMark

This is an open access article under the CC BY license (http://creativecommons.org/licenses/by/4.0/).

Table 1. Tumor DNA Computational Purity, DNA Indices, and Centromeric FISH Analysis of a Cohort of 17 MSS Colorectal Adenocarcinomas

			Chr2			Chr15			
			Mode (%MCD)[a]			Mode (%MCD)[a]			
Sample	Purity	DNA Index	Normal	Tumor	p Value[b]	Normal	Tumor	p Value[b]	Ploidy[c]
389	0.42	1.58	2 (41)	1 (60)	0.006	2 (39)	1 (61)	5×10^{-4}	A
363	0.69	2.23	2 (39)	3 (74)	5×10^{-4}	2 (32)	2 (65)	5×10^{-4}	A
391	0.48	1.59	2 (37)	2 (60)	5×10^{-4}	2 (38)	1 (64)	0.005	A
377	0.40	1.5	2 (42)	3 (60)	5×10^{-4}	2 (45)	3 (57)	5×10^{-4}	A
361	0.8	1.27	2 (43)	1 (64)	5×10^{-4}	2 (30)	2 (54)	0.01	A
379	0.36	1.63	2 (39)	4 (74)	5×10^{-4}	2 (31)	2 (53)	0.03	A
367	0.21	1.42	2 (36)	3 (56)	5×10^{-4}	2 (41)	2 (56)	0.1	A
369	0.48	1.61	2 (37)	3 (69)	5×10^{-4}	2 (38)	3 (55)	5×10^{-4}	A
365	0.18	1.49	2 (33)	2 (53)	0.002	2 (39)	3 (53)	5×10^{-4}	A
395	0.42	1	2 (47)	2 (55)	0.12	2 (43)	1 (42)	5×10^{-4}	A
375	0.35	1	2 (37)	2 (33)	0.14	2 (34)	2 (32)	0.25	D
397	0.49	1	2 (36)	2 (41)	0.18	2 (37)	2 (32)	0.25	D
385	0.56	1	2 (41)	2 (34)	0.22	2 (35)	2 (29)	0.22	D
381	0.63	1	2 (37)	2 (37)	0.4	2 (30)	2 (24)	0.05	D
383	0.47	1	2 (39)	2 (48)	0.5	2 (32)	2 (35)	0.22	D
373	0.65	1	2 (47)	2 (51)	0.7	2 (48)	2 (55)	0.19	D
371	0.35	1	2 (39)	2 (33)	0.12	2 (45)	2 (35)	0.19	D

See also Figures S1 and S2; Table S1.
[a]Percentage modal centromeric deviation.
[b]Normal-tumor comparison (two-tailed Fisher's exact test and Monte Carlo simulations).
[c]A = aneuploid; D = diploid.

2009a, 2009b; Cahill et al., 1998; Sotillo et al., 2007) and results in an aberrant chromosome number, known as aneuploidy. Structural CIN results in the disordered integrity of parts of chromosomes. Both types of CIN are interconnected: missegregated chromosomes are exposed to mitotic stress that generates structural CIN (Crasta et al., 2012; Janssen et al., 2011) while changes in chromosome structure render them susceptible to missegregation (Burrell et al., 2013; Chan et al., 2007; Kawabata et al., 2011; Pampalona et al., 2010).

Since chromosome segregation errors are poorly tolerated by diploid cells (Dewhurst et al., 2014; Iwanaga et al., 2007; Thompson and Compton, 2008, 2010), survival mechanisms, termed aneuploidy tolerance, are crucial for the propagation of aneuploidy in tumors. Mutations in *TP53* (Grim et al., 2012; Li et al., 2010; Thompson and Compton, 2010) and buffering of protein changes due to aneuploidy (Stingele et al., 2013; Torres et al., 2010) have been proposed as candidate mechanisms of aneuploidy tolerance. Due to the potential clinical benefit of limiting CIN in tumors, further efforts to elucidate these survival mechanisms might contribute to limiting this driver of heterogeneity.

Colorectal cancer (CRC) can be broadly divided into microsatellite-instability high (MSI, 20%) and microsatellite-stable tumors (MSS, 80%). MSI CRC tumors remain near diploid, whereas MSS tumors develop a wide range of aneuploid karyotypes and CIN (Bogaert and Prenen, 2014; Cancer Genome Atlas Network, 2012; Mouradov et al., 2013; Rowan et al., 2005). *TP53* mutations occur frequently in aneuploid tumors (Cianchi et al., 1999; Rowan et al., 2005; Tang et al., 2004); however, next-generation sequencing efforts have not specifically explored the somatic mutational landscapes of aneuploid versus diploid MSS CRC tumors to identify determinants of CIN.

In this study, we aimed to identify somatic mutations enriched in aneuploid CRC and to elucidate the potential role of these mutations in the development of CIN in CRC.

RESULTS

Somatic Mutation Analysis in CIN Colorectal Cancer Genomes

We selected a cohort of 17 MSS colorectal adenocarcinomas and eight MSS aneuploid cell lines (see Table S1 for clinical features) for whole-exome sequencing. Ploidy status was deduced by calculation of the DNA index (DI) using DNA image cytometry data obtained from nuclei isolated from paraffin-embedded specimens (Table 1 and Figure S1). DI was calculated as the ratio between the mode of the relative DNA content of observable tumor nuclei peaks and a diploid control consisting of nuclei from infiltrated fibroblasts, endothelial cells, and immune cells. To validate the DNA image cytometry results, we performed centromeric fluorescence in situ hybridization (FISH) for chromosomes 2 and 15 (Table 1), since these chromosomes are not frequently subject to whole chromosome gains or losses in CRC (Orsetti et al., 2014). The overall distribution of centromeric signals in tumor cells was compared with the normal adjacent tissue (Table 1; Figures S2A and S2B). By convention, a tumor was classified as aneuploid when an aneuploid peak was detected by DNA image cytometry (DI between 1.1 and 1.89 or greater than 2.1) or when significant changes in the distribution

Table 2. Ploidy and Karyotype Data for the Eight Exome-Sequenced CRC Cell Lines

Cell Line	Age	Dukes Stage	MCN[a]	Ploidy
T84	NR[b]	NR[b]	56	hyperdiploid
SW948	72	C3	67	hypotriploid
SW403	81	C3	68	triploid
SK-CO-1	51	NR[b]	75	hypertriploid
SW1463	65	C1	NR	hypertetraploid
NCI-H508	66	NR[b]	102	hypotriploid
LS123	55	B	63	hypotriploid
HT55	NR[b]	NR[b]	72	hypertriploid

[a]Modal chromosome number.
[b]Not reported.

of centromeric signals were detected for one of the chromosomes tested. A tumor was classified as diploid when no aneuploid populations were detected by DNA image cytometry (only one peak with DI = 1) and no significant changes were detected in the distribution of centromeric signals for the two chromosomes tested. We detected modal chromosome signals different from 2 for at least one chromosome in samples with aneuploid peaks (Table 1). For the MSS cell lines, ploidy status was obtained from published karyotyping and SNP array analysis (Table 2, Gaasenbeek et al., 2006; Lee et al., 2011).

Aneuploid CRC is strongly associated with CIN defined by cell-to-cell variation of centromeric signals and surrogate parameters that measure karyotypic complexity in cancer genomes such as the weighted genome instability index (wGII), which assesses the fraction of the genome with alterations (Burrell et al., 2013; Chin et al., 2007). We observed that both modal centromere deviation (MCD) for chromosomes 2 and 15 and the wGII were significantly higher in aneuploid tumors (Figures S2C and S2D). In samples with DI = 1, the modal centromere signal was 2 in all cases except tumor 395, which showed significant alterations in the overall distribution of chromosome 15 signals and was therefore classified as aneuploid (Table 1 and Figure S2E). This classification was also supported by a high wGII (0.41). No significant differences were observed in tumor sample purity between aneuploid and diploid tumors (Table 1 and Figure S2F). Taken together, ten MSS tumors were classified as aneuploid and seven as diploid (Table 1).

To attempt to identify aneuploidy-specific mutations, we performed exome sequencing on DNA from tumors, normal adjacent tissue, and cell lines (Table S2), and mutation calling of tumor somatic variants was performed by filtering germline variants identified in normal adjacent colon. Manual curation of variant calls and validation by Sanger sequencing revealed a list of 32 genes specifically mutated in aneuploid samples (Figure 1A and Table S3). Notably, known CRC drivers (*APC*, *FBXW7*, and *KRAS*) did not segregate according to tumor ploidy status. As expected, however, *TP53* mutations were significantly enriched in aneuploid tumors (Figure 1A, 13/18 aneuploid tumors and cell lines, 0/7 diploid samples; p = 0.001, Fisher's exact test). No somatic mutations were mutually exclusive with *TP53* in this discovery cohort.

BCL9L was the only gene for which all mutations found were clearly inactivating, with one nonsense mutation Q713* in tumor 379, one nonsense mutation R716* in the cell line SW1463, and one splice-site variant (exon 5 + 1 G > A) in the tumor sample 363. We also found loss of heterozygosity (LOH) at the *BCL9L* locus in two aneuploid tumors (391 and 389) and one cell line (SK-CO-1). R716* was observed in two of four alleles of the aneuploid cell line SW1463. Q713* and the splice-site mutation were observed in two of three alleles of tumor samples 379 and 363, respectively, which suggests that *BCL9L* mutations occurred early, prior to chromosome duplication.

Next, we performed two functional RNAi screens probing phenotypes relevant to chromosome segregation errors and their tolerance. We used the diploid cell line HCT-116 due to its low level of constitutive chromosome segregation errors relative to CIN CRC cell lines and its poor tolerance of drug-induced segregation errors (Gascoigne and Taylor., 2008; Lengauer et al., 1997; Thompson and Compton, 2010). In the first RNAi screen we silenced each of the 32 genes mutated in aneuploid samples and examined the consequence upon chromosome segregation error frequency as previously described (Burrell et al., 2013), with no significant results. Second, we performed a screen to detect tolerance of chromosome segregation errors. Chromosome missegregation induces p53-mediated cell cycle arrest in the next G1 phase, often followed by apoptosis, thereby preventing propagation of aneuploid progeny (Figure S3A; see also Hinchcliffe et al., 2016). Chromosome missegregation can be artificially induced in HCT-116 cells with reversine, an Mps1 inhibitor that impairs the spindle assembly checkpoint resulting in chromosomal non-disjunction and missegregation. Consistent with results by Jemaa et al. (2012), we found that reversine treatment induces subsequent arrest or cell death (Figure S3A). We depleted all aneuploid-specific genes (Figure 1A) individually in HCT-116 cells with small interfering RNA (siRNA) pools from Dharmacon in the presence or absence of 250 nM reversine, a concentration that does not inhibit Aurora kinase B (Santaguida et al., 2010). Silencing of *TP53*, *RABGAP1*, *BCL9L*, *HDLBP*, and *ZFHX3* induced reversine tolerance (Figure 1A, right). We considered a gene validated when at least three of four individual oligonucleotides (components of the pool of four) showed the same effect as the pool. *TP53*, *BCL9L*, and *ZFHX3* were validated following these criteria (Figure S3B). Experiments with distinct Qiagen siRNA pools also showed a similar result for *TP53*, *BCL9L*, and *ZFHX3* (Figure S3B).

Efficient depletion of the BCL9L protein was observed for all four single oligonucleotides (Figure S3C); however, we discarded the BCL9L oligonucleotide 4 due to high cellular toxicity. Finally, expression of a BCL9L-EGFP construct lacking the 3′ UTR region reverted the survival phenotype in reversine when an siRNA targeting 3′ UTR was transfected, further supporting that aneuploidy tolerance observed with various siRNA duplexes is due to on-target silencing of *BCL9L* (Figure S3D). *BCL9L* silencing also increased cell viability following treatment with 200 nM aphidicolin (Figure S3E), which causes replication stress and induces segregation errors of structurally unstable chromosomes (Burrell et al., 2013). No tolerance effect was observed when HCT-116 cells were treated with doxorubicin, suggesting that silencing of *BCL9L* does not result in general resistance to cytotoxics causing DNA damage (Figure S3F).

Given the aneuploid-specific pattern of LOH and truncating events of *BCL9L*, the second most commonly truncated gene in aneuploid CRC after *TP53* in our discovery cohort (Figure 1A

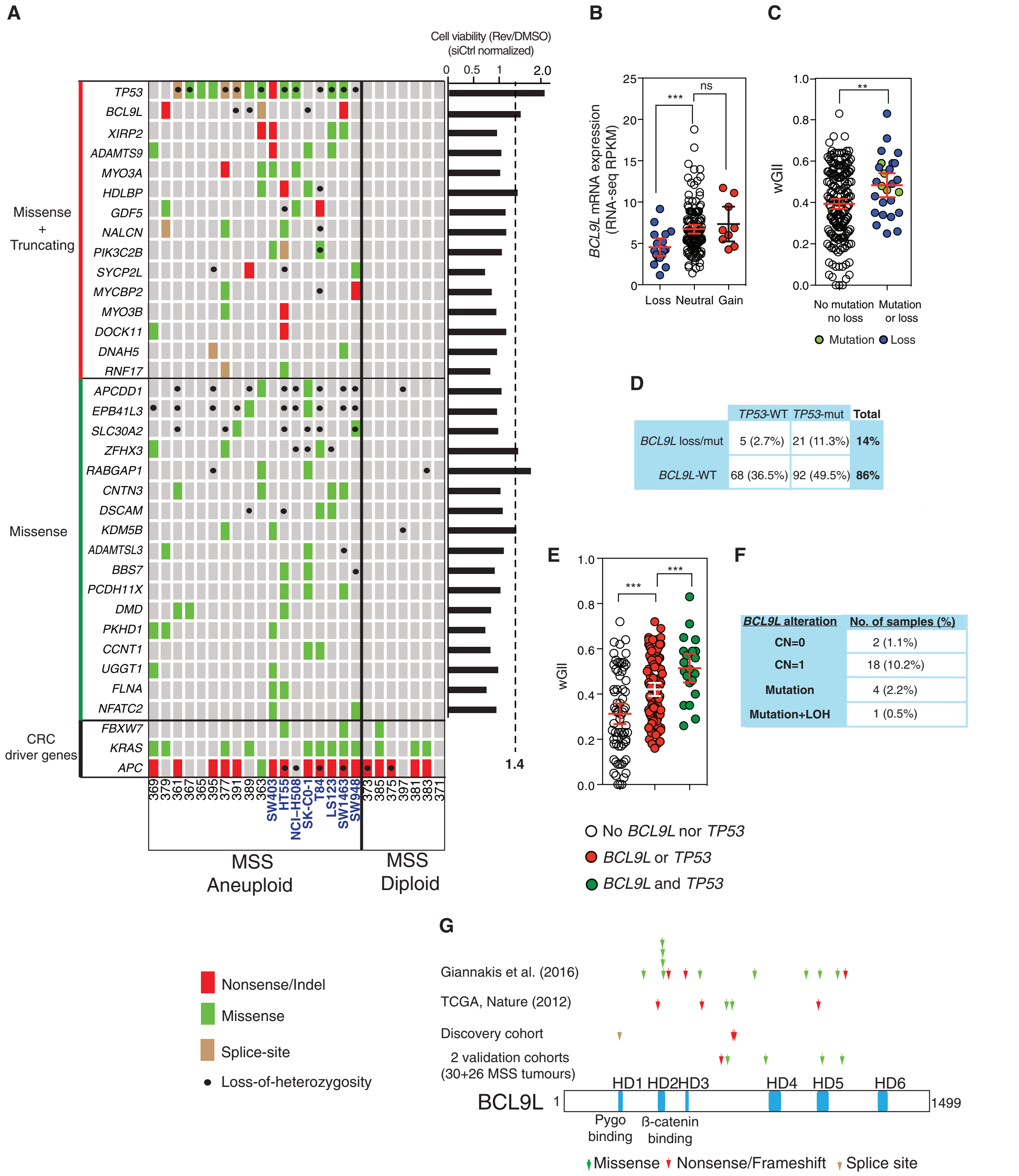

Figure 1. Genomic Analysis of Aneuploid CRC and Cell Lines

(A) Non-synonymous somatic mutations and loss of heterozygosity in a discovery cohort of 17 MSS CRC tumors and eight cell lines (in blue). Genes mutated in aneuploid samples are shown separated in two categories (truncating + missense or missense only). Common CRC driver genes are shown at the bottom. Right-hand graph presents the results of the reversine tolerance screen. Each value was normalized to siControl (siCtrl) treated cells, and genes whose knockdown provided a $\geq$1.4-fold increase in viability were considered for further analysis.

(B) Correlation between *BCL9L* copy number and mRNA level in MSS CRC in the TCGA dataset. Loss (CN = 0 or 1), neutral (CN = 2), and gain (CN $\geq$ 3). RPKM, reads per kilobase of transcript per million mapped reads; p values were calculated by unpaired Student's t test.

(C) wGII in MSS CRC TCGA samples with *BCL9L* alterations and wild-type *BCL9L* (p values calculated by unpaired Student's t test).

(D) Co-occurrence and mutual exclusivity of *BCL9L* alterations and *TP53* mutations in TCGA MSS CRC (percentage of all MSS samples).

(legend continued on next page)

and Table S3), and its putative aneuploidy tolerance function in the siRNA screen, we investigated *BCL9L* somatic events in independent cohorts. We analyzed data from 186 MSS CRCs available from The Cancer Genome Atlas (TCGA) (Cancer Genome Atlas Network, 2012). *ZFHX3* was not investigated further as mutations in this gene were not enriched in aneuploid CRC in validation cohorts.

We confirmed that samples with somatic copy-number loss of *BCL9L* had significantly lower gene expression compared with samples with no alterations in *BCL9L* (Figure 1B). Using the wGII score as a surrogate of chromosomal instability and aneuploidy in CRC as previously described (Chin et al., 2007; Lee et al., 2011), we observed significantly higher wGII scores in tumors harboring *BCL9L* mutation or copy-number loss compared with tumors with no alteration in *BCL9L* (Figure 1C). This relationship remained significant when controlling for the higher probability of gene loss in high-wGII tumors (Figure S3G, $p = 0.03$).

The majority of *BCL9L* deletions and mutations co-occurred with *TP53* mutations (21/26 tumors with co-occurrence, Figure 1D). In a similar computational permutation analysis as performed above (Figures 1C and S3G), samples with co-occurring *BCL9L* and *TP53* alterations displayed higher wGII scores compared with those with mutually exclusive alterations (Figure 1E, $p = 0.007$), suggesting that these two genes might cooperate as aneuploidy suppressors in CRC.

Comprehensive genomic analysis from the same colorectal TCGA cohort enabled us to infer the genotype of *BCL9L* alterations in MSS CRC (Figure 1F). In total, *BCL9L* mutations and/or deletions occurred in 14% of MSS CRC (26/186, 5 mutations and 21 deletions), the majority of which (23/26) retained a wild-type (WT) copy of *BCL9L* while biallelic alterations of *BCL9L* occurred in only three samples. Taken together, these results suggest a haploinsufficient model of tumor suppression for *BCL9L*.

Finally, we evaluated the pattern of *BCL9L* non-synonymous mutations in MSS CRC across the BCL9L protein (Figure 1G). We compiled all *BCL9L* mutation data from the discovery cohort in Figure 1A, the TCGA MSS CRC cohort, a large cohort of 438 MSS CRC published by Giannakis et al. (2016) together with two additional validation cohorts of MSS CRC tumors sequenced by Ion Torrent targeted sequencing (Figure S3H). The 27 *BCL9L* somatic mutations identified were scattered across the gene with one cluster of four missense mutations mapping to two adjacent residues within the β-catenin binding HD2 domain (R409W and S410F). Thirty-seven percent (10/27) of the somatic mutations were truncating events (nonsense, splice-site, and indel mutations) whereas 17 of 27 were missense mutations (see Table S4 for functional impact). This characteristic profile of scattered mutations with >20% of inactivating/truncating mutations is consistent with the tumor-suppressor pattern proposed by Vogelstein et al. (2013). Consistent with these data, comprehensive computational analysis has classified *BCL9L* as a candidate driver gene in a pan-cancer analysis (Tamborero et al., 2013) and as a significantly mutated driver gene in MSS CRC (Giannakis et al., 2016).

Loss of *BCL9L* Drives Tolerance to Segregation Errors and Aneuploidy

The results shown above prompted us to carry out a more detailed study of the role of *BCL9L* dysfunction in aneuploidy tolerance. The diploid cell line HCT-116 expresses high levels of BCL9L, and siRNA transfection efficiently depleted BCL9L protein and mRNA (Figure 2A). *BCL9L* silencing increased the number of metabolically active cells, total cell number, bromodeoxyuridine (BrdU)-incorporating cells, and colony-forming efficiency in reversine-treated HCT-116 cells (Figures 2B–2E) and reduced reversine-induced apoptosis (Figure 2F). In the absence of reversine, *BCL9L* silencing did not induce any significant changes in cell proliferation or apoptosis, nor did it affect the rate of constitutive segregation errors (Figure S4A). BCL9L knockdown also induced reversine tolerance in a panel of near-diploid colorectal cell lines that express BCL9L (Gaasenbeek et al., 2006; Lee et al., 2011; Figures S4B–S4D). In contrast, survival of *BCL9L* mutant and/or non-expressing cells (LS-174T and RKO) in reversine was not improved after *BCL9L* silencing, suggesting on-target specificity of the BCL9L siRNAs (Figures S4B–S4D; see also COSMIC database at cancer.sanger.ac.uk).

Next, we examined the fate of daughter cells arising from error-free mitoses or mitoses with naturally occurring segregation errors. For this we used HCT-116 cells expressing H2B-RFP to visualize chromosomes. Following control siRNA transfection, the majority of daughter cells that had undergone a chromosome segregation error did not divide again within 48 hr (Figures 2G and S5A, and Movies S1, S2, S3, and S4 for examples of endogenous segregation errors). Longer-term observation of cells that had undergone a chromosome segregation error revealed that 24.5% of arrested cells died between 48 and 72 hr after mitosis (Figure S5B). In contrast, the majority of daughter cells entered a second mitosis following silencing of *BCL9L* or *TP53*, whether an endogenous segregation error occurred or not (Figures 2G and S5A). Similar results were found through live-cell microscopy of three additional cell lines (Figure S5C). These results suggest that *BCL9L* dysfunction promotes survival following chromosome segregation errors by a mechanism that may not be unique to Mps1 inhibition but a more general mechanism that also applies to endogenous chromosome segregation errors.

We generated HCT-116 cells with partial depletion of BCL9L (Figure S5D) using a lentiviral small hairpin RNA (shRNA) vector to study the long-term consequences of *BCL9L* silencing. Treatment with 125 nM reversine for 15 days revealed an increase in colony-forming efficiency in the shBCL9L cells relative to shControl (shCtrl) cells (Figure 2H). To study whether BCL9L depletion promotes the propagation of aneuploid cells, we treated cells with 125 nM reversine for 15 days followed by a 2-week recovery in drug-free medium, and performed centromeric FISH analysis with centromeric probes for four chromosomes (Figure 2I). In untreated cells, *BCL9L* silencing produced a small but significant increase in the modal centromeric deviation when compared with shCtrl for chromosomes 2 and 8. In

(E) wGII in samples with mutually exclusive and co-occurring *BCL9L* and *TP53* somatic alterations (MSS CSC TCGA; p values calculated by unpaired Student's t test).

(F) Allelic pattern of *BCL9L* alterations in TCGA MSS CRC (percentage of all MSS samples).

(G) Mapping of non-synonymous mutations across the BCL9L protein identified in four different cohorts.

Error bars denote 95% confidence interval. ns, not significant. $^{**}p < 0.01$, $^{***}p < 0.001$. See also Figure S3 and Tables S2–S4.

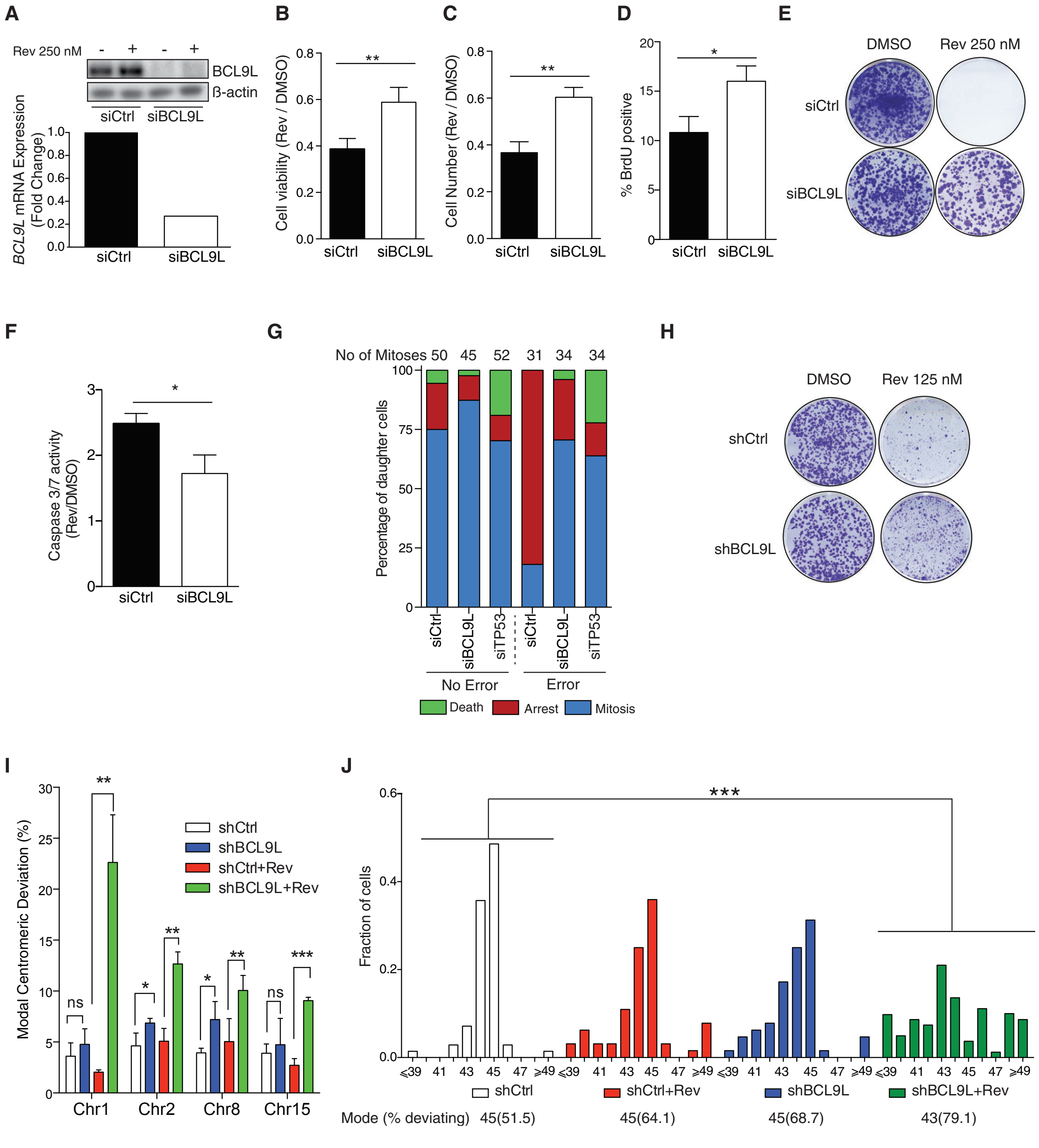

Figure 2. BCL9L Knockdown Confers Aneuploidy Tolerance in HCT-116 Cells

(A) siRNA knockdown of BCL9L protein (top) and mRNA (bottom) in HCT-116 (representative experiments shown, Qiagen siRNA pool).

(B–D) Impact of 250 nM reversine treatment on cell viability (B), cell number (C), and fraction of BrdU-incorporating cells (D) following control or BCL9L siRNA transfection (72 hr, n = 3; p values calculated by paired Student's t test).

(E) Colony-forming efficiency of HCT-116 after siRNA transfection and 250 nM reversine treatment.

(F) Fold change induction of Caspase-3/7 enzymatic activity following 250 nM reversine treatment in HCT-116 transfected control siRNA or after BCL9L knockdown (72 hr, n = 3, p value calculated by paired Student's t test).

(G) Live-cell imaging analysis of post-mitotic daughter cell fate after a normal mitosis or mitosis with endogenous segregation errors. Daughter cells were tracked for at least 48 hr. Arrest was defined as the absence of cell division within 48 hr post mitosis. Death was defined as visible nuclear collapse.

(H) Effect of stable lentiviral BCL9L knockdown on colony-forming efficiency of HCT-116 treated with 125 nM reversine for 15 days and recovered for additional 15 days.

(I) Modal centromeric deviation (%) in the number of FISH signals (CEP 1, 2, 8, and 15) in reversine-treated and untreated HCT-116 cells with lentiviral stable BCL9L knockdown (n = 3, 400–500 nuclei scored per experiment). Cells were treated as in (H).

(legend continued on next page)

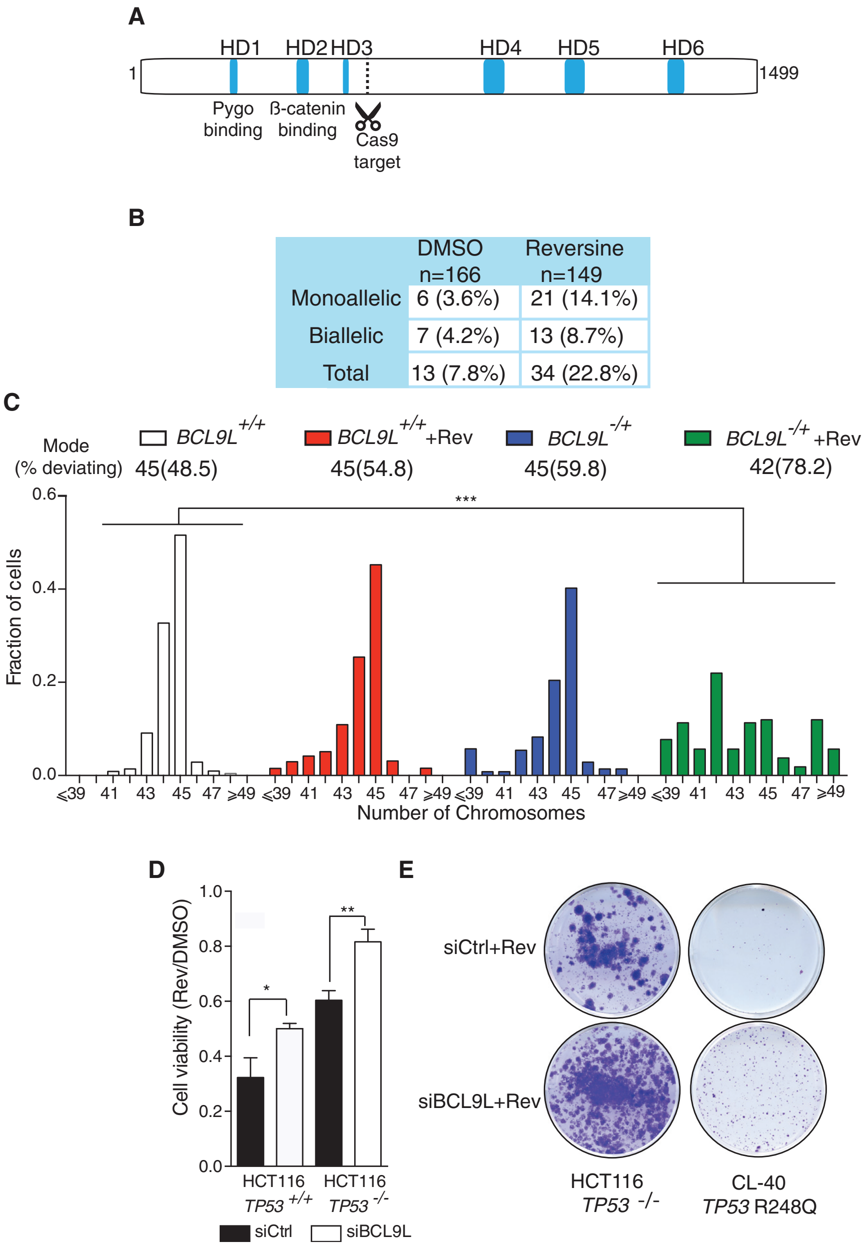

Figure 3. Heterozygous Truncation of *BCL9L* Drives Aneuploidy Tolerance in HCT-116 Cells

(A) Mapping of the CRISPR protospacer site on the BCL9L protein. Guide RNA targets nucleotides 2,542–2,561 (*BCL9L* cDNA sequence GenBank: NM_182557).

(B) Genotyping results of *BCL9L* CRISPR clones selected in 125 nM reversine for 15 days (percentage of all isolated clones).

(C) Karyotypic analysis of an isolated HCT-116 clone bearing a heterozygous 5-bp deletion C-terminal to the HD3 domain in *BCL9L* (p.Glu530fs) (70–100 cells analyzed, modal number of chromosomes and percent deviating from the mode shown above the graph; p value calculated by two-sided Wilcoxon rank-sum test).

(D) Effect of BCL9L depletion on cell viability of isogenic *TP53*-WT and null HCT-116 cells (72 hr, n = 3; p value calculated by paired Student's t test). Cell viability was measured by CellTiter-Blue (Promega).

(E) Colony-formation assay in CRC *TP53*-mutant cells (*TP53*-null HCT-116 and CL-40).

Error bars denote SD. *p < 0.05, **p < 0.01, ***p < 0.001. See also Figure S5.

shBCL9L cells pre-treated with reversine, this increase was significant for all four probes (Figure 2I). Total chromosome counts carried out on metaphase spreads derived from the same cells supported the development of aneuploidy in BCL9L-depleted cells treated with reversine (Figure 2J). We did not detect structurally aberrant chromosomes in metaphase spreads. There was no evidence of cytokinesis failure resulting in tetraploidization in BCL9L-depleted cells treated with reversine (Figure S5E).

Next, we engineered *BCL9L* truncating mutations in HCT-116 cells similar to those observed in CRC using CRISPR/Cas9. Since most truncating *BCL9L* mutations preserve HD1, HD2, and HD3 domains (Figure 1G), we designed a guide RNA targeting *BCL9L* C-terminal to the HD3 domain (Figure 3A). Sanger sequencing of clones selected in 125 nM reversine for 2 weeks showed a 2.9-fold enrichment in *BCL9L* mutant clones selected in reversine when compared with untreated colonies (Figure 3B). Both monoallelic and biallelic *BCL9L* truncations appeared to be selected by reversine treatment (Figure 3B). Karyotypic analysis of metaphase spreads of HCT-116 with a heterozygous 5-bp deletion C-terminal to the HD3 domain generated by CRISPR/Cas9 (reduction of BCL9L protein shown in Figure S5F) showed an increase in aneuploidy in reversine-treated *BCL9L*$^{-/+}$ cells in comparison with the WT control (Figure 3C). Taken together, these results with *BCL9L* mutant cell lines and our genomic analysis support a role for *BCL9L* haploinsufficiency conferring aneuploidy tolerance.

The majority of CRCs with *BCL9L* alterations also harbor *TP53* mutations and this co-occurrence seems to coincide with higher wGII scores in tumors (Figures 1D and 1E). Silencing of *BCL9L* in HCT-116 *TP53*-null cells also increased the fraction of surviving cells after 3 days of reversine treatment (Figure 3D) and the

(J) Karyotypic analysis following stable lentiviral BCL9L knockdown and reversine treatment. Metaphases were stained with a pan-centromeric probe (70–100 cells analyzed, modal number of chromosomes and percentage deviating from the mode shown at the top; p values calculated by two-sided Wilcoxon rank-sum test).

Error bars denote SD. ns, not significant. *p < 0.05, **p < 0.01, ***p < 0.001. See also Figures S4 and S5.

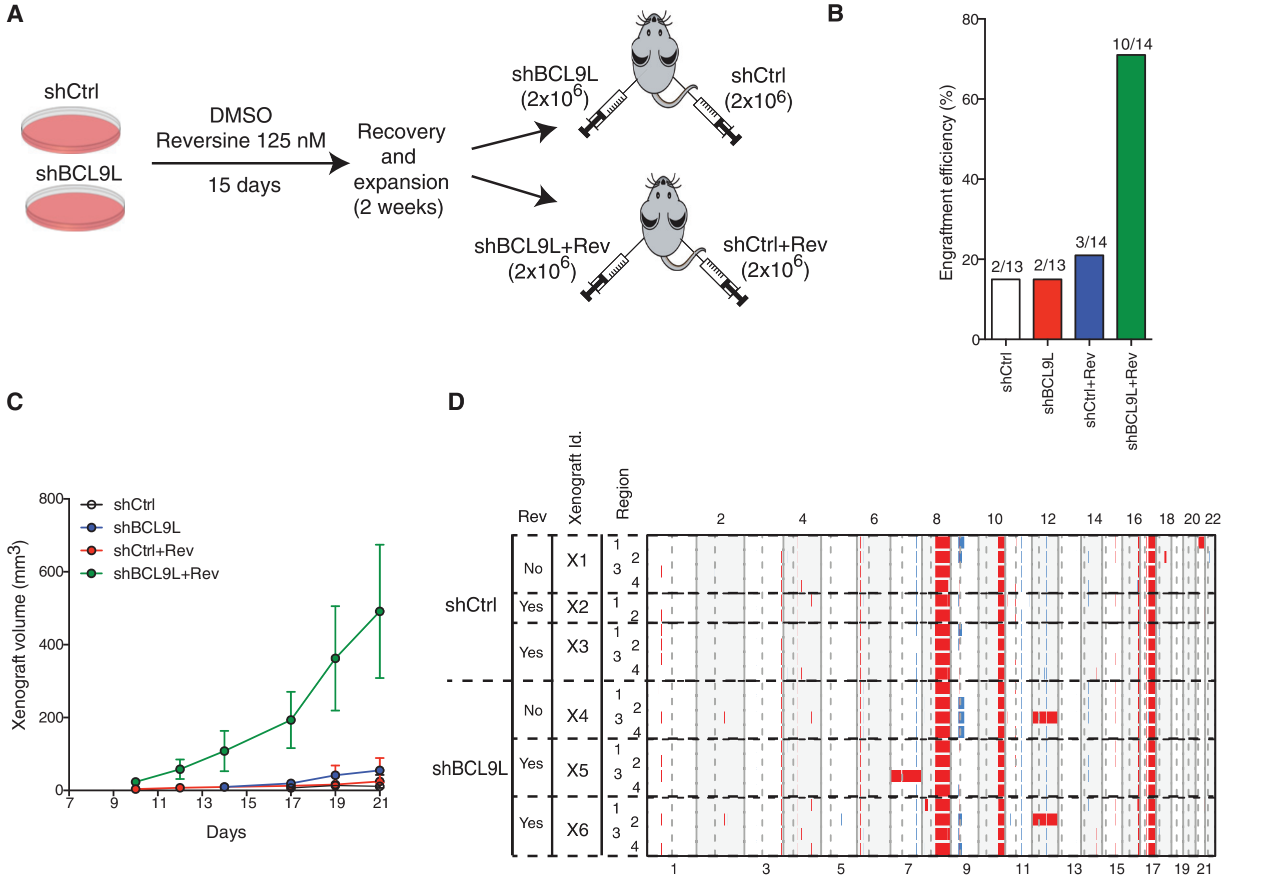

Figure 4. BCL9L Depletion Results in Intratumor Heterogeneity in Xenografts

(A) Experimental procedure for xenograft experiments. Stable shBCL9L HCT-116 cells were treated as in Figure 2H. Cells (2×10^6) were subcutaneously injected in each mouse flank.

(B) Engraftment efficiency of cells treated as described in (A) 60 days following injection.

(C) Growth curves of shCtrl and shBCL9L xenografts with and without reversine pre-treatment (mean ± SEM).

(D) Genome-wide multi-region SNP DNA copy-number analysis of six xenografts (three shCtrl and three shBCL9L). Red, gain; blue, loss.

number of resistant colonies in HCT-116 *TP53*-null and CL-40 cells, a CRC cell line harboring the most frequent *TP53* mutation in CRC (R248Q, Figure 3E). These data support the hypothesis that BCL9L depletion results in an additive survival effect in *TP53*-mutant cells and suggest that loss of *BCL9L* contributes to aneuploidy tolerance in both *TP53*-competent and mutant CRC.

Effect of *BCL9L* Loss on Xenograft Models of Tumorigenesis

To determine the role of *BCL9L* as an aneuploidy suppressor in vivo, we injected BCL9L-depleted or control cells into immunocompromised mice following the protocol shown in Figure 4A. We observed that reversine pre-treatment of shBCL9L cells dramatically improved the engraftment efficiency and growth rate when compared with the rest of the experimental situations (Figures 4B and 4C). Although untreated shBCL9L cells did not engraft better, they displayed a modest growth advantage when compared with untreated shCtrl cells, although these differences were not statistically significant (Figure 4C).

We hypothesized that the increased karyotypic diversity in *BCL9L*-deficient cells pre-treated with reversine (Figures 2J and 3C) might lead to clonal selection of advantageous karyotypes, promoting intratumor heterogeneity of whole chromosome aneuploidies in the mouse xenografts. SNP profiling of the xenografts (Figure 4D) detected ubiquitous alterations on chromosomes 8, 10, 16, and 17 that are known for parental HCT-116 cells, together with intratumor heterogeneity for whole chromosome 12 in one region of two BCL9L-depleted xenografts and whole chromosome 7 gain in one region of one BCL9L-depleted xenografts. Notably, whole chromosome gains were observed in shBCL9L cells both with and without reversine, substantiating the role of *BCL9L* loss in the tolerance and propagation of endogenous segregation errors. Control cells did not show any heterogeneous whole chromosome alterations. Gain of the long arm of chromosome 21 was seen in one region of one shCtrl xenograft. These data support the ability of BCL9L depletion to foster intratumor heterogeneity and the propagation of subclones with whole chromosome aneuploidies distinct from other subclones within the same tumor.

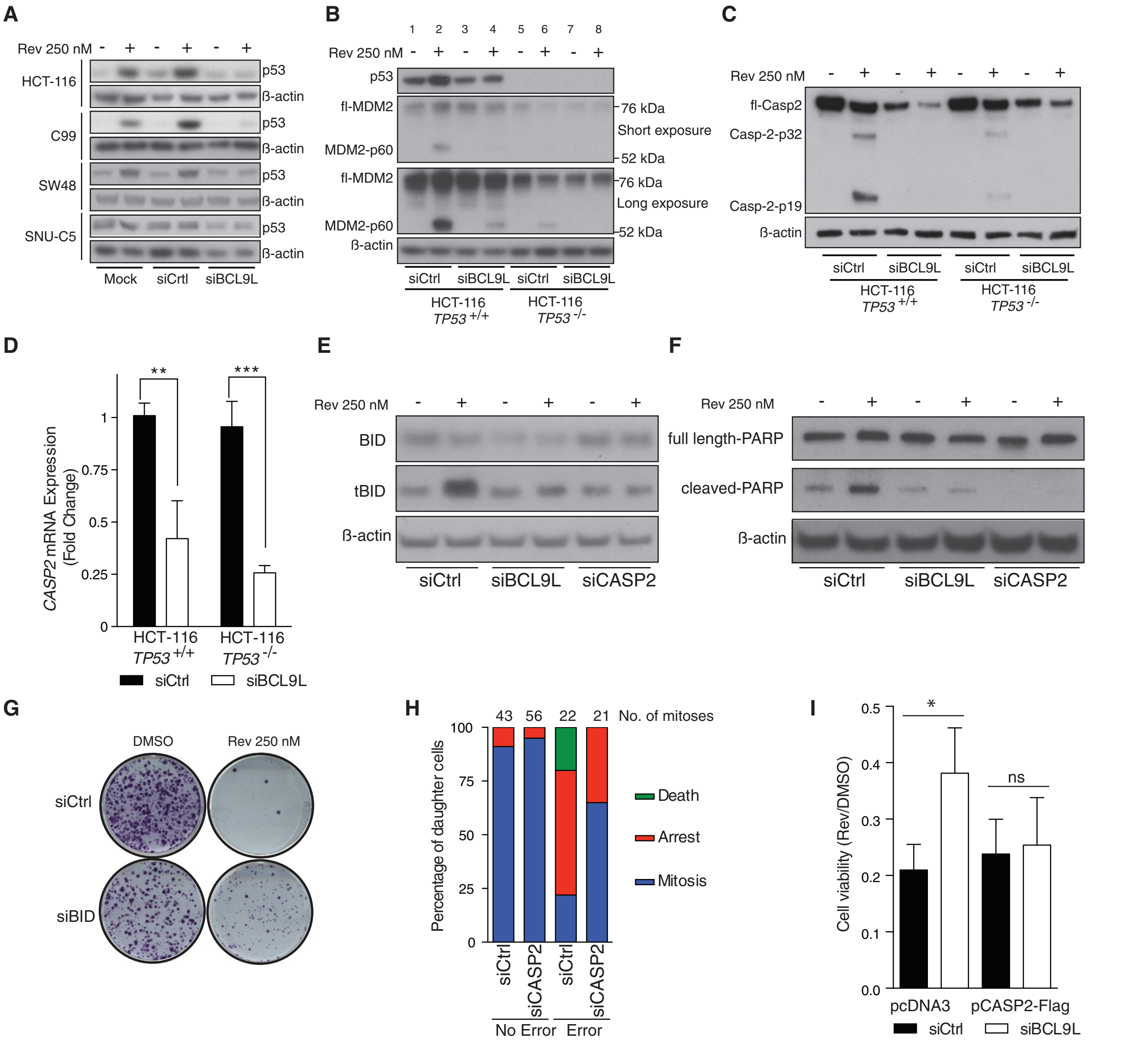

Figure 5. Mechanisms of *BCL9L*-Mediated Aneuploidy Tolerance

(A) Effect of BCL9L depletion on p53 protein levels in four near-diploid CRC cell lines following reversine treatment for 72 hr.

(B) MDM2 protein expression and cleavage in reversine-treated (72 hr) *TP53*-WT and *TP53*-null HCT-116 cells following BCL9L depletion (two exposures shown).

(C) Effect of BCL9L depletion on caspase-2 protein expression and cleavage in isogenic HCT-116 cells (72 hr).

(D) qPCR analysis of caspase-2 mRNA in BCL9L-depleted *TP53*-WT and *TP53*-null HCT-116 cells (n = 3; p value calculated by unpaired Student's t test).

(E) BID protein expression and cleavage (tBID) in reversine-treated *TP53*-null HCT-116 cells following BCL9L or caspase-2 depletion (72 hr).

(F) Effect of BCL9L and caspase-2 depletion on PARP cleavage in *TP53*-null HCT-116 cells (72 hr).

(G) Effect of *BID* silencing on colony-forming efficiency in reversine-treated *TP53*-null HCT-116 cells.

(H) Live-cell imaging analysis of daughter cell fate after normal mitosis and mitosis with endogenous segregation errors in caspase-2-depleted HCT-116 cells.

(I) Viability of cells at 72 hr after co-transfecting pcDNA3-caspase-2-FLAG or empty control together with the indicated siRNA in the presence and absence of 250 nM reversine (n = 3; p value calculated by paired Student's t test).

Error bars denote SD. ns, not significant. *p < 0.05, **p < 0.01, ***p < 0.001. See also Figures S6 and S7.

Mechanism of *BCL9L*-Mediated Aneuploidy Surveillance

p53 stabilization mediates apoptosis and cell cycle arrest upon genotoxic stress. Western blot analysis showed that *BCL9L* silencing strongly inhibited p53 accumulation following reversine treatment in *TP53*-WT HCT-116, SW48, and C99 cells, an effect reproduced with three siRNA duplexes (Figures 5A and S6A). We did not detect p53 accumulation in *TP53*-mutant SNU-C5 cells upon reversine treatment (Figure 5A). *BCL9L* silencing in HCT-116 did not affect *TP53* mRNA levels (Figure S6B). However, *BCL9L* silencing in HCT-116 inhibited the induction of the p53 transcriptional targets

CDKN1A (p21) and *BBC3* (Puma) after reversine treatment (Figure S6C).

We then examined MDM2 expression due to its important role in regulating p53 stability (Karni-Schmidt et al., 2016). Following reversine treatment, we observed an intense band around 60 kDa similar to the MDM2-p60 N-terminal cleavage product previously described (Oliver et al., 2011) (Figure 5B) that was not detected by C-terminal MDM2 antibodies (Figure S6D). MDM2-p60 accumulated mainly in the nucleus where it co-localized with p53 (Figure S6E). Importantly, MDM2 cleavage was still detectable in *TP53*-null cells following reversine exposure, and was impaired following *BCL9L* silencing and reversine treatment in both *TP53*-WT (Figure 5B, compare lanes 2 and 4) and *TP53*-null cells (Figure 5B, compare lanes 6 and 8).

Active caspase-2 cleaves MDM2, generating the MDM2-p60 fragment as part of a p53 regulatory cascade. MDM2-p60 conserves the p53 binding domain but is devoid of the RING domain. p60-p53 heterodimers cannot be targeted for degradation, which ultimately enhances p53 accumulation (Oliver et al., 2011; Terry et al., 2015). We observed that reversine treatment induced cleavage of caspase-2 (determined by cleavage of caspase-2 into p32 and p19 moieties) in both HCT-116 *TP53*-WT and to a lesser extent in *TP53*-null cells (Figure 5C). We also observed reduced levels of caspase-2 protein and mRNA in BCL9L-depleted HCT-116 *TP53*-WT and null cells (Figures 5C, 5D, and S5D), which contributed to lower levels of active caspase-2 upon reversine treatment. A reduction in caspase-2 protein following *BCL9L* silencing was also confirmed in other cell lines (Figure S6F) and with different siRNA sequences targeting *BCL9L* (Figure S6G). qPCR analysis revealed that reversine treatment did not increase the expression of *PIDD* mRNA, a gene involved in p53-dependent caspase-2 activation (Figure S6H). Cell synchronization and transient reversine exposure revealed that p53 stabilization, MDM2 cleavage, and caspase-2 activation are detectable after one division in the presence of reversine (doubling time for HCT-116 = 20 hr), confirming that one cell division is sufficient to trigger these three events (Figure S6I). Similar to *BCL9L* silencing, caspase-2 depletion by RNAi attenuated both p53 accumulation and MDM2 cleavage upon reversine treatment (Figures S7A and B).

These results suggest that reversine induces proteolytic activation of caspase-2 partially independent of p53, and depletion of BCL9L reduces caspase-2 expression that ultimately prevents cleavage of MDM2 and stabilization of p53 following reversine exposure.

Given the higher number of karyotypic alterations in CRC with co-occurrence of *BCL9L* and *TP53* alterations (Figure 1E) and our results showing a *BCL9L* survival effect in *TP53*-WT and null backgrounds (Figures 3D, 3E, S4C, and S4D), we investigated a potential p53-independent role for BCL9L in aneuploidy tolerance. Since caspase-2 cleavage was detectable, but at reduced levels, in *TP53*-null cells (Figure 5C), we assessed the role of other caspase-2 substrates, such as BID, in mediating aneuploidy tolerance (Guo et al., 2002). Although *BCL9L* silencing in *TP53*-null cells resulted in lower levels of basal *BID* mRNA (Figure S7C), only a moderate reduction in BID protein steady-state levels was observed (Figures 5E and S7D). In *TP53*-null cells, reversine treatment induced formation of a 15 kDa band consistent with tBID (Figures 5E and S7D), derived through caspase-mediated cleavage of BID. Silencing of either *BCL9L* or caspase-2 attenuated this cleavage (Figures 5E and S7D). tBID relocalizes to the outer mitochondrial membrane where it activates the mitochondrial apoptotic pathway (Korsmeyer et al., 2000). Consistent with a p53-independent pro-apoptotic role for BCL9L, BCL9L and caspase-2 depletion prevented poly(ADP-ribose)polymerase (PARP) cleavage in reversine-treated *TP53*-null HCT-116 cells (Figure 5F). Colony-forming assays confirmed that BID depletion by siRNA had a similar effect to *BCL9L* silencing in reversine-treated HCT-116 *TP53*-null cells (Figure 5G). We did not find significant changes in the expression of other caspases and mitochondrial apoptotic regulators (Figure S7E).

Consistent with the results shown above, caspase-2 depletion increased resistance to reversine treatment in BrdU incorporation assays (Figure S7F), and also increased tolerance to endogenous segregation errors in HCT-116 cells (Figure 5H). Finally, co-transfection of siRNA targeting BCL9L and a caspase-2 expression plasmid (Figure S7G) reverted the tolerance of reversine treatment mediated by BCL9L depletion (Figure 5I). These observations support a mechanism of aneuploidy tolerance whereby caspase-2 suppression in BCL9L-depleted cells enhances the survival of cancer cells after endogenous or drug-mediated segregation errors in both *TP53*-WT and *TP53*-null backgrounds.

Next, we explored the hypothesis that *BCL9L* loss drives aneuploidy tolerance through repression of Wnt signaling. BCL9/BCL9L and their binding partners β-catenin and Pygo function as transcriptional co-activators that facilitate the activity of the TCF/LEF family of transcription factors (de la Roche et al., 2008). We confirmed that *BCL9L* silencing inhibited TCF4 transcriptional activity in reporter assays and expression of Wnt signaling targets (Figures S8A and S8B). Examination of the ENCODE database (Rosenbloom et al., 2012) revealed a potential TCF4-binding site near the transcription start site of *CASP2* that we were able to confirm by TCF4 chromatin immunoprecipitation in HCT-116 cells (Figure 6A). Treatment of HCT-116 with PNU74654, a drug that inhibits Wnt signaling by impairing β-catenin binding to TCF4, triggered a statistically significant downregulation of the Wnt targets *AXIN2* and *MYC* along with reduction of caspase-2 mRNA and protein (Figures 6B and 6C). In addition, treatment of HCT-116 cells with PNU74654 induced reversine tolerance relative to HCT-116 cells treated with reversine alone (Figures 6D and 6E).

In summary, we propose a model in which partial loss of *BCL9L* results in lower caspase-2 mRNA and protein levels in both *TP53*-WT and mutant cells, likely mediated through inhibition of TCF4 transcriptional activity at the *CASP2* promoter. After chromosome segregation errors, fully functional BCL9L permits transcription and activation of caspase-2, resulting in p53 stabilization via MDM2 cleavage in *TP53*-WT cells and BID cleavage in *TP53*-mutant cells, ultimately inducing arrest and apoptosis (Figure 6F). In cancer cells, *BCL9L* dysfunction results in lower levels of caspase-2, and when chromosome missegregation occurs this deficiency results in suboptimal activation of caspase-2, leading to impaired p53 stabilization, tBID formation, and attenuated cell death.

DISCUSSION

Aneuploidy has prognostic relevance in multiple cancer types (Carter et al., 2006; Danielsen et al., 2016; Kronenwett et al.,

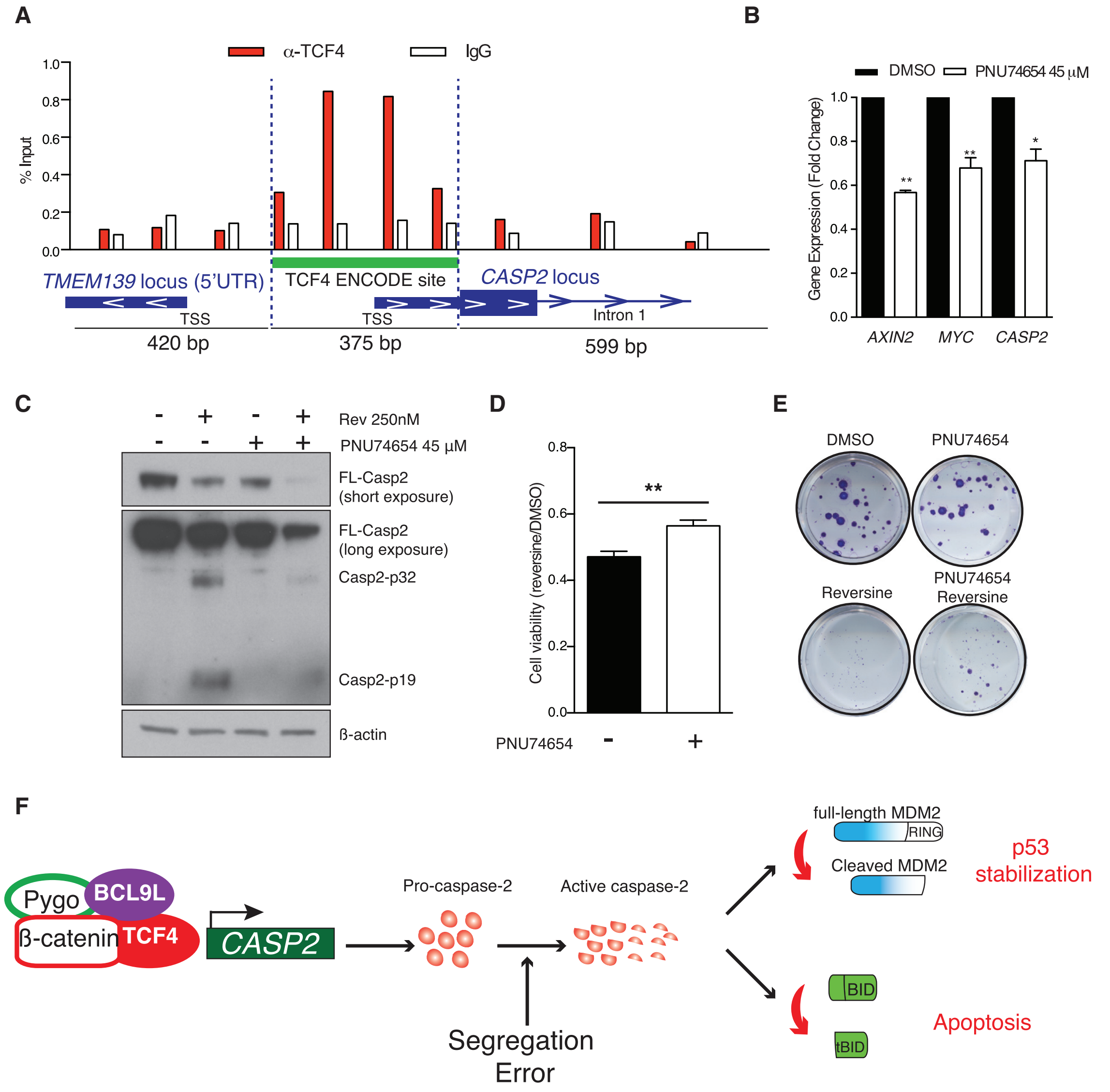

Figure 6. Mechanism of *BCL9L* in the Regulation of Caspase-2 Expression

(A) Chromatin immunoprecipitation of TCF4 and qPCR analysis of the immunoprecipitated DNA. Primers were designed across the caspase-2 promoter region that contains a TCF4 binding site annotated in ENCODE (bars are located approximately in the center of the resulting amplicon). TSS, transcription start site; IgG, immunoglobulin G.

(B) mRNA expression analysis of caspase-2 and the Wnt targets *AXIN2* and *MYC* following treatment with 45 μM PNU74654 (n = 3; p values calculated by unpaired Student's t test).

(C) Effect of PNU74654 on caspase-2 protein with and without reversine treatment.

(D) Effect of PNU74654 on cell viability following reversine treatment in HCT-116 cells (mean ± SD, n = 3).

(E) Colony-forming efficiency of HCT-116 cells treated with reversine in the presence or absence of 45 μM PNU74654.

(F) Proposed mechanism of *BCL9L* in aneuploidy surveillance.

Error bars denote SD. *p < 0.05, **p < 0.01. See also Figure S8.

2004; Mouradov et al., 2013; Yoo et al., 2010) and is associated with cancer multidrug resistance (Kuznetsova et al., 2015; Lee et al., 2011; Swanton et al., 2006). These high-risk features of CIN suggest that targeting aneuploid cancer cell populations may have therapeutic potential, emphasizing the importance of understanding the cellular processes that initiate and promote tolerance of aneuploidy.

Tumors harbor a wide spectrum of structural and numerical chromosomal alterations (Andor et al., 2015) ranging from diploid or near-diploid tumors to highly aneuploid samples with more complex karyotypes. Notwithstanding that p53 is closely associated with CIN and aneuploidy in CRC, little is known about somatic events that might cooperate with p53 dysfunction in generating or sustaining the accumulation of chromosomal alterations. Our data provide support for *BCL9L* as an aneuploidy tumor-suppressor gene in CRC, the loss of which sustains aneuploidy tolerance, both independently of and in cooperation with p53, through repression of caspase-2. These results are

supported by studies in caspase-2 knockout mouse models in which transformed cells develop aneuploidy and become more aggressive (Dorstyn et al., 2012; Puccini et al., 2013).

The mechanisms leading to p53 accumulation in response to chromosomal missegregation events are unclear. DNA damage (Janssen et al., 2011), histone phosphorylation (Hinchcliffe et al., 2016) and reactive oxygen species (Li et al., 2010) have all been proposed as mechanisms of p53 accumulation in CIN cells. Our data reveal that caspase-2 depletion induces tolerance of endogenous chromosome segregation errors and prevents p53 accumulation in response to artificial induction of chromosome segregation errors using an Mps1 inhibitor, reversine, supporting a central role for caspase-2 as an enzyme regulating p53, underpinned by seminal work from other groups (Dorstyn et al., 2012; Oliver et al., 2011; Terry et al., 2015). We found that loss of *BCL9L* prevents cleavage of BID through caspase-2 in *TP53*-null cells and thereby inhibits apoptosis. This p53-independent role for caspase-2 in the suppression of aneuploidy might operate as a fail-safe mechanism to limit CIN in *TP53*-mutant tumors, thereby compromising outgrowth of heterogeneous tumor cells and impairing subsequent tumor adaptation. Conceivably, in *TP53*-WT cells parallel mechanisms of aneuploidy surveillance independent of p53 might reinforce the removal of aneuploid cells in instances where chromosome missegregation events may remain undetected by p53.

Our results support the possibility that caspase-2 can be activated upstream of p53 after chromosome missegregation. This emphasizes the need to elucidate the mechanisms of caspase-2 activation, such as its dependence on phosphorylation (Andersen et al., 2009) or proteotoxic stress, frequently observed in aneuploid cells (Upton et al., 2008). This process might constitute a mechanism of genome instability sensing that can operate independently of p53.

Although evidence supports roles for both BCL9L and BCL9 in the regulation of gene expression that are independent of β-catenin (Cantu et al., 2014), our data suggest that caspase-2 is a target of the β-catenin/TCF4 transcriptional complex. However, it is unclear whether BCL9L regulates a specific subset of genes distinct from its homolog BCL9. Our data support the "just-right" model (Albuquerque et al., 2002) for the modulation of Wnt signaling in tumors to render TCF transcriptional activation sufficient for cancer cell viability, while minimizing transcriptional activation of genes associated with cell death. More specifically, partial inhibition of TCF4 transcriptional activity in tumors with excessive Wnt pathway activation through *BCL9L* dysfunction might reduce caspase-2 expression to levels compatible with cell viability, enhancing tolerance of segregation errors and intratumor heterogeneity.

Consistent with these observations, and notwithstanding the limitations of the xenograft evolutionary experiments due to animal welfare considerations and the resulting short time course of such studies, the xenograft data support the ability of *BCL9L* silencing to propagate intratumor heterogeneity manifested as whole chromosome aneuploidies that are spatially distinct within individual tumors.

Based on the genomic analysis presented here, the characterization of *BCL9L* as an aneuploidy suppressor conforms to a haploinsufficiency model based on results from our analyses of CRC datasets and our functional work. This model has been frequently described for other tumor suppressors such as *PTEN*, *BRCA1*, and *RAD17* (Berger et al., 2011). Such observations may begin to explain why the identification of aneuploidy suppressors has proved evasive, and suggest the need for deeper analysis of genomic datasets focusing on haploinsufficiency as a possible mechanism of tolerance to large-scale karyotypic alterations.

In summary, these data support a role for *BCL9L* as an aneuploidy tolerance gene, conforming to criteria for a significantly mutated driver gene and tumor suppressor in CRC (Giannakis et al., 2016; Tamborero et al., 2013; Vogelstein et al., 2013). Understanding aneuploidy tolerance mechanisms more widely, and the BCL9L/caspase-2/BID axis specifically, may unravel potential vulnerabilities in aneuploid cancers, which could be exploited to limit intercellular heterogeneity, a substrate for selection and tumor evolution.

EXPERIMENTAL PROCEDURES

Patient Samples

Tissue collection was approved by an ethics committee (NRS Committee South Central-Oxford B, REC reference 05/Q1605/66), and all individuals included in this study had provided written informed consent for the analysis presented.

Reversine Survival Screen

Five thousand HCT-116 cells per well were seeded on 96-well plates with siRNA transfection medium and DMSO or 250 nM reversine. Cells were grown for 3 days and cell viability was measured by Cell Titer Blue (Promega). The surviving fraction for each siRNA pool was calculated as the ratio of the fluorescent Cell Titer Blue signal of treated wells between untreated wells (four replicates). Data shown in Figure 1A were normalized to siCtrl2 (siGENOME).

Reversine Survival Assays

For siRNA short-term survival, cells were plated in transfection medium and 250 nM reversine for 3 days. Cell number was measured by DAPI staining and automated imaging (Acumen), cell viability was measured with Cell Titer Blue (Promega) or alternatively, cells were harvested and analyzed after 1 hr of BrdU incorporation. For colony-forming assays with siRNA, cells were transfected for 3 days and replated in serial dilutions in the presence or absence of reversine. After 5 days of treatment, reversine-containing medium was replaced by drug-free medium and cell colonies grown until the appropriate size was reached.

For long-term colony-forming assays (shRNA), cells were treated for 15 days with 125 nM reversine.

FISH Analysis

For development of aneuploidy, stable BCL9L knockdown and control cells were treated for 15 days with 125 nM reversine. Reversine-containing medium was replaced by drug-free medium and cells were allowed to recover for 2 weeks. Next, cells were grown on glass slides and centromeric FISH was performed (CEP 1, 2, 8, and 15; Abbot Molecular). Centromeric signals were counted and modal centromeric variation was calculated as the fraction of cells with centromeric signals different from the modal number within the population.

Animal Procedures

All animal regulated procedures were approved by The Francis Crick Institute BRF Strategic Oversight Committee that incorporates the Animal Welfare and Ethical Review Body and conformed with UK Home Office guidelines and regulations under the Animals (Scientific Procedures) Act 1986 including Amendment Regulations 2012.

Statistical Methods

For a complete description see Supplemental Experimental Procedures.

ACCESSION NUMBERS

The accession number for whole-exome sequencing and SNP array data reported in this paper is EGA: EGASS00001001907.

SUPPLEMENTAL INFORMATION

Supplemental Information includes Supplemental Experimental Procedures, eight figures, four tables, and four movies and can be found with this article online at http://dx.doi.org/10.1016/j.ccell.2016.11.001.

AUTHOR CONTRIBUTIONS

Conceptualization, C.L.-G., L.S., I.T., and C.S.; Methodology, C.L.-G., L.S., N.McG., E.G., A.J.R., R.B., H.D., and C.S.; Software, Formal Analysis, and Data Curation, C.L.-G., N.McG., N.J.B., S.H., F.F., A.S., M.K., and C.S.; Investigation and Validation, C.L.-G., L.S., S.H., E.G., A.J.R., N.M., S.B., B.P., D.O., M.N., and R.B.; Resources, A.J.R., E.D., H.D., G.S., B.S.-D., and I.T.; Writing – Original Draft, C.L.-G. and C.S.; Writing – Review and Editing and Visualization, C.L.-G., L.S., N.McG., S.H., N.J.B., E.G., I.T., and C.S.; Supervision, C.L.-G., I.T. M.N., H.D., and C.S.; Funding Acquisition, C.S.

ACKNOWLEDGMENTS

We thank FACS, Cell Services, Experimental Histopathology, High-Throughput Screening, Equipment Park, and Biological Research core facilities at The Francis Crick Institute for providing reagents, processing and analysis of samples, and insightful discussions. We specially thank Levi Garraway and Jasmine Mu (Dana-Farber/Harvard Cancer Center) for sharing sequencing data. The results published here are partly based upon data generated the TCGA research network (http://cancergenome.nih.gov/). The data were retrieved through dbGaP authorization (accession no. phs000178.v9.p8). C.S. is Royal Society Napier Research Professor. This work was supported by the Francis Crick Institute which receives its core funding from Cancer Research UK (FC001169), the UK Medical Research Council (FC001169), and the Wellcome Trust (FC001169); and by the UK Medical Research Council (grant reference MR/FC001169/1). C.S. is funded by Cancer Research UK (TRACERx), the CRUK Lung Cancer Center of Excellence, Stand Up 2 Cancer (SU2C), the Rosetrees Trust, NovoNordisk Foundation (ID 16584 to L.S.), the Prostate Cancer Foundation, the Breast Cancer Research Foundation (BCRF), the European Research Council (THESEUS) (617844 to E.G.), and Marie Curie Network PloidyNet. Support was provided to C.S. by the National Institute for Health Research, the University College London Hospitals Biomedical Research Center, and the Cancer Research UK University College London Experimental Cancer Medicine Center. This project was also funded by The Wellcome Trust Center for Human Genetics (090532/Z/09/Z to I.T. E.D. and M.K.).

Received: February 9, 2016
Revised: August 5, 2016
Accepted: October 28, 2016
Published: January 9, 2017

REFERENCES

Albuquerque, C., Breukel, C., van del Luijt, R., Fidalgo, P., Lage, P., Slors, F.J.M., Nobre-Leitao, C., Fodde, R., and Smits, R. (2002). The ‘‘just-right’’ signaling model: APC somatic mutations are selected based on a specific level of activation of the β-catenin signaling cascade. Hum. Mol. Genet. *11*, 1549–1560.

Andersen, J.L., Johnson, C.E., Freel, C.D., Parrish, A.B., Day, J.L., Buchakjian, M.R., Nutt, L.K., Thompson, J.W., Moseley, M.A., and Kornbluth, S. (2009). Restraint of apoptosis during mitosis through interdomain phosphorylation of caspase-2. EMBO J. *28*, 3216–3227.

Andor, N., Graham, T.A., Jansen, M., Xia, L.C., Aktipis, C.A., Petritsch, C., Ji, H.P., and Maley, C.C. (2015). Pan-cancer analysis of the extent and consequences of intratumor heterogeneity. Nat. Med. *22*, 105–113.

Bakhoum, S.F., Genovese, G., and Compton, D.A. (2009a). Deviant kinetochore microtubule dynamics underlie chromosomal instability. Curr. Biol. *19*, 1937–1942.

Bakhoum, S.F., Thompson, S.L., Manning, A.L., and Compton, D.A. (2009b). Genome stability is ensured by temporal control of kinetochore-microtubule dynamics. Nat. Cell Biol. *11*, 27–35.

Berger, A.H., Knudson, A.G., and Pandolfi, P.P. (2011). A continuum model for tumor suppression. Nature *476*, 163–169.

Bogaert, J., and Prenen, H. (2014). Molecular genetics of colorectal cancer. Ann. Gastroenterol. *27*, 9–14.

Boutros, P.C., Fraser, M., Harding, N.J., de Borja, R., Trudel, D., Lalonde, E., Meng, A., Hennings-Yeomans, P.H., McPherson, A., Sabelnykova, V.Y., et al. (2015). Spatial genomic heterogeneity within localized, multifocal prostate cancer. Nat. Gen. *47*, 736–745.

Burrell, R.A., McClelland, S.E., Endesfelder, D., Groth, P., Weller, M.C., Shaikh, N., Domingo, E., Kanu, N., Dewhurst, S.M., Gronroos, E., et al. (2013). Replication stress links structural and numerical cancer chromosomal instability. Nature *494*, 492–496.

Cahill, D.P., Lengauer, C., Yu, J., Riggins, G.J., Willson, J.K., Markowitz, S.D., Kinzler, K.W., and Vogelstein, B. (1998). Mutations of mitotic checkpoint genes in human cancers. Nature *392*, 300–303.

Cancer Genome Atlas Network. (2012). Comprehensive molecular characterization of human colon and rectal cancer. Nature *487*, 330–337.

Cantu, C., Zimmerli, D., Hausmann, G., Valenta, T., Moor, A., Aguet, M., and Basler, K. (2014). Pax6-dependent, but beta-catenin-independent, function of Bcl9 proteins in mouse lens development. Genes Dev. *28*, 1879–1884.

Carter, S.L., Eklund, A.C., Kohane, I.S., Harris, L.N., and Szallasi, Z. (2006). A signature of chromosomal instability inferred from gene expression profiles predicts clinical outcome in multiple human cancers. Nat. Gen. *38*, 1043–1048.

Chan, K.L., North, P.S., and Hickson, I.D. (2007). BLM is required for faithful chromosome segregation and its localization defines a class of ultrafine anaphase bridges. EMBO J. *26*, 3397–3409.

Chin, S.F., Teschendorff, A.E., Marioni, J.C., Wang, Y., Barbosa-Morais, N.L., Thorne, N.P., Costa, J.L., Pinder, S.E., van de Wiel, M.A., Green, A.R., et al. (2007). High-resolution aCGH and expression profiling identifies a novel genomic subtype of ER negative breast cancer. Genome Biol. *8*, R215.

Cianchi, F., Balzi, M., Becciolini, A., Giache, V., Messerini, L., Palomba, A., Tisti, E., Faraoni, P., Chellini, F., Pucciani, F., et al. (1999). Correlation between DNA content and p53 deletion in colorectal cancer. Eur. J. Sur. *165*, 363–368.

Crasta, K., Ganem, N.J., Dagher, R., Lantermann, A.B., Ivanova, E.V., Pan, Y., Nezi, L., Protopopov, A., Chowdhury, D., and Pellman, D. (2012). DNA breaks and chromosome pulverization from errors in mitosis. Nature *482*, 53–58.

Danielsen, H.E., Pradhan, M., and Novelli, M. (2016). Revisiting Tumor aneuploidy - the place of ploidy assessment in the molecular era. Nat. Rev. Clin. Oncol. *13*, 291–304.

de la Roche, M., Worm, J., and Bienz, M. (2008). The function of BCL9 in Wnt/beta-catenin signaling and colorectal cancer cells. BMC Cancer *8*, 199.

Desmedt, C., Fumagalli, D., Pietri, E., Zoppoli, G., Brown, D., Nik-Zainal, S., Gundem, G., Rothe, F., Majjaj, S., Garuti, A., et al. (2015). Uncovering the genomic heterogeneity of multifocal breast cancer. J. Pathol. *236*, 457–466.

Dewhurst, S.M., McGranahan, N., Burrell, R.A., Rowan, A.J., Gronroos, E., Endesfelder, D., Joshi, T., Mouradov, D., Gibbs, P., Ward, R.L., et al. (2014). Tolerance of whole-genome doubling propagates chromosomal instability and accelerates cancer genome evolution. Cancer Discov. *4*, 175–185.

Ding, L., Ley, T.J., Larson, D.E., Miller, C.A., Koboldt, D.C., Welch, J.S., Ritchey, J.K., Young, M.A., Lamprecht, T., McLellan, M.D., et al. (2012). Clonal evolution in relapsed acute myeloid leukaemia revealed by whole-genome sequencing. Nature *481*, 506–510.

Dorstyn, L., Puccini, J., Wilson, C.H., Shalini, S., Nicola, M., Moore, S., and Kumar, S. (2012). Caspase-2 deficiency promotes aberrant DNA-damage response and genetic instability. Cell Death Differ. *19*, 1288–1298.

Gaasenbeek, M., Howarth, K., Rowan, A.J., Gorman, P.A., Jones, A., Chaplin, T., Liu, Y., Bicknell, D., Davison, E.J., Fiegler, H., et al. (2006). Combined array-comparative genomic hybridization and single-nucleotide polymorphism-loss

of heterozygosity analysis reveals complex changes and multiple forms of chromosomal instability in colorectal cancers. Cancer Res. *66*, 3471–3479.

Gascoigne, K.E., and Taylor, S.S. (2008). Cancer cells display profound intra- and interline variation following exposure to antimitotic drugs. Cancer Cell *12*, 111–122.

Giannakis, M., Mu, X.J., Shukla, S.A., Qian, Z.R., Cohen, O., Nishihara, R., Bahl, S., Cao, Y., Amin-Mansour, A., Yamauchi, M., et al. (2016). Genomic correlates of immune-cell infiltrates in colorectal carcinoma. Cell Rep. *15*, 857–865.

Grim, J.E., Knoblaugh, S.E., Guthrie, K.A., Hagar, A., Swanger, J., Hespelt, J., Delrow, J.J., Small, T., Grady, W.M., Nakayama, K.I., and Clurman, B.E. (2012). Fbw7 and p53 cooperatively suppress advanced and chromosomally unstable intestinal cancer. Mol. Cell. Biol. *32*, 2160–2167.

Guo, Y., Srinivasula, S.M., Druilhe, A., Fernandes-Alnemri, T., and Alnemri, E.S. (2002). Caspase-2 induces apoptosis by releasing proapoptotic proteins from mitochondria. J. Biol. Chem. *277*, 13430–13437.

Hinchcliffe, E.H., Day, C.A., Karanjeet, K.B., Fadness, S., Langfald, A., Vaughan, K.T., and Dong, Z. (2016). Chromosome missegregation during anaphase triggers p53 cell cycle arrest through histone H3.3 Ser31 phosphorylation. Nat. Cell Biol. *18*, 668–675.

Iwanaga, Y., Chi, Y.H., Miyazato, A., Sheleg, S., Haller, K., Peloponese, J.M., Jr., Li, Y., Ward, J.M., Benezra, R., and Jeang, K.T. (2007). Heterozygous deletion of mitotic arrest-deficient protein 1 (MAD1) increases the incidence of tumors in mice. Cancer Res. *67*, 160–166.

Janssen, A., van der Burg, M., Szuhai, K., Kops, G.J., and Medema, R.H. (2011). Chromosome segregation errors as a cause of DNA damage and structural chromosome aberrations. Science *333*, 1895–1898.

Jemaa, M., Galluzzi, L., Keep, O., Boileve, A., Lissa, D., Senovilla, L., Harper, F., Pierron, G., Berardinelli, F., Antoccia, A., et al. (2012). Preferential killing of p53-deficient cancer cells by reversine. Cell Cycle *11*, 2149–2158.

Karni-Schmidt, O., Lokshin, M., and Prives, C. (2016). The roles for MDM2 and MDMX in cancer. Annu. Rev. Pathol. *11*, 617–644.

Kawabata, T., Luebben, S.W., Yamaguchi, S., Ilves, I., Matise, I., Buske, T., Botchan, M.R., and Shima, N. (2011). Stalled fork rescue via dormant replication origins in unchallenged S phase promotes proper chromosome segregation and tumor suppression. Mol. Cell *41*, 543–553.

Korsmeyer, S.J., Wei, M.C., Saito, M., Weiler, S., Oh, K.J., and Schlesinger, P.H. (2000). Pro-apoptotic cascade activates BID, which oligomerizes BAK or BAX into pores that result in the release of cytochrome c. Cell Death Differ. *7*, 1166–1173.

Kronenwett, U., Huwendiek, S., Ostring, C., Portwood, N., Roblick, U.J., Pawitan, Y., Alaiya, A., Sennerstam, R., Zetterberg, A., and Auer, G. (2004). Improved grading of breast adenocarcinomas based on genomic instability. Cancer Res. *64*, 904–909.

Kuznetsova, A.Y., Seget, K., Moeller, G.K., de Pagter, M.S., de Roos, J.A., Durrbaum, M., Kuffer, C., Muller, S., Zaman, G.J., Kloosterman, W.P., et al. (2015). Chromosomal instability, tolerance of mitotic errors and multidrug resistance are promoted by tetraploidization in human cells. Cell Cycle *14*, 2810–2820.

Landau, D.A., Carter, S.L., Stojanov, P., McKenna, A., Stevenson, K., Lawrence, M.S., Sougnez, C., Stewart, C., Sivachenko, A., Wang, L., et al. (2013). Evolution and impact of subclonal mutations in chronic lymphocytic leukemia. Cell *152*, 714–726.

Lee, A.J., Endesfelder, D., Rowan, A.J., Walther, A., Birkbak, N.J., Futreal, P.A., Downward, J., Szallasi, Z., Tomlinson, I.P., Howell, M., et al. (2011). Chromosomal instability confers intrinsic multidrug resistance. Cancer Res. *71*, 1858–1870.

Lengauer, C., Kinzler, K.W., and Vogelstein, B. (1997). Genetic instability in colorectal cancer. Nature *386*, 623–627.

Lengauer, C., Kinzler, K.W., and Vogelstein, B. (1998). Genetic instabilities in human cancers. Nature *396*, 643–649.

Li, M., Fang, X., Baker, D.J., Guo, L., Gao, X., Wei, Z., Han, S., van Deursen, J.M., and Zhang, P. (2010). The ATM-p53 pathway suppresses aneuploidy-induced tumorigenesis. Proc. Natl. Acad. Sci. USA *107*, 14188–14193.

Mouradov, D., Domingo, E., Gibbs, P., Jorissen, R.N., Li, S., Soo, P.Y., Lipton, L., Desai, J., Danielsen, H.E., Oukrif, D., et al. (2013). Survival in stage II/III colorectal cancer is independently predicted by chromosomal and microsatellite instability, but not by specific driver mutations. Am. J. Gastroenterol. *108*, 1785–1793.

Negrini, S., Gorgoulis, V.G., and Halazonetis, T.D. (2010). Genomic instability–an evolving hallmark of cancer. Nat. Rev. Mol. Cell Biol. *11*, 220–228.

Oliver, T.G., Meylan, E., Chang, G.P., Xue, W., Burke, J.R., Humpton, T.J., Hubbard, D., Bhutkar, A., and Jacks, T. (2011). Caspase-2-mediated cleavage of Mdm2 creates a p53-induced positive feedback loop. Mol. Cell *43*, 57–71.

Orsetti, B., Selves, J., Bascoul-Mollevi, C., Lasorsa, L., Gordien, K., Bibeau, F., Massemin, B., Paraf, F., Soubeyran, I., Hostein, I., et al. (2014). Impact of chromosomal instability on colorectal cancer progression and outcome. BMC Cancer *14*, 121.

Pampalona, J., Soler, D., Genesca, A., and Tusell, L. (2010). Whole chromosome loss is promoted by telomere dysfunction in primary cells. Genes Chromosomes Cancer *49*, 368–378.

Puccini, J., Shalini, S., Voss, A.K., Gatei, M., Wilson, C.H., Hiwase, D.K., Lavin, M.F., Dorstyn, L., and Kumar, S. (2013). Loss of caspase-2 augments lymphomagenesis and enhances genomic instability in Atm-deficient mice. Proc. Natl. Acad. Sci. USA *110*, 19920–19925.

Rosenbloom, K.R., Dreszer, T.R., Long, J.C., Malladi, V.S., Sloan, C.A., Raney, B.J., Cline, M.S., Karolchik, D., Barber, G.P., Clawson, H., et al. (2012). ENCODE whole-genome data in the UCSC Genome Browser: update 2012. Nucleic Acids Res. *40*, D912–D917.

Rowan, A., Halford, S., Gaasenbeek, M., Kemp, Z., Sieber, O., Volikos, E., Douglas, E., Fiegler, H., Carter, N., Talbot, I., et al. (2005). Refining molecular analysis in the pathways of colorectal carcinogenesis. Clin. Gastroenterol. Hepatol. *3*, 1115–1123.

Santaguida, S., Tighe, A., D'Alise, A.M., Taylor, S.S., and Musacchio, A. (2010). Dissecting the role of MPS1 in chromosome biorientation and the spindle checkpoint through the small molecule inhibitor reversine. J. Cell Biol. *190*, 73–87.

Sotillo, R., Hernando, E., Diaz-Rodriguez, E., Teruya-Feldstein, J., Cordon-Cardo, C., Lowe, S.W., and Benezra, R. (2007). Mad2 overexpression promotes aneuploidy and tumorigenesis in mice. Cancer Cell *11*, 9–23.

Stingele, S., Stoehr, G., and Storchova, Z. (2013). Activation of autophagy in cells with abnormal karyotype. Autophagy *9*, 246–248.

Swanton, C., Tomlinson, I., and Downward, J. (2006). Chromosomal instability, colorectal cancer and taxane resistance. Cell Cycle *5*, 818–823.

Szerlip, N.J., Pedraza, A., Chakravarty, D., Azim, M., McGuire, J., Fang, Y., Ozawa, T., Holland, E.C., Huse, J.T., Jhanwar, S., et al. (2012). Intratumoral heterogeneity of receptor tyrosine kinases EGFR and PDGFRA amplification in glioblastoma defines subpopulations with distinct growth factor response. Proc. Natl. Acad. Sci. USA *109*, 3041–3046.

Tamborero, D., Gonzalez-Perez, A., Perez-Llamas, C., Deu-Pons, J., Kandoth, C., Reimand, J., Lawrence, M.S., Getz, G., Bader, G.D., Ding, L., and Lopez-Bigas, N. (2013). Comprehensive identification of mutational cancer driver genes across 12 tumor types. Sci. Rep. *3*, 2650.

Tang, R., Changchien, C.R., Wu, M.C., Fan, C.W., Liu, K.W., Chen, J.S., Chien, H.T., and Hsieh, L.L. (2004). Colorectal cancer without high microsatellite instability and chromosomal instability–an alternative genetic pathway to human colorectal cancer. Carcinogenesis *25*, 841–846.

Terry, M.R., Arya, R., Mukhopadhyay, A., Berrett, K.C., Clair, P.M., Witt, B., Salama, M.E., Bhutkar, A., and Oliver, T.G. (2015). Caspase-2 impacts lung tumorigenesis and chemotherapy response in vivo. Cell Death Differ. *22*, 719–730.

Thompson, S.L., and Compton, D.A. (2008). Examining the link between chromosomal instability and aneuploidy in human cells. J. Cell Biol. *180*, 665–672.

Thompson, S.L., and Compton, D.A. (2010). Proliferation of aneuploid human cells is limited by a p53-dependent mechanism. J. Cell Biol. *188*, 369–381.

Torres, E.M., Dephoure, N., Panneerselvam, A., Tucker, C.M., Whittaker, C.A., Gygi, S.P., Dunham, M.J., and Amon, A. (2010). Identification of aneuploidy-tolerating mutations. Cell *143*, 71–83.

Upton, J.P., Austgen, K., Nishino, M., Coakley, K.M., Hagen, A., Han, D., Papa, F.R., and Oakes, S.A. (2008). Caspase-2 cleavage of BID is a critical apoptotic

signal downstream of endoplasmic reticulum stress. Mol. Cell. Biol. *28*, 3943–3951.

Vogelstein, B., Papadopoulos, N., Velculescu, V.E., Zhou, S., Diaz, L.A., Jr., and Kinzler, K.W. (2013). Cancer genome landscapes. Science *339*, 1546–1558.

Yates, L.R., Gerstung, M., Knappskog, S., Desmedt, C., Gundem, G., Van Loo, P., Aas, T., Alexandrov, L.B., Larsimont, D., Davies, H., et al. (2015). Subclonal diversification of primary breast cancer revealed by multiregion sequencing. Nat. Med. *21*, 751–759.

Yoo, J.W., Seo, K.W., Jang, S.J., Oh, Y.M., Shim, T.S., Kim, W.S., Lee, D.S., Lee, S.D., and Choi, C.M. (2010). The relationship between the presence of chromosomal instability and prognosis of squamous cell carcinoma of the lung: fluorescence in situ hybridization analysis of paraffin-embedded tissue from 47 Korean patients. J. Korean Med. Sci. *25*, 863–867.

Cancer Cell, Volume *31*

Supplemental Information

BCL9L Dysfunction Impairs Caspase-2 Expression Permitting Aneuploidy Tolerance in Colorectal Cancer

Carlos López-García, Laurent Sansregret, Enric Domingo, Nicholas McGranahan, Sebastijan Hobor, Nicolai Juul Birkbak, Stuart Horswell, Eva Grönroos, Francesco Favero, Andrew J. Rowan, Nicholas Matthews, Sharmin Begum, Benjamin Phillimore, Rebecca Burrell, Dahmane Oukrif, Bradley Spencer-Dene, Michal Kovac, Gordon Stamp, Aengus Stewart, Havard Danielsen, Marco Novelli, Ian Tomlinson, and Charles Swanton

SUPPLEMENTAL DATA

Table S1: Related to Table 1. Clinical features of the whole-exome sequenced cohort of 17 MSS colorectal adenocarcinomas.

Tumor	Sex	Age	Differentiation	Tumor Classification	Dukes stage	TNM	RLyV	Liver mets
**361**	M	66	Well/moderate	Adenocarcinoma	C1	pT3 N1 Mx	R0 Ly1 V1	Yes
**363**	M	52	Moderate	Adenocarcinoma	N.R.[(a)]	pT4 N0 Mx	R0 Ly0 V0	Yes
**365**	F	66	Moderate	Adenocarcinoma	A	pT2 N0 Mx	R0 Ly0 V0	Yes
**367**	M	76	Moderate	Adenocarcinoma	C1	pT3 N1 Mx	R0 Ly0 V0	Yes
**369**	M	48	Moderate	Adenocarcinoma	C1	pT3,N1,M1	R0 Ly0 V0	Yes
**371**	F	79	Moderate	Mucinous adenocarcinoma	B	ypT4 N0 Mx	R0 Ly0 V0	No
**373**	M	29	Moderate	Mucinous adenocarcinoma	C1	pT3 N2 M1	Ly1 V1	Yes
**375**	F	70	Well/moderate	Adenocarcinoma	B	pT3 N0 Mx	R0 Ly0 V0	No
**377**	M	61	Moderate	Adenocarcinoma	A	pT2 N0 Mx	R0 Ly0 V1	No
**379**	M	68	Moderate	Mucinous adenocarcinoma	A	pT2 N0 Mx	R0 Ly0 V0	No
**381**	F	64	Moderate	Adenocarcinoma	A	pT2 N0 Mx	R0 Ly0 V0	No
**383**	F	74	Moderate	Adenocarcinoma	A	pT2 N0 Mx	R0 Ly0 V0	No
**385**	M	70	Well/moderate	Adenocarcinoma	C1	T4 N2 M0	R0 Ly0 V0	No
**389**	M	58	Well/moderate	Adenocarcinoma	B	pT3 N0 Mx	R0Ly0V0	No
**391**	M	70	Well/moderate	Adenocarcinoma	A	pT2 N0 Mx	R0Ly0V0	No
**395**	M	59	Poor	Adenocarcinoma	B	pT3 N0 Mx	R0 Ly0 V1	No
**397**	M	74	Moderate	Adenocarcinoma	B	pT4 N0 Mx	R0 Ly0 V0	No

([a]) Not reported.

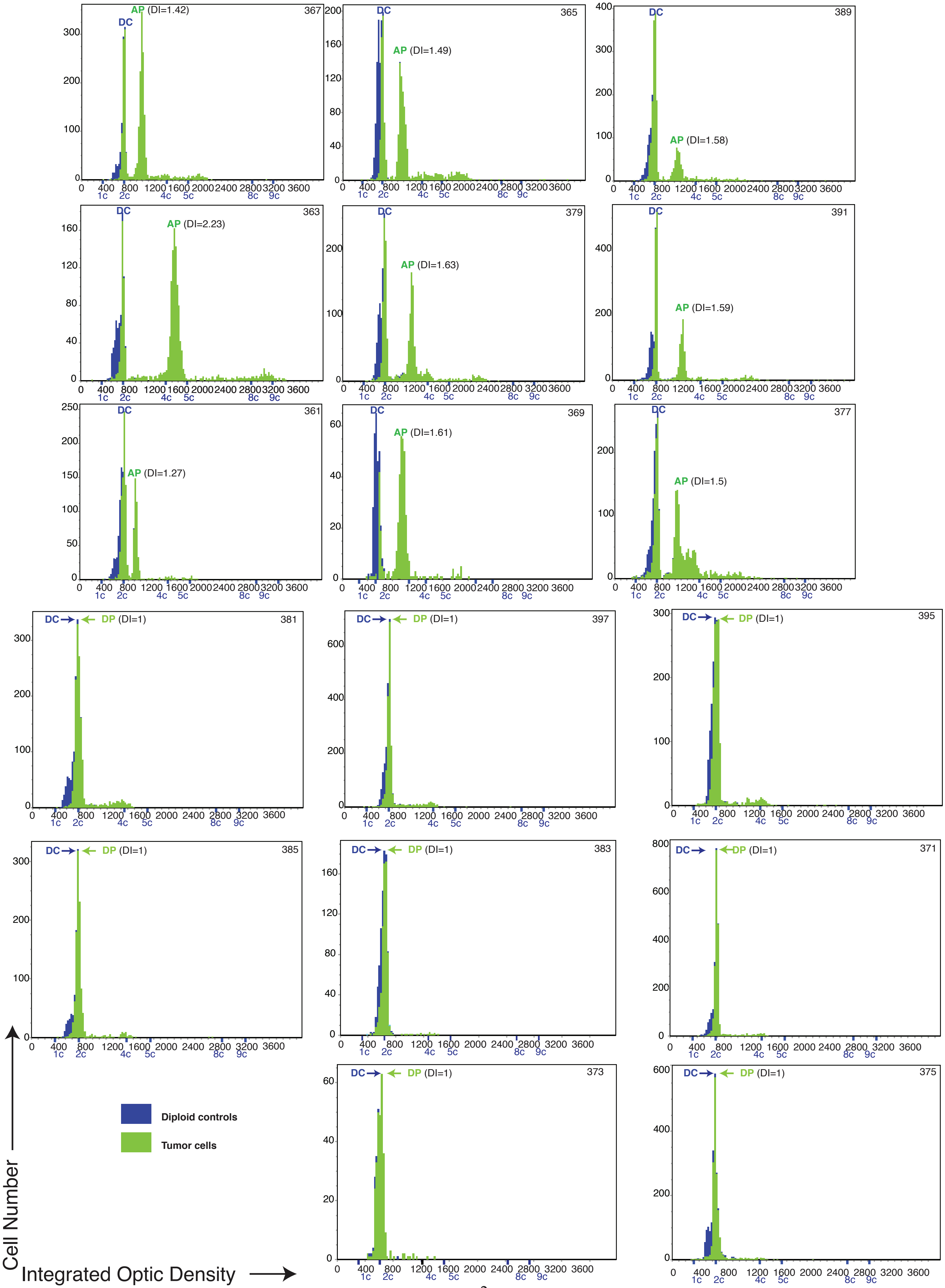

367
DC
AP (DI=1.42)
365
DC
AP (DI=1.49)
389
DC
AP (DI=1.58)
363
DC
AP (DI=2.23)
379
DC
AP (DI=1.63)
391
DC
AP (DI=1.59)
361
DC
AP (DI=1.27)
369
DC
AP (DI=1.61)
377
DC
AP (DI=1.5)
381
DC → ← DP (DI=1)
397
DC → ← DP (DI=1)
395
DC → ← DP (DI=1)
385
DC → ← DP (DI=1)
383
DC → ← DP (DI=1)
371
DC → ← DP (DI=1)
373
DC → ← DP (DI=1)
375
DC → ← DP (DI=1)
0 400 800 1200 1600 2000 2400 2800 3200 3600
1c 2c 4c 5c 8c 9c
Diploid controls
Tumor cells
Cell Number
Integrated Optic Density

Figure S1. Related to Table 1. Ploidy analysis of a cohort of 17 MSS colorectal adenocarcinomas by DNA image cytometry (see Supplemental Experimental Procedures). Histograms show DNA indices (DI) associated to aneuploid and diploid peaks calculated using adjacent fibroblasts, endothelial and infiltrated immune cells as diploid controls. Blue histograms represent diploid controls (fibroblasts, endothelial and infiltrated immune cells); green histograms represent tumor cells. The position of the modal DNA content of the G_1/G_0 diploid controls has been annotated as DC and marks the 2c position. The position of the modal DNA content of the aneuploid tumor cell population has been annotated as AP and the diploid tumor cell population as DP. 1c, 2c, 4c, 5c, 8c and 9c annotation on the horizontal axis mark the theoretical position of the 1N, 2N, 4N, 5N , 8N and 9N populations relative to the diploid controls.

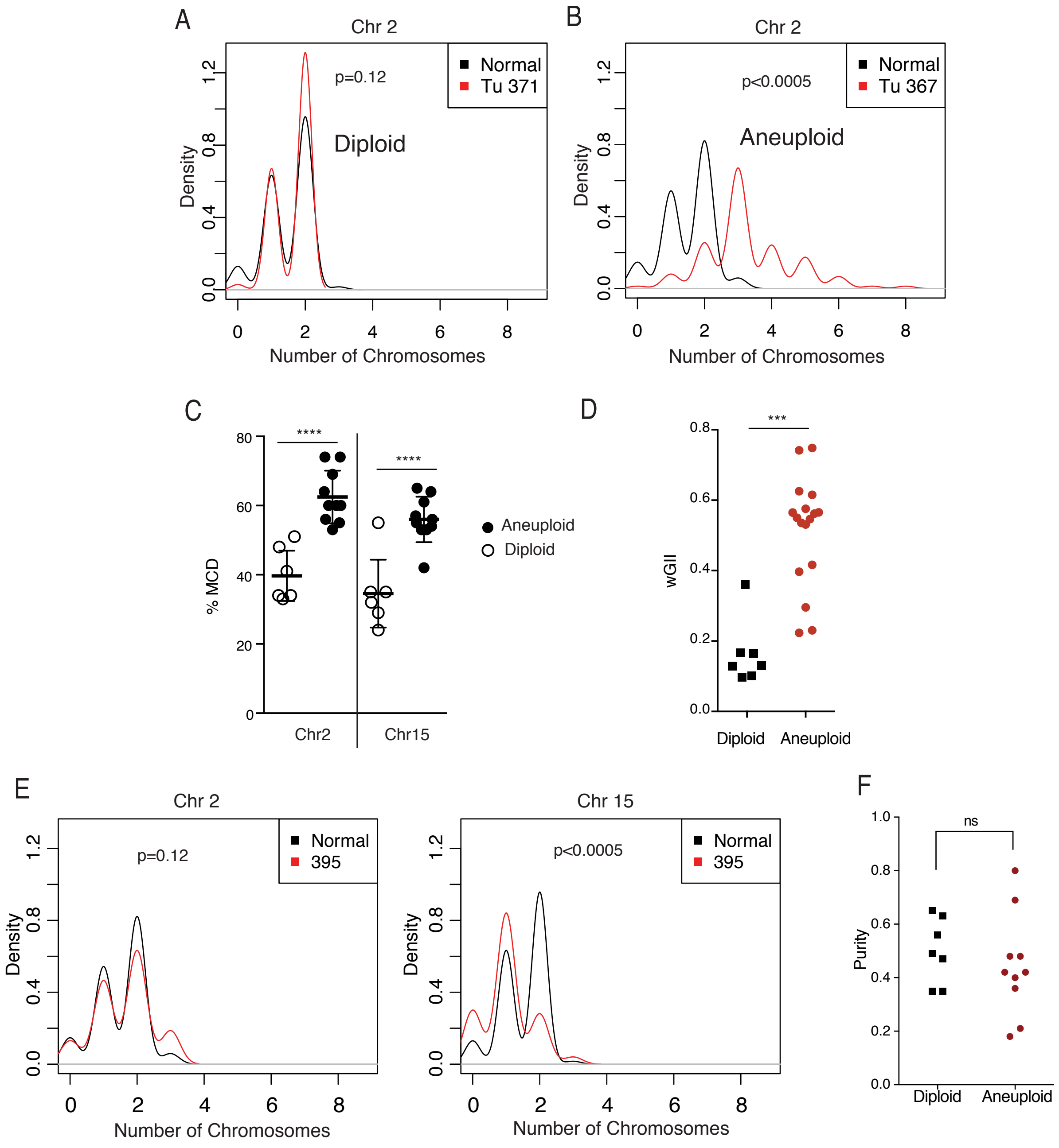

Figure S2. Related to Table 1. (**A -B**) Examples of analysis of chromosome 2 centromeric FISH on a diploid **(A)** and an aneuploid **(B)** colorectal adenocarcinoma. Normal adjacent tissue was used as diploid control (p values calculated by two-tailed Fisher's exact test and Monte Carlo simulations). (**C**) Comparison of the modal centromeric deviation (%MCD) from aneuploid and diploid CRC tumours (chr2 and chr15, p values calculated by unpaired Student's t-test). (**D**) Comparison of the weighted genome instability index (wGII) of aneuploid and diploid CRC tumours (p values calculated by unpaired Student's t-test). (**E**) Centromeric FISH analysis (Chr 2 and 15) of sample 395. (**F**) Difference in tumour cell purity between diploid and aneuploid CRC tumours (p value calculated by unpaired Student's t-test).

Error bars denote SD. ns=not significant, ***p<0.001, ****p<0.0001.

Table S2: Related to Figure 1. Whole-exome sequencing depth and number of variant calls before manual curation in a cohort of 17 MSS colorectal adenocarcinomas and 8 aneuploid cell lines.

Sample (N=Normal)	Median depth (reads)	Non-synonymous calls
**T84**	49	342
**SW480**	43	441
**SW403**	53	598
**SK-CO-1**	45	284
**SW1463**	52	617
**NCI-508**	49	392
**LS123**	54	555
**HT55**	45	1452
**361**	52	38
**362 (N)**	49	
**363**	49	123
**364 (N)**	51	
**365**	37	33
**366 (N)**	45	
**367**	49	51
**368 (N)**	48	
**369**	46	115
**370 (N)**	42	
**371**	43	123
**372 (N)**	48	
**373**	39	100
**374 (N)**	37	
**375**	41	94
**376 (N)**	43	
**377**	54	81
**378 (N)**	51	
**379**	50	84
**380 (N)**	49	
**381**	46	69
**382 (N)**	46	
**383**	57	113
**384 (N)**	47	
**385**	54	102
**386 (N)**	52	
**389**	55	41
**390 (N)**	44	
**391**	63	57
**392 (N)**	53	
**395**	55	65
**396 (N)**	57	
**397**	41	59
**398 (N)**	34	

Table S3. Related to Figure 1. Details of the somatic mutations shown in Figure 1A (note that 371, 397 samples are not shown since they do not display any mutation in genes shown in Figure 1A)

Sample	Chromosome	Position	Germline	Tumour	*Gene Symbol*	Mutation Type
361	chr17	7577157	T	A	*TP53*	Exon 5 -2
	chr3	74347140	C	A	*CNTN3*	S790I
	chrX	32360256	G	C	*DMD*	S1961R
	chr5	112173917	C	T	*APC*	R876X
	chr5	112175216	G	T	*APC*	E1309X
363	chr17	7577547	C	A	*TP53*	G245V
	chr11	118773944	C	T	*BCL9L*	Exon 5 +1
	chr2	168102142	C	T	*XIRP2*	Q1414X
	chr10	26241152	T	C	*MYO3A*	V38A
	chr2	242179190	G	A	*HDLBP*	R813C
	chr18	10485711	G	A	*APCDD1*	A343T
	chr9	125838936	G	C	*RABGAP1*	L701F
	chr3	74350696	G	A	*CNTN3*	P650S
	chr5	112176030	T	A	*APC*	A1580N
365	chr17	7673803	C	T	*TP53*	R273C
367	chr17	7577094	G	A	*TP53*	R282W
	chrX	32632524	A	C	*DMD*	L460V
369	chr3	64619218	C	T	*ADAMTS9*	R702Q
	chrX	117773430	G	A	*DOCK11*	G1345E
	chr16	72991587	G	A	*ZFHX3*	R820C
	chr2	128877943	G	T	*UGGT1*	D272Y
	chr12	25398284	C	A	*KRAS*	G12V
	chr5	112174631	C	T	*APC*	R1114X
373	chr5	112164586	C	T	*APC*	R554X
375	chr5	112175706	T	-	*APC*	frameshift
377	chr17	7578176	G	T	*TP53*	Exon 3 +1
	chr10	26377153	G	T	*MYO3A*	E461X
	chr13	101881748	G	A	*NALCN*	P541L
	chr13	77862394	T	C	*MYCBP2*	I128V
	chr2	171258135	A	G	*MYO3B*	Y688C
	chr13	25353803	A	T	*RNF17*	Exon 5 -2
	chr16	72984723	G	A	*ZFHX3*	T954M
	chr1	202702954	C	T	*JARID1B*	A1162T
	chr12	25398281	C	T	*KRAS*	G13D
	chr5	112174268	A	T	*APC*	K993X
	chr5	112116592	C	T	*APC*	R213X
379	chr11	118772315	G	A	*BCL9L*	Q713X
	chr20	34021715	A	G	*GDF5*	C500R
	chr13	101763474	A	G	*NALCN*	Exon 20 +2
	chr15	84611752	G	A	*ADAMTSL3*	C803Y
	chr6	51923374	C	T	*PKHD1*	G420D
	chr12	25398281	C	T	*KRAS*	G13D
381	chr12	25398284	C	A	*KRAS*	G12V
	chr5	112174182	-	A	*APC*	frameshift
383	chr5	112128191	C	T	*APC*	R232X
	chr12	25398284	C	T	*KRAS*	G12D
385	chr4	153249384	C	A	*FBXW7*	R347L
	chr12	25398281	C	T	*KRAS*	G13D
	chr5	112176027	T	-	*APC*	frameshift
	chr5	112173349	-	C	*APC*	frameshift
389	chr17	7577094	G	A	*TP53*	H282Y
	chr6	10907840	G	T	*SYCP2L*	E248X
	chr12	25398284	C	T	*KRAS*	G12D

391	chr17	7571720	G	A	*TP53*	Exon 8 +1
	chr1	26366306	G	A	*SLC30A2*	T312M
	chr5	112175212	AAAAG	-	*APC*	frameshift
395	chr17	7578406	C	T	*TP53*	R175H
	chr5	13894933	G	T	*DNAH5*	Exon 16 +2
	chr5	112175212	AAAAG	-	*APC*	frameshift
SW403	chr17	7579536	C	A	*TP53*	E51X
	chr2	168106261	G	T	*XIRP2*	E2787X
	chr3	64672354	G	A	*ADAMTS9*	Q136X
	chr10	26459447	A	T	*MYO3A*	K1126M
	chr1	204425158	G	T	*PIK3C2B*	T590N
	chr1	202729562	C	A	*JARID1B*	C353F
	chr6	51613167	C	T	*PKHD1*	V3083M
	chr2	128910379	G	T	*UGCGL1*	D656Y
	chrX	153587463	C	T	*FLNA*	G1455R
	chr20	50048607	C	A	*NFATC2*	D907Y
	chr5	112174884	C	T	*APC*	S1198F
	chr5	112175124	C	A	*APC*	S1278X
HT55	chr17	7578211	C	A	*TP53*	R213L
	chr2	242187700	T	A	*HDLBP*	K526X
	chr13	101844385	C	A	*NALCN*	Q549H
	chr1	204426163	A	C	*PIK3C2B*	Exon 11 +2
	chr2	171399474	A	T	*MYO3B*	K1212X
	chrX	117805037	G	T	*DOCK11*	E1710X
	chr13	101844385	C	A	*NALCN*	Q549H
	chr4	122765089	T	A	*BBS7*	D433V
	chrX	91132857	A	T	*PCDH11X*	I540F
	chrX	32404536	A	T	*DMD*	V1522E
	chrX	153581290	A	T	*FLNA*	Y2069N
	chr4	153303466	T	A	*FBXW7*	T8S
	chr5	112174682	C	T	*APC*	Q1131X
	chr5	112175198	C	T	*APC*	Q130X3
	chr5	112175680	A	T	*APC*	R1463S
NCI-H508	chr17	7577120	C	T	*TP53*	R273H
	chr10	26417463	A	G	*MYO3A*	E753G
	chr20	34022560	C	A	*GDF5*	R218M
SK-CO-1	chr3	64641310	G	T	*ADAMTS9*	Q371K
	chr2	242195813	T	A	*HDLBP*	D220V
	chr18	10485730	G	A	*APCDD1*	R349H
	chr18	5397160	A	G	*EPB41L3*	V913A
	chr9	125752338	A	C	*RABGAP1*	S185R
	chr15	84651057	G	A	*ADAMTSL3*	E893K
	chr4	122782666	T	C	*BBS7*	K112E
	chrX	91134258	G	A	*PCDH11X*	V1007I
	chr12	49087306	G	A	*CCNT1*	S564F
	chr12	25398284	C	A	*KRAS*	G12V
	chr5	112838859	T	-	*APC*	frameshift
T84	chr20	34022340	C	T	*GDF5*	W291X
	chr1	204423833	G	A	*PIK3C2B*	S677F
	chr16	72821983	C	G	*ZFHX3*	A3398P
	chr21	41550978	C	A	*DSCAM*	Q941H
	chr12	49089589	T	A	*CCNT1*	I244F
	chr12	25398281	C	T	*KRAS*	G13D
	chr5	112840058	T	-	*APC*	frameshift
LS123	chr17	7578406	C	T	*TP53*	R175H
	chr2	168104014	G	A	*XIRP2*	A2038T
	chr3	64617175	C	T	*ADAMTS9*	R782Q
	chr3	74316452	C	G	*CNTN3*	A928P

	chr21	41416190	C	T	*DSCAM*	R1733Q
	chr12	25398285	C	T	*KRAS*	G12S
	chr5	112170777	C	T	*APC*	Q625X
	chr5	112175639	C	T	*APC*	R1450X
SW948	chr6	10898308	C	T	*SYCP2L*	S134L
	chr13	77670484	T	G	*MYCBP2*	K3268T
	chr13	77765922	T	C	*MYCBP2*	E1283G
	chr1	26371527	A	T	*SLC30A2*	S78T
	chr20	50049005	G	A	*NFATC2*	P774L
	chr12	25380276	T	A	*KRAS*	Q61L
	chr5	112174631	C	T	*APC*	R1114X
	chr5	112175576	C	T	*APC*	Q1429X
SW1463	chr17	7577538	C	T	*TP53*	R248Q
	chr11	118772306	G	A	*BCL9L*	R716X
	chr2	168100943	T	A	*XIRP2*	V1014E
	chr5	13830149	G	A	*DNAH5*	L2079F
	chr3	74349047	C	A	*CNTN3*	S713I
	chrx	91132792	G	A	*PCDH11X*	R518H
	chr4	153247366	C	T	*FBXW7*	R361Q
	chr12	25398285	C	A	*KRAS*	G12C
	chr5	112176756	TG	-	*APC*	frameshift

Table S4: Related to Figure 1. Missense *BCL9L* mutations and Polyphen scores of predicted functional impact in 4 MSS CRC cohorts (TCGA, Giannakis et al., 2016 and 2 validation cohorts of 30 and 26 samples).

***BCL9L* mutation**	**Polyphen score**
E676G	Probably damaging
S700N	Benign
P842S	Probably damaging
Q1381R	Probably damaging
P1077R	Probably damaging
P1197A	Benign
A578V	Benign
L1064P	Benign
R409W (3)	Probably damaging
T1100I	Probably damaging
P356L	Benign
R581W	Benign
S410F	Probably damaging
V828	Benign
A1009C	Probably damaging

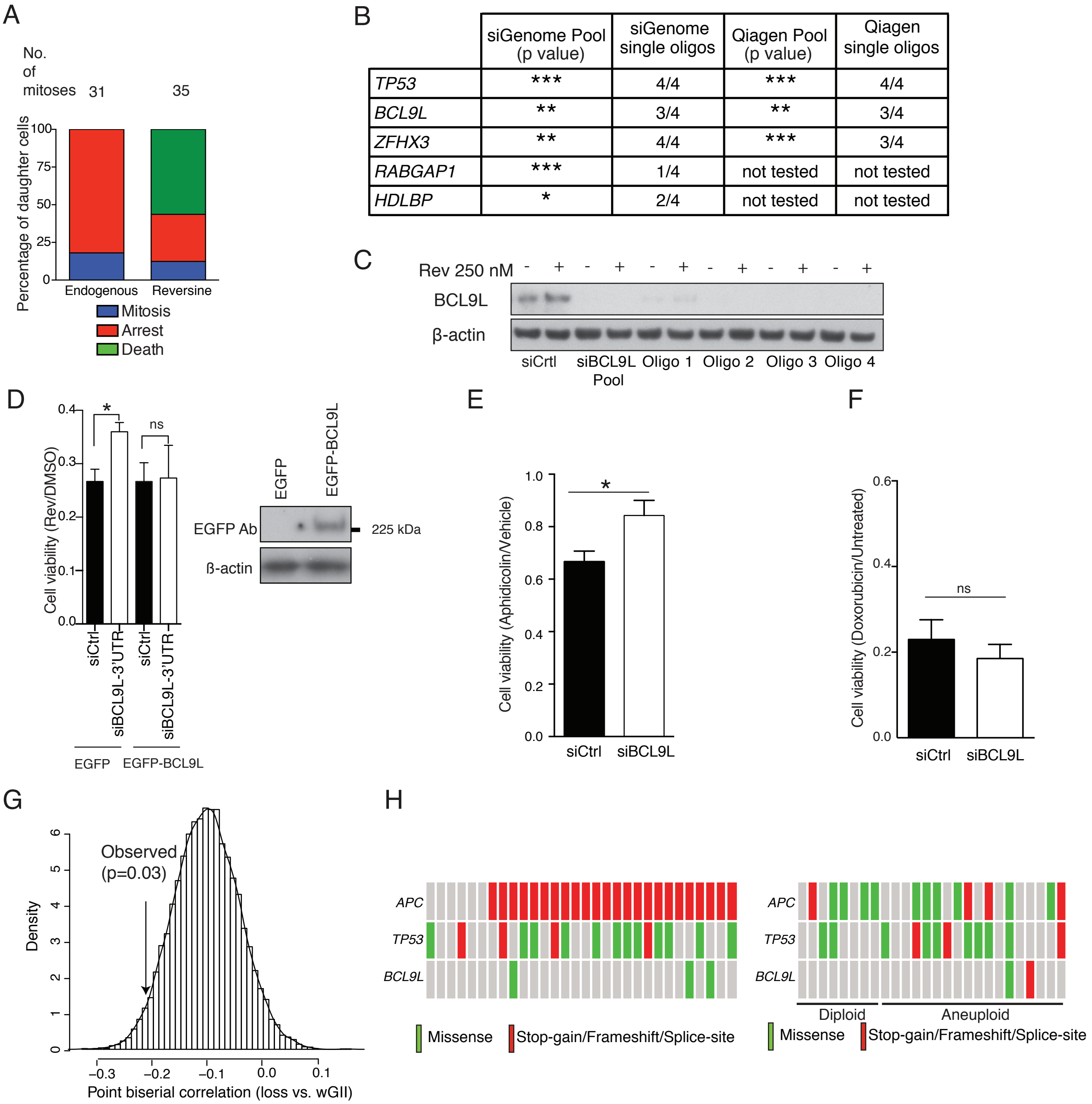

	siGenome Pool (p value)	siGenome single oligos	Qiagen Pool (p value)	Qiagen single oligos
TP53	***	4/4	***	4/4
BCL9L	**	3/4	**	3/4
ZFHX3	**	4/4	***	3/4
RABGAP1	***	1/4	not tested	not tested
HDLBP	*	2/4	not tested	not tested

Figure S3. Related to Figure 1. **(A)** Fate of daughter cells after endogenous or reversine-induced segregation error studied by live-cell imaging of HCT-116 cells expressing H2B-RFP. **(B)** Summary of the effect of TP53, BCL9L, RABGAP1, HDLBP and ZFHX3 knock-down on cell survival upon 250 nM with pools of 4 and indivudual siRNA duplexes (n=3, p value calculated by paired Student's t-test). **(C)** Western blot analysis of the BCL9L knock-down efficiency following transfection of an siRNA pool or transfection of individual duplexes (Qiagen). **(D)** Viability of HCT-116 cells co-transfecting a BCL9L-EGFP construct and a single siRNA duplex targeting the 3'-UTR region of *BCL9L* mRNA as indicated in the presence and absence of 250 nM reversine. (siRNA duplex shown in Supplemental Experimental Procedures, 72 hr, n=3, p value calculated by Student's t-test). **(E)** Effect of BCL9L depletion on survival upon aphidicolin treatment (200 nM, 4 days, n=3, p value calculated by paired Student's t-test) **(F)** Effect of BCL9L depletion on survival upon doxorubicin treatment (100 nM, 3 days, n=3, p value calculated by paired Student's t-test). **(G)** Computational permutation analysis showing a correlation between wGII and *BCL9L* copy-number loss in MSS CRC (TCGA, see Supplemental Experimental Procedures). **(H)** Mutational status of *APC*, *TP53* and *BCL9L* determined using Ion Torrent targeted sequencing in a cohort of 30 MSS CRC tumours (left) and a cohort of 26 MSS CRC tumours (right) of known ploidy (analyzed by DNA image cytometry).

Error bars denote SD. ns=not significant, *p<0.05, **p<0.001***p<0.001, ****p<0.0001.

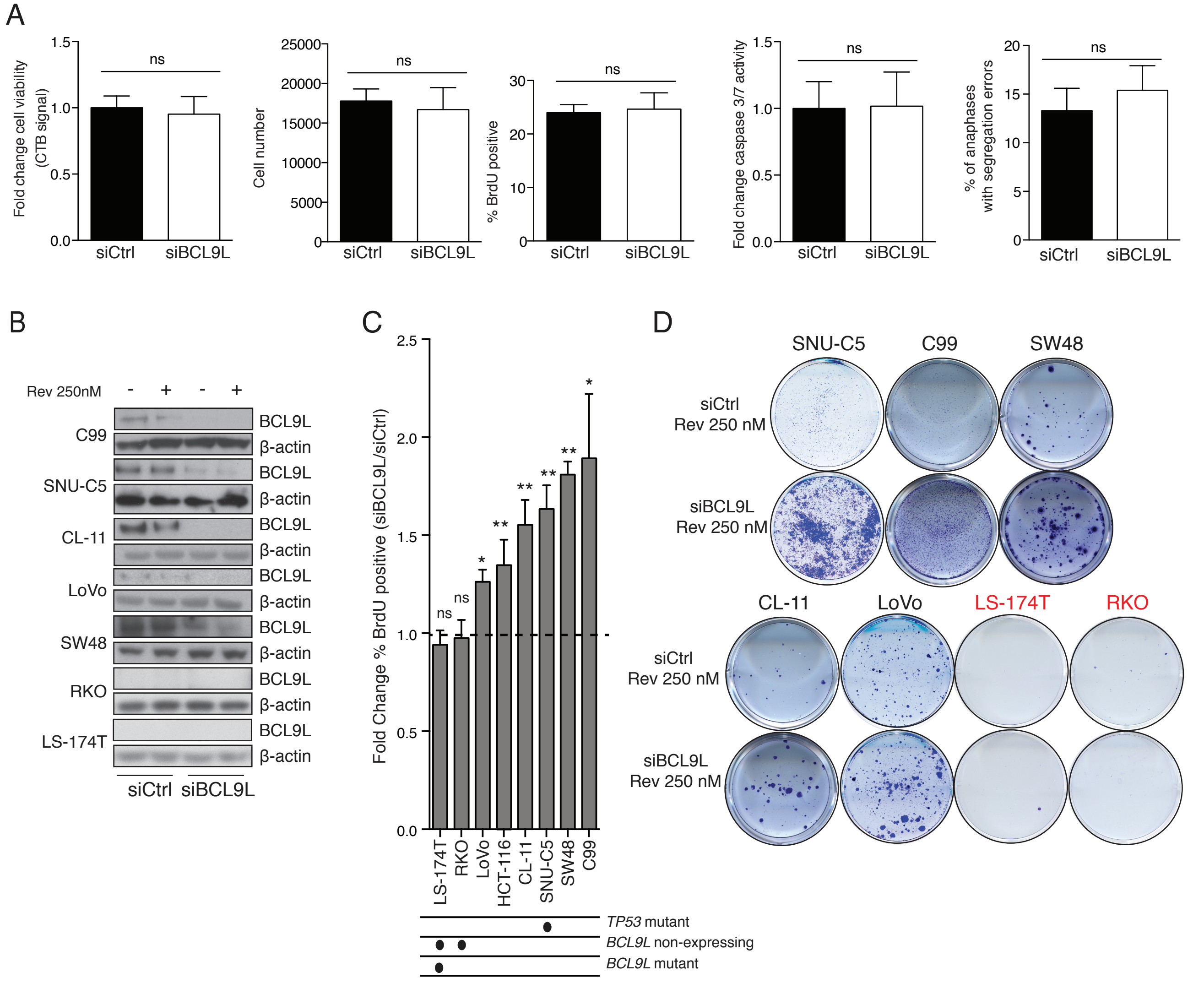

Figure S4. Related to Figure 2. (**A**) Basal effect of BCL9L knock-down on cell viability, cell number, BrdU incorporation, caspase 3/7 activity and endogenous segregation errors in the absence of reversine (n=3, p values calculated by paired Student's t-test). (**B)** Western-blot analysis of BCL9L protein levels in 7 near-diploid cell lines. Effect of 3 day reversine treatment and BCL9L knock-down are shown. (**C**) Effect of BCL9L depletion on the fraction of BrdU-incorporating cells in 8 reversine-treated near-diploid cell lines (n=3, no changes detected in untreated controls, p value calculated by paired Student's t-test). Cells were treated with 250 nM reversine for 3 days followed by 1 hr BrdU pulse. *BCL9L* and *TP53* mutation status is shown at the bottom. (**D)** Colony forming efficiency of 7 near-diploid CRC cell lines after BCL9L siRNA transfection and reversine treatment. After 72 hr of siRNA transfection, cells were plated in serial dilutions in the presence and absence of 250 nM reversine for 5 days and grown until appropriate colony size was achieved. LS-174T and RKO do not express BCL9L and accordingly do not benefit from BCL9L knock-down.

Error bars denote SD. ns=not significant, *p<0.05, **p<0.001.

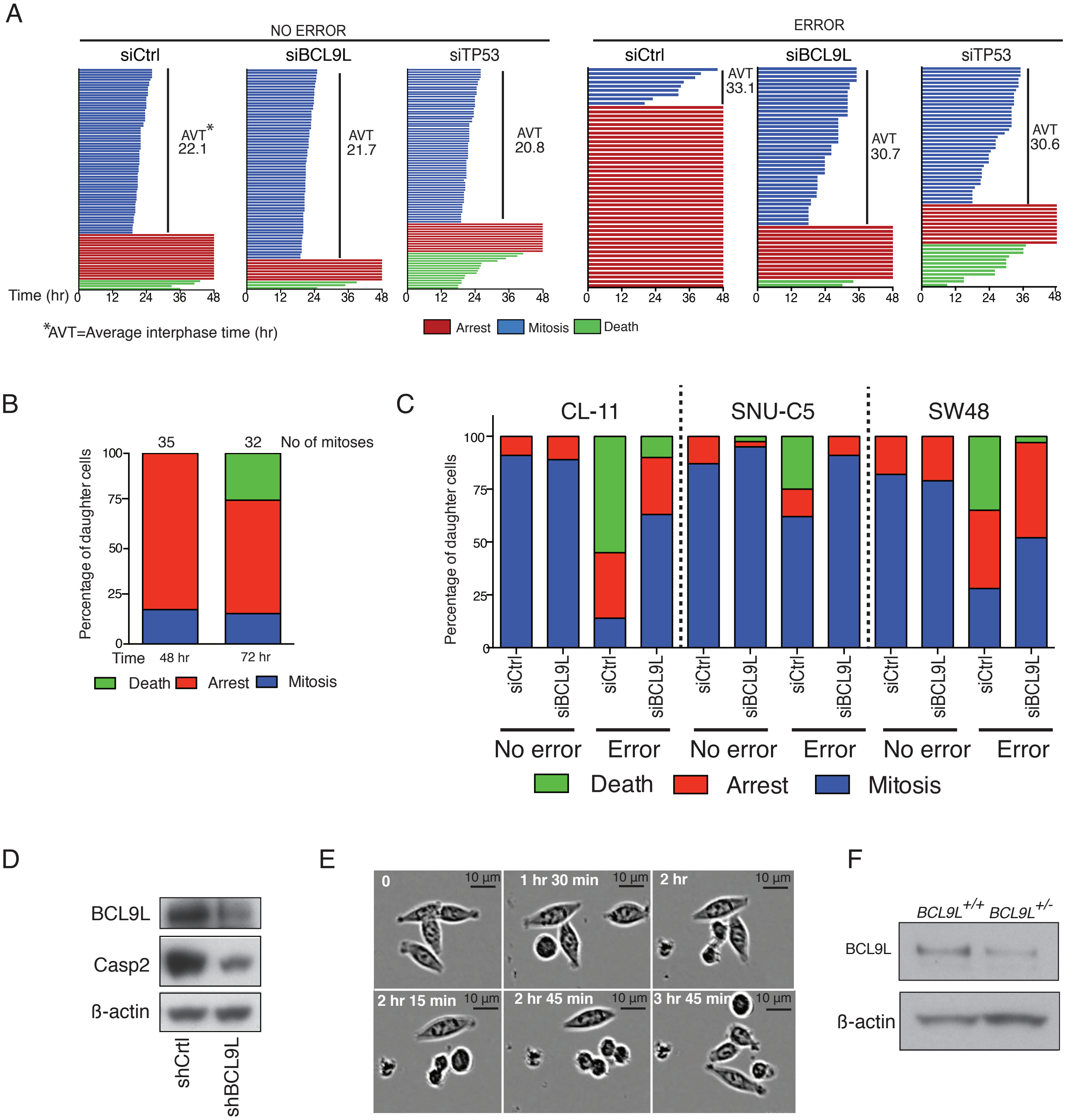

Figure S5. Related to Figure 2 and 3. (**A**) Single cell analysis of the effect of BCL9L and TP53 knock-down on post-mitotic daughter cell fate after normal mitosis or mitosis with endogenous segregation error (Time=0 marks the time of the first mitosis) (**B**) Post-mitotic daughter cell fate of HCT-116 after mitosis with chromosome segregation error. Live cell imaging was carried out for 48 or 72 hr. (**C**) Post-mitotic daughter cell fate of 3 near-diploid cell lines after a normal mitosis or with an endogenous chromosome segregation error. A minimum of 30 mitoses cells were analyzed. (**D**) Western-blot analysis of BCL9L and caspase-2 expression in HCT-116 cells stably expressing a *BCL9L* shRNA. (**E**) Representative images of mitosis in HCT-116 cells in reversine obtained by live-cell brightfield microscopy. All mitosis analyzed underwent normal cytokinesis and divided. (**F**) Levels of BCL9L protein in a *BCL9L*-WT clone and a *BCL9L*-mutant clone with a heterozygous 5 bp deletion downstream the HD3 domain as shown in 3A.

Movie S1: Two anaphases at 1 hr 33 min and 3 hr 33 min with segregation errors. Daughter cells arrest or die. Related to Figure 2

Movie S2: Anaphase with chromosomal bridge at 3 hr 15 min. Daughter cells divide and arrest. Related to Figure 2

Movie S3: Anaphase with lagging chromosome at 51 min. Both daughter cells divide. Related to Figure 2

Movie S4: Normal mitosis at 42 min. Both daughter cells divide. Related to Figure 2

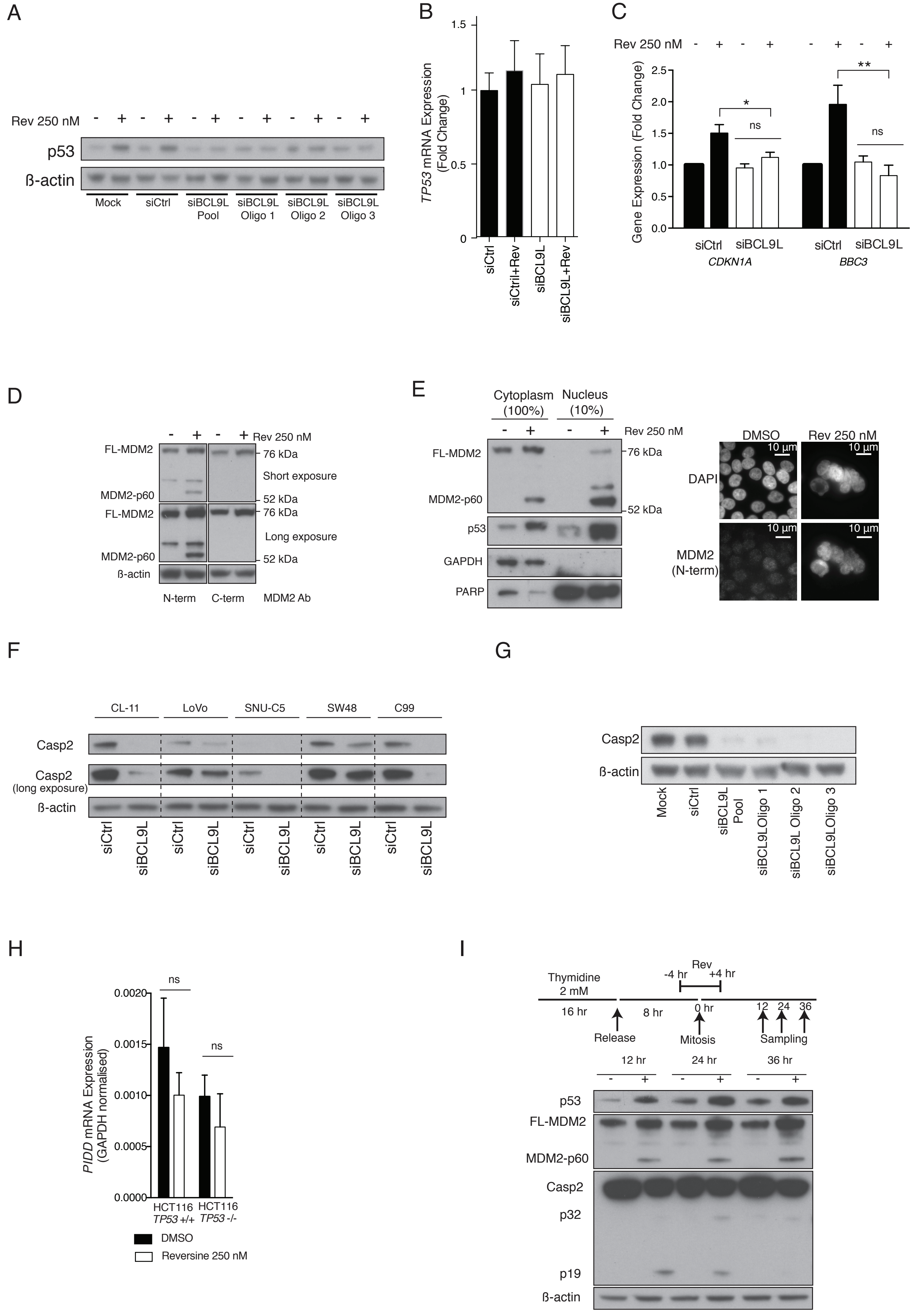
A
Rev 250 nM
p53
ß-actin
Mock
siCtrl
siBCL9L Pool
siBCL9L Oligo 1
siBCL9L Oligo 2
siBCL9L Oligo 3
B
TP53 mRNA Expression (Fold Change)
siCtrl
siCtrl+Rev
siBCL9L
siBCL9L+Rev
C
Gene Expression (Fold Change)
ns
CDKN1A
BBC3
D
FL-MDM2
MDM2-p60
76 kDa
52 kDa
Short exposure
Long exposure
N-term
C-term
MDM2 Ab
E
Cytoplasm (100%)
Nucleus (10%)
GAPDH
PARP
DMSO
Rev 250 nM
10 µm
DAPI
MDM2 (N-term)
F
CL-11
LoVo
SNU-C5
SW48
C99
Casp2
Casp2 (long exposure)
G
H
PIDD mRNA Expression (GAPDH normalised)
HCT116 TP53 +/+
HCT116 TP53 -/-
DMSO
Reversine 250 nM
I
Thymidine 2 mM
16 hr
Release
8 hr
Rev
-4 hr
+4 hr
0 hr
Mitosis
Sampling
12 hr
24 hr
36 hr
p32
p19

Figure S6. Related to Figure 5. (**A)** Reversine mediated induction of p53 in HCT-116 and effect of BCL9L knock-down with a pool of 3 siRNAs (Qiagen) and 3 individual single siRNAs (components of the pool) in HCT-116. (**B)** qPCR analysis of *TP53* mRNA expression in isogenic *TP53*-WT and null HCT-116 cells following reversine treatment and BCL9L depletion (n=3). (**C)** qPCR analysis of the p53 transcriptional targets *CDKN1A* (p21) and *BBC3* (PUMA) in HCT-116 cells and the effect of reversine and BCL9L knock-down (n=3, p values calculated by unpaired Student's t-test). (**D)** Immunodetection of MDM2 with antibodies against the N-terminal domain (left) or C-terminal domain (right) respectively. (**E)** Subcellular localization of full length-MDM2 and MDM2-p60 and p53 assessed by cellular fractionation and immunofluorescence (Note that nuclear protein represents 10% of cells relative to cytoplasmic). (**F)** Effect of BCL9L knock-down on caspase-2 expression in 5 near-diploid cell lines (2 exposures shown). (**G)** Effect of BCL9L depletion on caspase-2 expression with a pool of 3 siRNA oligonucleotides (Qiagen) and 3 individual siRNA oligonucleotides (components of the pool, marked in Supplemental Experimental Procedures). (**H)** Analysis of *PIDD* mRNA expression level by qPCR analysis following reversine treatment in HCT-116 *TP53*-WT and null cells (n=3, p values calculated by unpaired Student's t-test). (**I)** Time-course analysis of p53 accumulation, MDM2 cleavage and caspase-2 activation in synchronized HCT-116 cells transiently exposed to reversine during a single mitosis. Cells were synchronized at the G1-S transition with 2 mM thymidine for 16 hr, and released in fresh media to allow cell cycle progression. 250 nM reversine was added 4 hr before the estimated time for mitosis (8 hr after thymidine washout) and removed 4 hr after the estimated time for mitosis such that cells are only exposed to reversine during a single mitosis. Samples were collected 12, 24 and 36 hr later and lysed for western blot analysis.

Error bars denote SD. ns=not significant, *p<0.05, **p<0.001.

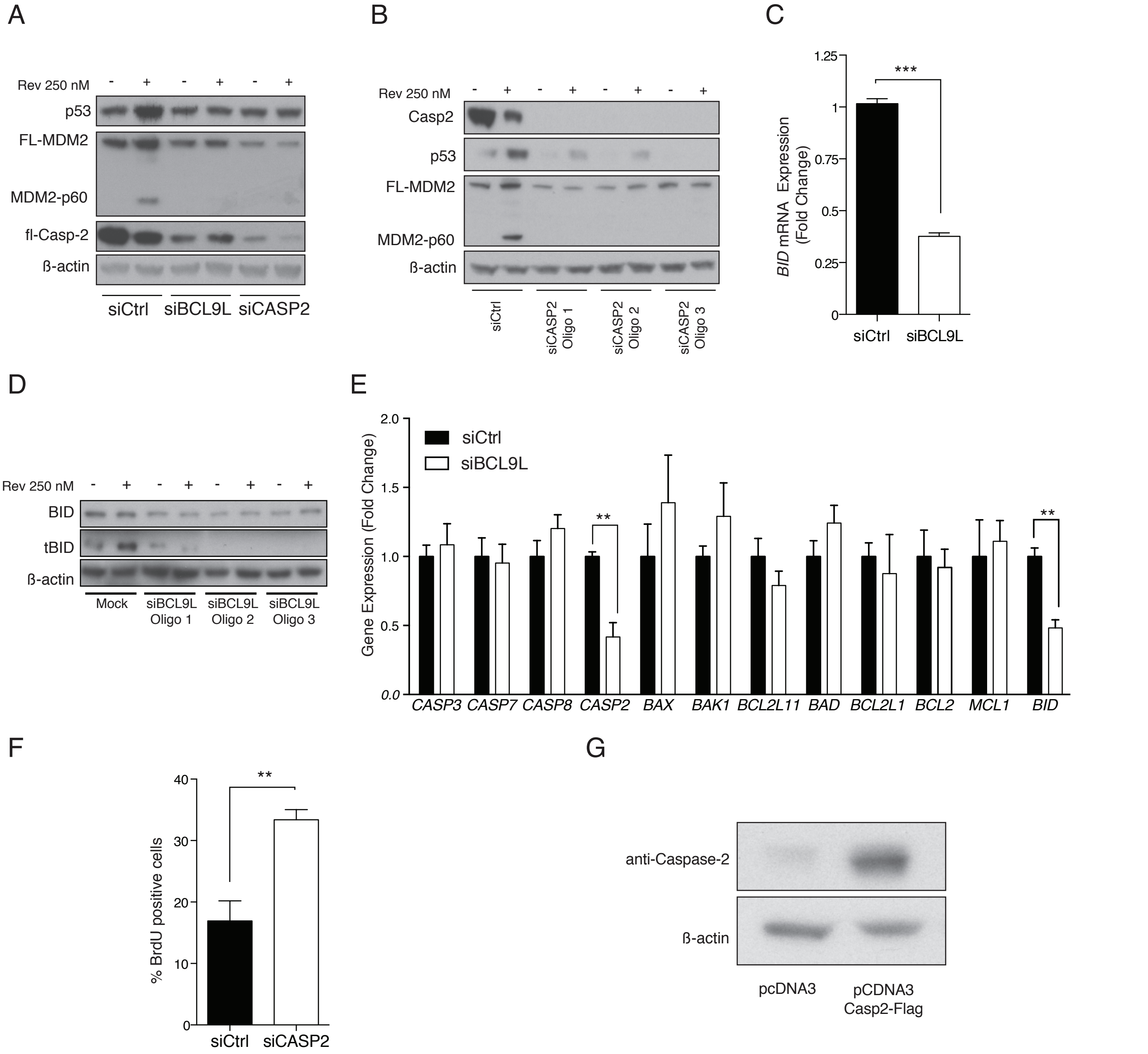

Figure S7. Related to Figure 5. (**A**) Effect of BCL9L and caspase-2 depletion on MDM2 cleavage and p53 stabilization following reversine treatment of HCT-116 cells. (**B**) Effect of caspase-2 knock-down on p53 stabilization and MDM2 cleavage with 3 different siRNA duplexes, and treated with reversine where indicated (Qiagen siRNAs used identified in Supplemental Experimental Procedures). (**C**) Analysis of *BID* mRNA expression in *TP53*-null HCT-116 by qPCR (n=3, p values calculated by unpaired Student's t-test). (**D**) Effect of *BCL9L* silencing with 3 different siRNA sequences on *BID* expression and cleavage in *TP53*-null HCT-116 determined by western blot (same oligonucleotides as the ones used in S6A and S6G). (**E**) qPCR analysis of mRNA expression of a selection of various initiator and effector caspases and components of the BCL2 family of mitochondrial apoptotic proteins (Fold change relative to siRNA control, n=3, p values calculated by unpaired Student's t-test). (**F**) Effect of caspase-2 depletion on cell proliferation as determined by the fraction BrdU-incorporating cells in 250 nM reversine treated HCT-116 cells (n=3, p values calculated by paired Student's t-test). (**G**) Caspase-2 protein levels in pcDNA3-Caspase2-Flag transfected HCT-116.

Error bars denote SD. **p<0.001, ***p<0.0001.

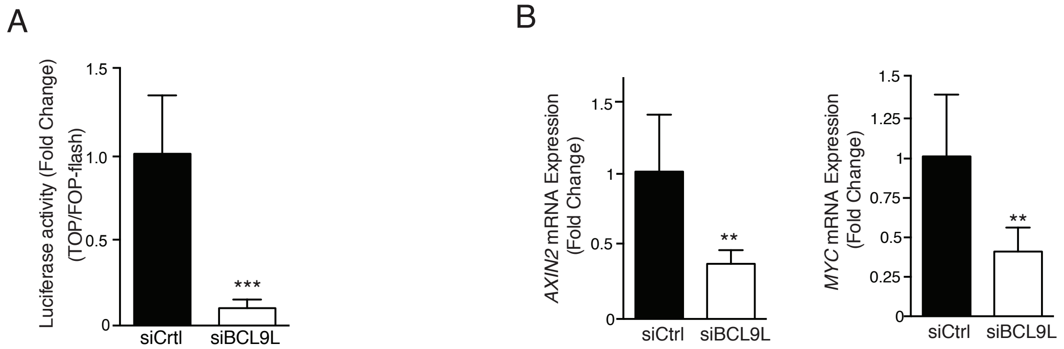

Figure S8. Related to Figure 6. (**A)** Effect of BCL9L knock-down on Wnt signaling (TOP/FOP-Flash luciferase reporter). (**B**) Gene expression analysis of the effect of BCL9L depletion on the Wnt signaling targets *AXIN2* and *MYC*.

p values calculated by unpaired Student's t-test. Error bars denote SD. **p<0.001, ***p<0.0001.

SUPPLEMENTAL EXPERIMENTAL PROCEDURES

Patient samples, whole-exome sequencing, Ion-Torrent targeted sequencing and SNP array

Tissue collection was approved by an ethics committee (NRES Committee South Central-Oxford B, REC reference 05/Q1605/66), and all individuals included in this study had provided written informed consent for the analysis presented.

For whole exome sequencing, DNA was extracted from fresh frozen colorectal adenocarcinomas and adjacent normal colon. Exome enrichment was carried out with SureSelect 38Mb+. Exome sequencing was performed on an Illumina GAIIx in 72 bp paired end mode. FastQ files were aligned to hg19 reference sequence using bwa 0-5.9r16 with a seed length of 72 bases and all other settings left as default (in particular up to three mismatches were allowed per read). Sam/Bam files associated with the same sample were merged and converted to pileup files using samtools 0.1.16. Point mutations were identified from these pileup files using in-house scripts, which applied a series of thresholds. Variant calls were validated by Sanger sequencing in tumour and normal colon DNA samples.

For Ion Torrent analysis we designed oligonucleotide libraries for a panel of genes including *APC*, *TP53*, *CTNNB1*, *BCL9L* and *BCL9*. Putative variants were targeted for validation using the Ion Torrent platform, with alignment of raw reads carried out on the Torrent Server using Tmap, with a minimum detection frequency of required before the validation was considered to be successful. Somatic status of mutations was validated by Sanger sequencing in tumour and normal colon DNA samples.

For SNP array analysis of tumour samples, DNA was run on Human 610 Quad v1 or Human CytoSNP12 v2.1 arrays in accordance with the Illumina Infinium assays manual protocol (Illumina). Genome Studio software (Illumina) was used to retrieve LogR Ratios and B allele frequencies and calls for copy number changes and LOH status were obtained with OncoSNPv2 software.

For multi-region SNP array of xenografts, we used the Affymetrix CytoScan HD SNP array platform. All analysis was performed in the R statistical environment version 3.2.1. Arrays were processed along with a batch of 15 arrays of normal blood acquired from GEO, GSE53799, using the Aroma Affymetrix CRMAv2 algorithm, and the B-allele fraction (BAF) was adjusted using the CalMaTe algorithm. Tumor LogR data was segmented using the PCF algorithm. Thresholds for calling copy number gain and loss was determined from analysis of the distribution of segmented LogR scores, and set at -0.3 for loss and 0.15 for gain on the uncentered distribution.

Ploidy analysis (DNA Image Cytometry)

Image Cytometry analysis was performed according to methods described in Dunn et al. (2010). In brief, 50 μm sections were cut from FFPE blocks, dewaxed in xylene, rehydrated in ethanol series and digested with proteinase XXIV (Sigma-Aldrich). Sample was washed in PBS, filtered with a 40 μm strainer and resuspended in PBS. The nuclear suspension was spun onto Superfrost slides Plus (positively charged, VWR), dried for 1 hr placed in HCl 5 M for 1 hr and stained with Feulgen-Schiff using published methodology (Bocking et al., 1995). Integrated optical density was measured using a Fairfield DNA Ploidy System. A minimum of 600 nuclei were analyzed, sorted into 4 separate categories (cancer cells, lymphocytes, plasma cells and fibroblasts). Lymphocytes were used as control to determine diploid peak position. Histograms were finally analyzed according to European Society for Analytical Cellular Pathology (Bocking et al., 1995, Haroske at al., 2001).

FISH (FFPE specimens)

5 μm sections were dewaxed in xylene, rehydrated in ethanol series, permeabalized in 1 M sodium thiocyanate at 80°C for 10 min and washed 2 times 10 min in PBS. Samples were then digested in 0.4% pepsin in 0.1 M HCl at 37°C for 10 min followed by quenching with 0.2% glycine in PBS for 5 min and 3 washes in PBS over 15 min. Samples were then fixed in 4% PFA for 10 min, washed 3 times in PBS over 15 min and dehydrated in ethanol series. 1 μl of chromosomal centromeric probes (CEP2 and CEP15, Abbot Molecular Inc.) was diluted in 10 μl of provided hybridization buffer, placed onto the sample, covered with coverslip, sealed with rubber solution glue, placed at 95°C for 10 min and transferred overnight to a 37°C humidified incubator. Slides were then washed 3 times with 0.5xSSC (20xSSC composition is 17.5% NaCl, 8.8% tri-sodium citrate) over 15 min at 37°C and 3 times with PBS over 15 min at room temperature. Finally, slides were then counterstained with DAPI, dehydrated in ethanol series and mounted in fluorescence compatible medium.

FISH (Cell lines)

Cells were grown on Poly-Lysine coated glass slides with required culture medium, washed with PBS, placed in 37°C hypotonic solution (0.4% KCl and 0.4% sodium citrate) for 7 min and fixed in 3:1 methanol/acetic acid at -20°C for 15 min. Cells fixed onto slides were denatured in 70% formamide,

2X SSC at 70°C for 90 seconds and then transferred to 70% ethanol at -20°C. CEP probes (CEP 1, 2, 8, 15, Abbot Molecular Inc.) were denatured at 95°C then placed onto slides, covered with coverslips and sealed with rubber solution glue. Hybridization was performed in a humidified incubator overnight at in 37°C. Slides were then washed 3 times with 50% formamide, 2X SSC over 15 min at 42°C, 3 times with 2x SSC over 15 min at 42°C, once with 4x SSC, 0.02% Tween for 5 min and finally once in PBS. Slides were counterstained with DAPI, dehydrated in ethanol series, and mounted in with fluorescence compatible media.

Metaphase spreads

Cells were treated with 10 µl/mL Colcemid (GIBCO) for 90 min, trypsinised and allowed to swell in hypotonic solution (0.4% KCl and 0.4% sodium citrate) for 7 min at 37°C. Cells were then spun down and the pellet resuspended in methanol/acetic acid (3:1). The suspension of fixed cells was dropped onto glass slides to allow cell breakage and the existence of chromosome spreads confirmed under the microscope. Metaphase spreads were stained with a human pan-centromeric FISH (Kreatech, Leica) probe with enzymatic treatment as described by the manufacturer.

TCGA mutation and copy-number data

All mutation data were downloaded from the Broad Institute MAF dashboard (https://confluence.broadinstitute.org/display/GDAC/MAF+Dashboard). Mutations were filtered to ensure that each variant had at least five tumor reads and coverage of ≥30.

Affymetrix SNP 6.0 arrays for TCGA for Colorectal Adenocarcinoma were obtained from TCGA (https://tcga-data.nci.nih.gov/tcga). Allele-specific integer copy numbers and purity estimates, were obtained using ASCAT.

Cell lines, siRNA, shRNA transfections and DNA constructs

Cell lines were provided by Cell Services at The Francis Crick Institute or by collaborators, mycoplasma tested and authenticated by STR profiling and comparison against published profiles.

siRNA pools were obtained from Qiagen (GeneSolution) and Dharmacon (siGENOME) (sequences provided below). Tranfections were carried out to a final concentration of 40 nM with Lipofectamine RNAiMAX transfection reagent (Life Technologies) to a final 1/400 dilution.

For stable silencing of *BCL9L* we used a GIPZ lentiviral construct (Clone V3LHS_368414, target sequence AGCAGATGAACATGATGAT, ThermoFisher).

TOP/FLOP-Flash luciferase reporter constructs were purchased from Millipore. *BCL9L* cDNA was cloned into a pEGFP-N1 for EGFP C-terminal fusion (ClonTech). pcDNA3-Casp2-Flag construct was obtained from Addgene. Constructs were transfected using Lipofectamine-2000 (Life Tecnologies) according to manufacturers instructions.

siRNA sequences

siGENOME

Gene Symbol	Accession Number	Target Sequence
ADAMTS9	NM_182920	CGACAAAUGUGAUACCUUA<br>GGACGUAGAAUGAAAUUUA<br>GGAAAGCACUGCAAGUAUG<br>GGACACAAAUGAUAUCUGU
ADAMTSL3	NM_207517	GGACAGAGGUCAUCAAUAU<br>GAACAUAAUCCUUGGACUG<br>ACAAUCCGAUGUCCUGUAA<br>GUAGAGGAAUCCAUGCAUG
APCDD1	NM_153000	GCUCAAACAUCUCCACAAU<br>CUUCAAGGCCUACCAAUUU<br>CAAACUACCUCACACGGAG<br>GCCCAGAGUUCAUCACAAG
BCL9L	NM_182557	GAACAGCAGUGGCGUGAUG<br>GCUGAUGCCUUCACAGUUU

		GACCUCACCAUCAGUAUUA<br>CAGGCAACCUCAACAUGAA
BBS7	NM_018190	UAACAGGCCAGUUCAGUUU<br>UUACAGGGAUCUGAUGUGA<br>GCGCUUAUACAGAUUACUA<br>GUUCUACGAUUUGAUCAGA
CCNT1	NM_001240	UAUCAACACUGCUAUAGUA<br>GAACAAACGUCCUGGUGAU<br>GCACAAGACUCACCCAUCU<br>UAGUAUACAUGCAUCGAUU
CNTN3	NM_020872	GCUCAAAGUUGUUGCUUCU<br>GAGUUCAUCUGGUGAUUUA<br>GCUGAGAAUUCACGAGGAA<br>GAAGAUAGUCGGAGAUUUG
DMD	NM_000109	GAAUGUUUAUGAUACGGGA<br>GCAAGUGGCAAGUUCAACA<br>GUCAGAUUCUCAGCUUAUA<br>CAAGACAGUUGGGUGAAGU
DNAH5	NM_001369	GGACAUCACUGAAUAUUGA<br>AAACUGAGCUUGUACAUGA<br>GAAGUUCCCUGCCCUAAUU<br>CGAAUGAGAUAGUGCGACA
DOCK11	NM_144658	UAAAUGAGCGGCUAAUUAA<br>GAUCAACACCGACAGUUUA<br>CGACAGAGUUCUACAAGGA<br>CGAAACAGAUCAGGAGUAA
DSCAM	NM_001389	GCGCAUAAGUGAAAUCAUA<br>GGACGGGAUUUAUGGCAAA<br>ACGGGAGCCUUGUAUAUUA<br>GUCAAAGCACGCACAAUUA
EPB41L3	NM_012307	GCUCGAAUAUCAGCAAUUA<br>GAAAGUAUGUGUUGAGCAU<br>CGGAACAACUUUUACAUUA<br>GGGCUUACGUAUCGAGAUG
FLNA	NM_001456	CCAACAAGGUCAAAGUAUA<br>GCAGGAGGCUGGCGAGUAU<br>GUAUGGAGAUGAAGAGGUA<br>UCACAGAAAUUGACCAAGA
GDF5	NM_000557	GGAAGGCACUGCAUGUCAA<br>AUAAGACCGUGUAUGAGUA<br>CCAAUAAGCUUGCUCGCUC<br>ACGUGGUGUAUAAGCAGUA
HDLBP	NM_005336	CACCAUCGCUUUGUUAUUG<br>GAAGCGACACCGUUGUUAU<br>CGGCAAAGCCAUUCGCAAA<br>GGUUGGAGCAUGACGUGAA
KDM5B	NM_006618	CCACAGAGCUUGUUGAGAA<br>GCAGAGGCCAUGAAUAUUA<br>CAACAGAACCUCAUAUUUG<br>GGAGAUGCACUUCGAUAUA
MYO3A	NM_017433	GAAAGGCAAUAACAUUCUA<br>GCAAUUGGAUACCACUUAU<br>GAACAGCAAUUGGAUACCA<br>GAAGAGGGGUGAAAGAAUG
MYO3B	NM_138995	GAAAUGAUGUUUACACCAA<br>GUACAUAGCUGAUGAAACU<br>GGAAAGGUAUUAUAUGAUG<br>GGGCAAGAAUCUCUGAAUA
MYCBP2	NM_015057	GGAGUAAGCUGACUAACUU

		GGACAGGUCUUCACAUUUG<br>GAAAUGAGAUAUCUACGUU<br>GAAGGAAGCUCGAUAUAUU
NALCN	NM_052867	GCUCAUCUCUCCACAAAUU<br>GAUAGGAACUAGUAUAUUC<br>GGACUUCGGUGGAGUAAUG<br>GGACAGAUUUACUACGUUU
NFATC2	NM_013986	CCAAUAAUGUCACCUCGAA<br>GCAGAAUCGUCUCUUUACA<br>GCGGGGAUCUUGAAGCUUA<br>UCAUGUACUGCGAGAAUUU
PCDH11X	NM_014522	GACCUUAACUUGUCGCUGA<br>CCAGAGAACUCGGCUAUAA<br>GGAAUAAACGGAGUUCAAA<br>GCAAGUGAGUGUUACUGAU
PIK3C2B	NM_002646	GCCGGAAGCUUCUGGGUUU<br>CAAGAGCUCUGGCCGAAUC<br>GCUGAGACCCUGCGUAAGA<br>GCUACCAGCUAUGAAGAUU
PKHD1	NM_138694	CGAGAUAGCUGUACUUUCA<br>CCAAUUACCGUCAAGAUUA<br>GGUAUGGUCUCUUUGUAUA<br>GGAAGGUGUUAGCCUGAUA
RABGAP1	NM_012197	GAUGGAAAGUCGUAUGUUA<br>GAAUUAGCCUGCAACAUGA<br>GUAGAGAAAUACCGCAUUC<br>AGAAAUGUGCCGUCGGGAA
RNF17	NM_031277	GAACUAUGCUUGUUAAUGA<br>GCAAGAGCAUUACAAUUAU<br>GAACUUUCUUGUUACGAUA<br>GAAAUUAGACAGAAUGUUC
SYCP2L	NM_001040274	GAAGAUGAACCUCUGCUAA<br>CCUCGUAGAUGGCCUGAAA<br>GGACUGAUCCCAAAGCUAG<br>UGUGUAGACUGACGAUUAA
SLC30A2	NM_001004434	GAGCAUGGGUGUCCUAGUG<br>GAGAAGUCGUUGAGAUCUU<br>GAGAUGUGAUCCUGGUGUU<br>CUGCAUAUCUGGGCACUGA
TP53	NM_000546	GAGGUUGGCUCUGACUGUA<br>GCACAGAGGAAGAGAAUCU<br>GAAGAAACCACUGGAUGGA<br>GCUUCGAGAUGUUCCGAGA
UGGT1	NM_020120	GAACAGAUCUGAAAGAGUU<br>CAAGGGAACUUUAGGAUUA<br>CAAAGGAGAUCACAGAUAU<br>GCUAGACCCUGAUGAGUUA
XIRP2	NM_152381	CAACAGAAGCUGCCCGCAA<br>GAAGAGAUGUGGAGUUCGA<br>GCGCAGGAUUGAACGCUUU<br>GAAAUUACUUCUUCCCGUA
ZFHX3	NM_006885	GUAUAAACCAAACGAGUUA<br>GUACAGAGACCACUACGAU<br>CCACUAUGCUAGAAUGUGA<br>GAACAAGGUUUACGGACUA

Qiagen GeneSolution

Gene Symbol	Accession Number	Target Sequence
BCL9L	NM_182557	CCCGACGGCUACCACCGCCAA*<br>CAGAGCUGAAGCCAUAUACGA*(3'utr)<br>CACCUGCAACGUGGGCUCGAA*<br>ACCCACAAUUGUAAUGUAGCA
BID	NM_001196	CAGGGAUGAGUGCAUCACAAA<br>AAAGACAAUGUUAAACUUAUA<br>UAGGGACUAUCUAUCUUAAUA<br>AACUUCCAAUUUCUAGCCAUA
CASP2	NM_032982	AACAUCUUCUGGAGAAGGACA*<br>AACCGAGUGGUGCUAGCCAAA*<br>UUGGUCCACCUUCCAGCACAA<br>CAGGAUCAUGUAAAUGCUCAA*
ZFHX3	NM_006885	CGGCGUGAGCUUACAAAUGAU<br>AACGGACACCUUCAGAUUGUA<br>CAGAGCGCUCUUCAAAGCCAA<br>AAGAACAAGGUUUACGGACUA

(*). Oligonucleotides with the same effect as the pool of 4 and used in deconvolution experiments

Reversine-tolerance assays

Cell-viability and BrdU assays were performed by seeding cells in transfection medium with reversine 250 nM for 72 hr. For cell viability, 20 µl of Cell Titer Blue (Promega) was added in 100 µl of culture media for 2 hr and fluorescence measured according to manufacturers instructions. For BrdU analysis, BrdU was added to a final concentration of 100 nM for 1 hr. Cells were trypsinized, fixed in 70% ethanol, washed with PBS, resuspended in 2 M HCl for 20 min and washed twice in PBS. Fixed cells were then stained with anti-BrdU primary antibody (1/50, Becton-Dickinson) washed with PBS and finally stained with secondary goat anti-mouse Alexa Fluor488-conjugated antibody (Life Technologies). Finally cells were resuspended in 1 ml of PBS, treated with 50 µl of ribonuclease A (100 mg/ml stock, Sigma-Aldrich) and 150 ul of propidium iodide (50 µg/ml stock, Sigma-Aldrich) and subjected to FACS analysis.

For colony formation assays cells were transfected with siRNA for 3 days, then plated 6 serial 1/10 dilutions at a starting concentration of $2x10^5$ cells (6-well plates) in 250 nM reversine for 5 days. Reversine-containing medium was replaced by drug free medium and colonies were allowed to grow to an appropriate size. For long-term treatment (CRISPR and shRNA) experiments we used reversine 125 nM for 15 days.

Caspase 3/7 enzymatic activity assay

For determination of caspase3/7 enzymatic activity we used the ApoOne system (Promega) according to manufacturers instructions.

Endogenous segregation error screen (Live-cell imaging)

For live cell imaging, we generated stable HCT-116 expressing H2B-RFP. 48 hr siRNA knock-down was carried out on microscopy-compatible cell chambers. Imaging was performed using a DeltaVision RTsystem (Image Solutions, Applied Precission) equipped with temperature, humidity and CO2 control. Time-lapse images were acquired for each point with z-stacks steps of 2 μm to image the whole cell, every 3 min for 6 hr, which allows adequate spatio-temporal resolution to identify segregation errors. Images were acquired every 15 min during the rest of the experiment up to 48 hr to determine if daughter cells divided further, arrested or died.

Segregation error analysis

Cells were grown on poly-lysine (1/8 dilution, Sigma-Aldrich) coated coverslips and fixed in 10% Triton X-100, 1M PIPES, 0.5M EGTA, 1M $MgCl_2$, 4% formaldehyde. Coverslips were stained with rabbit anti-β-tubulin antibody (1/1000, Abcam), human anti-centromere antibodies (1/250, Antibodies Incorporated) and DAPI (Roche). For secondary staining we used goat anti-rabbit Alexa Fluor 488 and goat anti-human Alexa Fluor 594 (Molecular Probes, 1/500). All antibodies were diluted in 3% BSA 1xPBS. Manual examination of segregation errors in anaphase was carried out with z-stacks acquired with an Olympus DeltaVision RT microscope (Applied Precision) at 60x or 100x.

Western-blot and quantitative PCR

Cell lysates were made with a buffer containing 100 mM NaCl, 50 mM Tris, 10% glycerol and 0.2% NP40 and protein concentration measured with Bradford reagent. Protein electrophoresis and transfer onto PVDF membrane were carried out using NuPAGE NOVEX system (Life Technologies). Electrophoresis was performed with NuPAGE Bis-Tris 4-12% gradient gels in 1x NuPAGE MES running buffer and transferred for 90 min at 150V in 1x NuPAGE transfer buffer, 20% methanol. For BID detection we used 12% NuPAGE Bis-Tris non-gradient gels and for BCL9L detection we used NuPAGE 3-8% Tris-Acetate gels and transfer with pH10 transfer buffer (19.2 mM Tris, 2.5 mM Glycine, 0.01% SDS and 10% methanol). Primary antibodies used were mouse anti-p53 (1/1000, DO1 clone, Santa Cruz Biotechnology), mouse anti-MDM2 (1/1000, SMP14 clone, Santa Cruz Biotechnology), rabbit anti-C-terminal MDM2 (1/1000, C18, Santa Cruz Biotechnology), rat anti-caspase-2 antibody (1/1000, Enzo Life Technologies), rabbit anti-BCL9L (1/500, Abcam), rabbit anti-BID (1/1000, ab113110, Abcam) and HRP-conjugated anti-β-actin (1/50000, A3854, Sigma-Aldrich), rabbit anti-PARP (1/1000, 46D11, Cell Signaling Technology). For primary antibody detection we used secondary HRP-swine anti-rabbit (1/50000, Dako), HRP-goat anti-mouse (1/50000, Dako) and HRP-goat anti-rat (1/10000, GE Healthcare Life Sciences).

Taqman gene-expression assays (Applied Biosystems): *GAPDH* (Hs99999905), *BID* (Hs01026792), *CASP2* (Hs00892481), *PIDD* (Hs00611142).

PCR primers for SYBR green quantitative PCR:

AXIN2_F	gctgacggatgattccatgt
AXIN2_R	actgcccacacgataaggag
BAD_F	cgagtttgtggactcctttaaga
BAD_R	caccaggactggaagactcg

BAK1_F	cctgccctctgcttctga
BAK1_R	ctgctgatggcggtaaaaa
BAX_F	caagaccagggtggttgg
BAX_R	cactcccgccacaaagat
BBC3_F	gacctcaacgcacagtacga
BBC3_R	gagattgtacaggaccctcca
BCLXL_F	agccttggatccaggagaa
BCLXL_R	agcggttgaagcgttcct
BCL9L_1F	tggattcagaggccaaagag
BCL9L_1R	cccactgtacggctgctt
BIM_F	catcgcggtattcggttc
BIM_R	gctttgccatttggtcttttt
CASP3_F	tgtgaggcggttgtagaaga
CASP3_R	gggctcgctaactcctcac
CASP7_F	ccgagacttttagtttcgcttt
CASP7_R	cctgatcatctgccatcgt
CASP8_F	ggtcacttgaaccttgggaat
CASP8_R	tttctgctgaagtccatcttttt
CASP9_F	aagcccaagctctttttcatc
CASP9_R	actcgtcttcaggggaagtg
CDKN1A_F	ccgaagtcagttccttgtgg
CDKN1A_F	catgggttctgacggacat
MCL1_F	Aagccaatgggcaggtct
MCL1_R	tgtccagtttccgaagcat
MYC_F	gctgcttagacgctggattt
MYC_R	taacgttgaggggcatcg
TP53_F	aggccttggaactcaaggat
TP53_R	cccttttggacttcaggtg

Subcellular compartment protein enrichment.

For enrichment of nuclear and cytoplasmic protein fractions we used Qproteome Cell Compartment Kit (Qiagen) according to manufacturers instructions.

CRISPR genome editing and reversine-resistant colony selection

We designed a guide RNA construct targeting nucleotides 2542-2561 (AGTACTACGAAGAGAAACGG) of *BCL9L* (cDNA sequence NM182557) according to protocol described by Cong and colleagues (2013) by cloning a protospacer sequence into pX330-hSpCas9. transient transfection system (double-strand break). Cells were transfected with the *BCL9L* targeting construct for 48 hr and seeded in serial dilutions in reversine-containing media. After 2 weeks in 125 nM reversine resistant colonies were collected and *BCL9L* genotyped by Sanger sequencing.

Chromatin-immunoprecipitation

A 15 cm dish of 70% confluent HCT-116 was fixed with 1% formaldehyde in culture medium for 10 min at room temperature and quenched by adding 2.14 ml of 1.5 M glycine. Cells were scraped in 10 ml PBS with protease and phosphatase inhibitors and pelleted at 1500 rpm for 10 min at 4°C. The cell pellet was resuspended in 2 ml of buffer A containing NP40 (5 mM PIPES-NaOH, pH8, 85 mM KCl, 0.5% NP40 and inhibitors), incubated with rotation at 4°C for 10 min and pelleted at 3700 rpm for 10 min at 4°C. The pellet was resuspended buffer A without NP40, incubated with rotation at 4°C for 10 min and pelleted at 3700 rpm for 10 min at 4°C. The pellet was resuspended in lysis buffer B (50 mM Tris-HCl, pH8, 1% SDS, 10 mM EDTA and inhibitors), incubated with rotation at 4°C for 10 min and pelleted at 3700 rpm for 10 min at 4°C. Samples were sonicated in a Bioruptor at low intensity for 5 cycles (30 seconds on/30 seconds off), incubated at 4°C for 10 min and pelleted at 3700 rpm for 10 min at 4°C. The chromatin solution was diluted 1/5 with IP buffer (16.7 mM Tris-HCl, pH8, 167 nM NaCl, 1.2 mM EDTA, 1.1% Triton X100, 0.01% SDS and inhibitors). The chromatin solution was pre-cleared with 30 µl of Protein G-Sepharose FastFlow beads (Sigma) for 1 hr at 4°C. Sepharose beads were pelleted and 2 ml of the chromatin solution were incubated with 40 µl of rabbit antiTCF4 antibody (C48H11, Cell Signaling Technology) or with an equivalent amount of rabbit IgG (2729, Cell Signaling Technology) simultaneously with 30 µl of Protein G-Sepharose beads at 4°C overnight. Beads were washed 3 times with a low salt buffer (20 nM Tris HCl, pH8, 150 mM NaCl, 1% Triton X-100 and 0.1% SDS) and once with high salt buffer (same composition except 300 nM NaCl). Immune complexes were eluted overnight in 250 µl of elution buffer (10 mM Tris-HCl, pH8, 300 mM NaCl, 5 mM EDTA, 1% SDS) at 65°C. The supernatant was incubated 2 µl RNAase A (100 mg/ml) for 1 hr at 37°C followed by 2 µl Proteinase K (10 mg/ml) at 65°C for 2 hr and finally DNA purified with Qiagen PCR purification columns. Enrichment of immunoprecipitated DNA in the *CASP2* promoter was studied by qPCR using the following primers:

Primer pairs designed upstream the TCF4 binding site in the CASP2 promoter (ENCODE, chr7:142,985,169-142,985,544):

1F	ATGGGTTGGTATGAAGGCCA
1R	AGCTGTTAGAGACTCTTCGCA
2F	TGCCTCACTCCTAAGCTCTG
2R	GCAGCACACAATTCCAGGAA
3F	ATCTGTCCAGGGCCTTGTC
3R	TGGCGATAACTTCACGGAGA

Primers designed within the TCF4 binding site:

4F	TCTCAGTCTCCTTCGCAGC
4R	CCTATGGCGATAACTTCACGG
5F	AATCGGATTCCCAGGCCG
5R	CAGACAAAAGGAGGGGAAGG
6F	GTCTGAGGGGAGGGATGTG
6R	CCATTTCCCGCTTTTCCCG
7F	AAAAGCGGGAAATGGCGG
7R	TTCCCTACTTACCTGCGTCC

Primer pairs designed downstream the region of TCF4 binding site in the CASP2 promoter (5'UTR region and coding sequence):

8F	ATGGCCGCTGACAGGGGACG
8R	GCAGGAAGCGCGGACACG
9F	CAAGGGTGGGACGCCCGC
9R	CTGCAAAGACCTCCCCTTTAG
10F	CGACGAGACCTTCTGCAGAC
10R	GTACTCAATTTCCAAACCC

Mouse xenografts

HCT-116 cells infected with lentiviral shRNA targeting human *BCL9L* and control shRNA were grown in 125 nM reversine for 15 days and surviving colonies were trypsinized and expanded for two weeks. Two million cells were injected subcutaneously into nude (*nu/nu*) mice according to the UK Home Office rules and regulations.

Statistical methods

Data was analyzed using Prism 6.0 by two-tailed Student's t-test and Fisher's exact test. For the analysis shown in Figure S2B, on the basis of the observed probability for loss, given by the percentage of genome that is lost in that sample, we generated an aberration state (loss or no loss) for each sample separately. A pointbiserial correlation between aberration state and wGII was then calculated across samples. This process was repeated 10,000 times and a p value was obtained for *BCL9L* by counting the percentage of permutations showing a greater correlation coefficient than that observed for *BCL9L*. For statistical analysis of tissue FISH analysis, centromere counts as established by FISH were tabulated and difference in ploidy was assessed using a two-tailed Fisher's exact test. All analyses were performed in R 2.12.2, with Monte Carlo simulation used to compute the p values for the Fisher's test. For statistical analysis of metaphase spreads, total chromosome numbers were analyzed by two-sided Wilconxon rank sum test and performed in R 3.2.3.

SUPPLEMENTAL REFERENCES

Bocking, A., Giroud, F., and Reith, A. (1995). Consensus report of the ESACP task force on standardization of diagnostic DNA image cytometry. European Society for Analytical Cellular Pathology. Analytical cellular pathology : the journal of the European Society for Analytical Cellular Pathology *8*, 67-74.

Cong, L., Ran, F. A., Cox, D., Lin, S., Barretto, R., Habib, N., Hsu, P. D., Wu, X., Jiang, W., Marraffini, L. A., and Zhang, F. (2013). Multiplex genome engineering using CRISPR/Cas systems. Science *339*, 819-823.

Dunn, J. M., Mackenzie, G. D., Oukrif, D., Mosse, C. A., Banks, M. R., Thorpe, S., Sasieni, P., Bown, S. G., Novelli, M. R., Rabinovitch, P. S., and Lovat, L. B. (2010). Image cytometry accurately detects DNA ploidy abnormalities and predicts late relapse to high-grade dysplasia and adenocarcinoma in Barrett's oesophagus following photodynamic therapy. British journal of cancer *102*, 1608-1617.

Haroske, G., Baak, J. P., Danielsen, H., Giroud, F., Gschwendtner, A., Oberholzer, M., Reith, A., Spieler, P., and Bocking, A. (2001). Fourth updated ESACP consensus report on diagnostic DNA image cytometry. Analytical cellular pathology : the journal of the European Society for Analytical Cellular Pathology *23*, 89-95.
